# Mitofusin-Mediated Mitochondrial Fusion Inhibits Pseudorabies Virus Infection in Porcine Cells

**DOI:** 10.3390/vetsci12040368

**Published:** 2025-04-15

**Authors:** Xiuhan Xu, Yuan Zhao, Zhenbang Zhu, Wei Wen, Xiangdong Li

**Affiliations:** 1 Jiangsu Co-Innovation Center for Prevention and Control of Important Animal Infectious Diseases and Zoonoses, College of Veterinary Medicine, Yangzhou University, Yangzhou 225009, China; 2 Joint International Research Laboratory of Agriculture and Agri-Product Safety, The Ministry of Education of China, Yangzhou University, Yangzhou 225009, China; 3 Key Laboratory of Protection & Utilization of Biological Resources in Tarim Basin, College of Life Sciences, Tarim University, Alar 843300, China

**Keywords:** pseudorabies virus, mitochondrial dynamics, mitochondrial fusion, mitofusin proteins

## Abstract

**Background:** Mitochondria are highly dynamic organelles that undergo fusion/fission dynamics, and emerging evidence has established that mitochondrial dynamics plays a crucial regulatory role in the process of viral infection. Nevertheless, the function of mitochondria dynamics during pseudorabies (PRV) infection remains uncertain. **Methods:** Our investigation commenced with examining PRV-induced alterations in mitochondrial dynamics, focusing on morphological changes and the expression levels of fusion/fission proteins. We then restored mitochondrial dynamics through Mfn1 (Mitofusin 1)/Mfn2 (Mitofusin 2) overexpression and mdivi-1 (mitochondrial division inhibitor-1) treatment to assess their impact on PRV replication and mitochondrial damage. **Results:** We found a downregulation of the mitochondrial fusion proteins Mfn1, Mfn2, and OPA1 (optic atrophy 1), along with the activation of the fission protein Drp-1 (dynamin-related protein 1) upon PRV infection. Restoring the function of mitochondrial fusion inhibited PRV infection. Furthermore, elevated mitochondrial membrane potential (MMP), decreased reactive oxygen species (ROS) levels, and an increased mitochondrial number were observed after overexpressing Mfns or treatment with mdivi-1. **Conclusions:** PRV infection impairs mitochondrial dynamics by altering mitochondrial fusion and fission proteins, and the promotion of Mfn-mediated mitochondrial fusion inhibits PRV replication.

## Simple Summary

Mitochondrial dynamics has been shown to play a role in viral replication processes. This study aimed to systematically elucidate the role of mitochondrial dynamics in modulating pseudorabies virus (PRV) infection and its underlying mechanisms. Our results demonstrate that PRV infection in PK-15 cells induced significant alterations in mitochondrial morphology, accompanied by the downregulation of key mitochondrial fusion proteins, including mitofusin 1 (Mfn1), mitofusin 2 (Mfn2), and optic atrophy 1 (OPA1), while simultaneously activating the mitochondrial fission protein dynamin-related protein 1 (Drp1). Through overexpression of Mfn1 and Mfn2 or treatment with mdivi-1 (mitochondrial division inhibitor-1), we observed significant suppression of PRV glycoprotein B (gB) expression and subsequent inhibition of viral replication. Further investigation revealed that both Mfn overexpression and mdivi-1 treatment significantly attenuated reactive oxygen species (ROS) generation, restored mitochondrial membrane potential (MMP), and increased the mitochondrial number during PRV infection.

## 1. Introduction

Pseudorabies virus (PRV) is a linear double-stranded DNA virus belonging to the subfamily α-herpesviruses. Infection with PRV is characterized by the presence of fever, pruritus, and neurological signs in a variety of domesticated and wild animals [1,2,3]. The range of mammals susceptible to infection by PRV is extensive, including dogs, rabbits, and cattle. Furthermore, pigs represent the sole natural host for PRV [4]. The economic impact of PRV infection on the global swine farming industry is significant, with estimated losses reaching millions of dollars annually. In China, pigs were immunized with gene-deleted PRV live vaccines prior to 2011 [5], which resulted in the effective control of the disease. However, with the emergence of PRV variants, the outbreak of this disease spread in many vaccinated pig farms [6,7,8,9]. Recent studies have demonstrated that PRV variants can also infect humans, resulting in significant neurological damage, which raises concerns about the potential for cross-species transmission [10,11].

Mitochondria are highly dynamic organelles that undergo fusion/fission dynamics [12]. Mitochondrial dynamics are crucial for the maintenance of their biological functions [13]. Disruption to mitochondrial dynamics typically results in mitochondrial fragmentation and the subsequent impairment of cellular function [14,15]. A number of viruses have been demonstrated to facilitate self-replication by disrupting mitochondrial dynamics [16]. For example, Zika virus (ZIKV) has been demonstrated to suppress type I interferon (IFN) response by disrupting mitochondrial dynamics [17]. Newcastle disease virus (NDV) shifts the balance of mitochondrial dynamics from fusion to fission, activating mitophagy [18]. Changes in mitochondrial dynamics have also been reported during herpesvirus infection. Human herpesvirus type 1 (HHV-1, also known as herpes simplex virus type 1, HSV-1) and type 2 (HHV-2, also known as herpes simplex virus type 2, HSV-2) induce fragmentation in the mitochondrial morphology and decrease in the mitochondrial membrane potential, disrupting mitochondrial dynamics [19]. HSV-1 infection promotes mitochondrial fission and inhibits mitochondrial fusion, ultimately hampering mitochondrial dynamics [20]. In the existing research, PRV infection has been shown to disrupt mitochondrial motility and morphology via the Ca^2+^-sensitive cellular protein Miro, which impairs the recruitment of kinesin-1 to mitochondria. This disruption of mitochondrial dynamics is required for the efficient growth and spread of PRV [21]. A recent study conducted by our research group demonstrated that infection with PRV resulted in impaired mitochondrial dynamics [22]. Specifically, PRV infection induced mitochondrial fragmentation and mitophagy, thereby inhibiting the IFN-β response and promoting viral replication. These findings revealed a previously uncharacterized PRV infection mechanism that impairs mitochondrial dynamics [22]. Nevertheless, the precise role of mitochondrial dynamics in viral infections remains uncertain.

Mitofusins (Mfns) are widely distributed on the outer mitochondrial membrane (OMM) and are responsible for OMM fusion [23]. They are indispensable for the maintenance of normal mitochondrial function, acting by regulating mitochondrial fusion. In mammals, mitofusins can be divided into two types: mitofusin 1 (Mfn1) and mitofusin 2 (Mfn2) [24]. Mfns are key proteins in the regulation of mitochondrial dynamics [25]. An increasing number of studies have demonstrated that Mfn-mediated mitochondrial fusion plays a critical role in disease genesis. For example, Mfn2-mediated mitochondrial dynamics have been demonstrated to prevent cerebral ischemia/reperfusion injury [26]. Impaired mitochondrial dynamics induced by Mfn2 downregulation have been identified as a contributing factor in the disease process of nonalcoholic fatty liver disease (NAFLD) [27]. Mfns maintain mitochondrial dynamics in human immunodeficiency virus type 1 (HIV-1) infection by inhibiting MMP disruption and subsequent apoptosis [28]. However, few reports explain the roles of Mfn1 and Mfn2 in viral infection. In the case of DENV infection, Mfn1 is responsible for the host’s antiviral response, which inhibits viral replication. Mfn2, on the other hand, is responsible for mitigating cell death induced by viral infection. Furthermore, DENV facilitates self-replication by cleaving the Mfns protein, thereby inhibiting the host antiviral response [29]. It remains unclear whether Mfns plays a role in PRV and other herpesvirus infections.

In this study, we demonstrate that PRV infection resulted in impaired mitochondrial fusion and enhanced fission. Restoration of the mitochondrial fusion function by overexpressing Mfn1 or Mfn2 resulted in the inhibition of PRV replication. This study reveals the mechanism by which PRV infection disrupts mitochondrial dynamics and demonstrates the key role of Mfns proteins in anti-PRV functions.

## 2. Materials and Methods

### 2.1. Cells and Virus

PK-15 cells were obtained from the American Type Culture Collection and cultured in Dulbecco’s modified Eagle medium (HyClone, SH30243.01, Logan, UT, USA) containing 10% fetal bovine serum (Lonsera, S711-001S, Suzhou, China) and 100 U/mL penicillin/0.1 mg/mL streptomycin at 37 °C with 5% CO_2_.

Recombinant virus rPRV HN1201-EGFP-Luc (G-PRV) was provided by Prof. Beibei Chu at Henan Agricultural University [30]. Briefly, the recombinant virus was generated by co-transfection of the donor plasmid pUC57-US6/7-EGFP-Luc-US8/9, plasmid PX459M-sgRNA1-sgRNA2, and genomic DNA of the parental strain PRV HN1201 into PK-15 cells. The wild-type PRV JS-2020 strain (W-PRV) was stored in our laboratory [31].

### 2.2. Plasmids

Mfn2-MYC and Mfn1-MYC expression plasmids were constructed by inserting the corresponding swine ORF into the pcDNA3.1-MYC vector. All plasmids were verified by DNA sequencing. JetPRIME Transfection Reagent was employed for all transfection assays according to the manufacturer’s protocol.

### 2.3. RNA Interference

For RNA interference-based knockdown experiments, *Mfn1* siRNA (5′-GCGACUUUCCAAGCCUAAUTT-3′ and 3′-AUUAGGCUUGGAAAGUCGCTT-5′) and *Mfn2* siRNA (5′-GCUUUCAAGUGAGGAUGUUTT-3′ and 3′-AACAUCCUCACUUGAAAGCTT-5′) were purchased from Genepharma (Suzhou, China). PK-15 cells were transfected with 50 nM siRNA (final concentration) together with JetPRIME Transfection Reagent. RNAi Negative Control (Genepharma) was used as the control.

### 2.4. Chemical Reagents

The following reagents were used in this study: Enhanced Mitochondrial Membrane Potential Assay Kit with JC-1 (Beyotime, C2003S, Shanghai, China), Reactive Oxygen Species Assay Kit (Beyotime, S0033S, Shanghai, China), phenylmethanesulfonyl fluoride (PMSF), (Beyotime, ST506, Shanghai, China), chloroquine (CQ), (MCE, HY-17589A, Monmouth Junction, NY, USA), MG132 (Selleck, S2619, Houston, TX, USA), Z-VAD (MCE, HY-16658B, Monmouth Junction, NY, USA), mdivi-1 (Beyotime, SC8028, Shanghai, China), and JetPRIME Transfection Reagent (Ployplus, 101000046, Illkirch-Graffenstaden, France).

The following antibodies were used in this study: Myc-Tag (CST, 2276, Danvers, MA, USA), MFN1 (CST, 14739, Danvers, MA, USA), MFN2 (Proteintech, 12186-1-AP, Rosemont, IL, USA), TOMM20 (Santa Cruz, sc-271583, Dallas, MA, USA), COX4 (CST, 4850, Danvers, MA, USA), GAPDH (Proteintech, 60004-1-Ig, Rosemont, IL, USA), DRP1 (CST, 8570, used for Western blot, Danvers, MA, USA), phospho-Drp1 Ser616 (CST, 4494, Danvers, MA, USA), and OPA1 (CST, 80471, Danvers, MA, USA). The gB monoclonal antibody was a generous gift from Prof. Beibei Chu at Henan Agricultural University [30].

### 2.5. Western Blot

Cells were collected and lysed using RIPA buffer with 1 mM PMSF added (Beyotime, P0013B, Shanghai, China) to prepare total lysates. After detecting the protein concentration using the bicinchoninic acid (BCA) method, the protein samples were separated by SDS-PAGE gels and then transferred to polyvinylidene fluoride (PVDF) membranes (Pall Corporation, 66543, Port Washington, NY, USA). The membranes were blocked for 60 min (room temperature) using 5% skimmed milk (Sangon Biotech, A600669, Shanghai, China) in Tris-buffered saline (TBS) with 0.1% Tween-20 (TBS-T) (Sangon Biotech, A600560, Shanghai, China). The primary antibody was then incubated at 4 °C, the secondary antibody was incubated at room temperature, and they were finally developed using chemiluminescent substrate. The protein bands were detected on Tanon 5200 Multi (China) using an ECL Kit (NCM Biotech, P10300, Nanjing, China) and quantified by Image J analysis. The density of each band was normalized to its respective loading control.

### 2.6. RNA Extraction and qRT-PCR

Total RNA was extracted with TRIzol reagent (Tiangen, DP424, Beijing, China) and subjected to reverse transcription with HiScript III RT SuperMix for qPCR (Vazyme, R323–01, Nanjing, China). The DNA copy number was quantified through qRT-PCR by using specific primers: Mfn1-F: GCTGGTCATCCCTTGTCCAT, Mfn1-R: GCTAAAGATCTGGGGAGCTGA, Mfn2-F: TGGAGTCAACGCAATCAGCA, Mfn2-R: CAGGGACATCGCGTTTTTGG, OPA1-F: AGACTTTTTCACCACAGGTTCAC, OPA1-R: ATGAGCTCACCAAGCAGACC. Subsequently, the qRT-PCR reaction program was set up and initiated in the instrument (Thermo Fisher Scientific, Waltham, MA, USA) with the following parameters: 95 °C for 30 s (initial denaturation), followed by 40 cycles of 95 °C for 10 s and 60 °C for 30 s.

### 2.7. Immunofluorescence Assay (IFA)

PK-15 cells were infected with W-PRV (1 moi) for the indicated times and then fixed in 4% paraformaldehyde for 10 min, permeabilized with 0.1% Triton X-100 (Solarbio, T8200, Beijing, China) in PBS (Vazyme, G101, Nanjing, China) for 15 min, blocked with 2% bovine serum albumin (Solarbio, A8020, Beijing, China) in PBS for 1 h at room temperature, and incubated with the primary antibodies at 4 °C overnight. After washing with PBS, the cells were incubated with the secondary antibodies at RT for 1 h, followed by staining with DAPI (Beyotime, C1002, Shanghai, China). Finally, the slides were observed under a confocal microscope (ZEISS, LSM 880NLO, Oberkochen, Germany) with a 100× oil immersion objective.

### 2.8. Flow Cytometry

PK-15 cells were incubated with 10 μM JC-1 probe for 25 min at 37 °C (for the mitochondrial membrane potential assessment) or with 10 μM DCFH-DA probe for 20 min at 37 °C (for the ROS level assessment), and then the fluorescence intensity was detected by flow cytometry (Beckman Coulter, CytoFLEX S, Brea, CA, USA) in three independent experiments.

Under normal mitochondrial membrane potential (MMP) conditions, JC-1 is multimeric and fluoresces red. When the MMP is depolarized, JC-1 is monomeric and fluoresces green. The level of MMP was assessed by calculating the red–green fluorescence ratio.

### 2.9. Viral Titer Assays

PK-15 cells were seeded in 96-well plates and treated with serial diluted samples from virus supernatant dilutions. After 72 h of incubation, the Reed and Muench method was used to calculate the 50% tissue culture infectious dose (TCID_50_).

### 2.10. Viral Replication Levels Assays

PK-15 cells were infected with G-PRV (1 moi) for the indicated times, IFA experiments were then performed to assess the level of viral replication. In order to quantify the data, each group first calculated their respective GFP/DAPI ratios by Image J, and then these ratios were compared across the groups.

### 2.11. Statistical Analysis

The results are expressed as the mean ± SD values. Significant differences among the groups were determined with one-way analysis of variance followed by a Tukey test using statistical software GraphPad Prism 9.0. Values of * *p* < 0.05, ** *p* < 0.01, and *** *p* < 0.001 were considered significant, and *ns* indicates a lack of significant difference.

## 3. Results

### 3.1. PRV Infection Impairs Mitochondrial Dynamics

**Figure 1:**
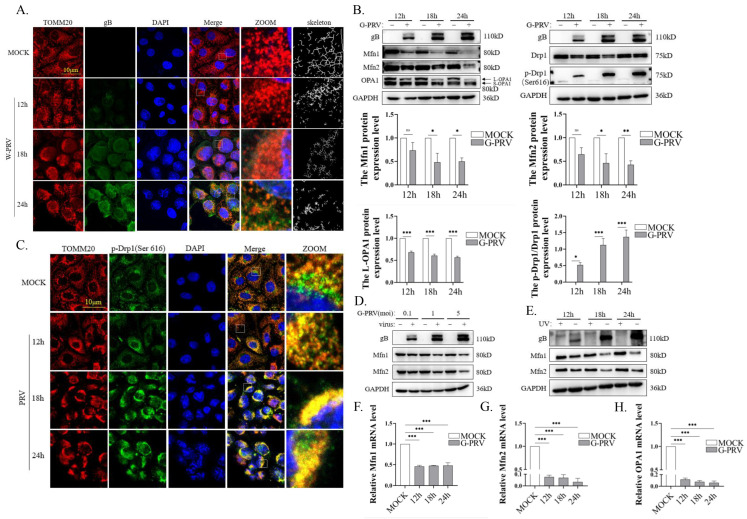
PRV infection impairs mitochondrial dynamics (Western Blot original figures see Supplementary Materials). (**A**) Confocal microscopy of PK-15 cells infected with W-PRV (moi 1) for 12, 18, and 24 h. The mitochondria network was analyzed by Image J. Green: anti-gB; red: anti-TOMM20; blue: DAPI. Scale bar: 10 μm. The yellow boxes indicated these areas were enlarged on the right. (**B**) Western blot analysis and quantification of expression levels of PK-15 cells infected with G-PRV (moi 1) for 12, 18, and 24 h. (**C**) Confocal microscopy of PK-15 cells infected with W-PRV (moi 1) for 12, 18, and 24 h. Green: anti-p-Drp1; red: anti-TOMM20; blue: DAPI. Scale bar: 10 μm. The yellow boxes indicated these areas were enlarged on the right. (**D**) Western blot analysis and quantification of expression levels of PK-15 cells infected with G-PRV (moi 0.1, 1, and 5) for 24 h. (**E**) Western blot analysis of PK-15 cells infected with G-PRV (moi 1) for 12, 18, and 24 h. G-PRV was pre-treated with UV or natural light (control) for 30 s. (**F**–**H**) The mRNA levels of indicated genes were detected by RT-qPCR. Data are mean ± SD values (n = 3 per group). *, **, and *** indicate statistically significant differences with *p* < 0.05, *p* < 0.01, and *p* < 0.001, respectively. ns indicates not statistically significant.

To investigate the impact of PRV infection on mitochondrial dynamics, we examined the mitochondrial morphology in PK-15 cells during PRV infection using confocal microscopy. We observed significant mitochondrial fragmentation during PRV infection (Figure 1A), consistent with our previous findings [22]. Since mitochondrial fusion and fission processes are regulated by dynamic proteins, we next analyzed the expression of these mitochondrial fusion and fission proteins in PK-15 cells at various time points post-PRV infection. The expression levels of mitochondrial fusion proteins, including Mfn1 and Mfn2, were significantly decreased at 18 and 24 h post-PRV infection (hpi) (Figure 1B left). Optic atrophy 1 protein (OPA1) localizes on the inner mitochondrial membrane (IMM) and mediates IMM fusion. Due to differential RNA splicing and precursor protein processing, OPA1 exists as distinct isoforms. The long isoform (L-OPA1) has a membrane anchor, but the short isoform (S-OPA1) lacks a membrane anchor [32]. Only the long isoform promotes mitochondrial fusion under conditions of stress [33]. The expression levels of L-OPA1 were significantly downregulated at 12, 18, and 24 hpi (Figure 1B left). Dynamin-related protein 1 (Drp1) plays a central role in the mitochondrial fission process and is located mainly in the cytoplasm. Once recruited to the mitochondria and phosphorylated at serine 616, Drp1 initiates the fission process [34]. The phosphorylation level of the mitochondrial fission protein Drp-1 at ser 616 was significantly increased. (Figure 1B right), as was the co-localization of p-Drp1 (Ser 616) with mitochondria (Figure 1C). Moreover, PRV infection suppressed the protein expressions of Mfn1 and Mfn2 in a dose-dependent manner (Figure 1D). However, UV-treated PRV lost the ability to affect Mfn1 and Mfn2 expression (Figure 1E). The mRNA levels of Mfn1, Mfn2, and OPA1 were similarly downregulated at 12–24 hpi (Figure 1F–H). Overall, mitochondrial dynamics were disrupted by PRV infection through the downregulation of mitochondrial fusion protein expression and the promotion of fission protein activation.

### 3.2. Promotion of Mitochondrial Fusion Inhibits PRV Replication

**Figure 2:**
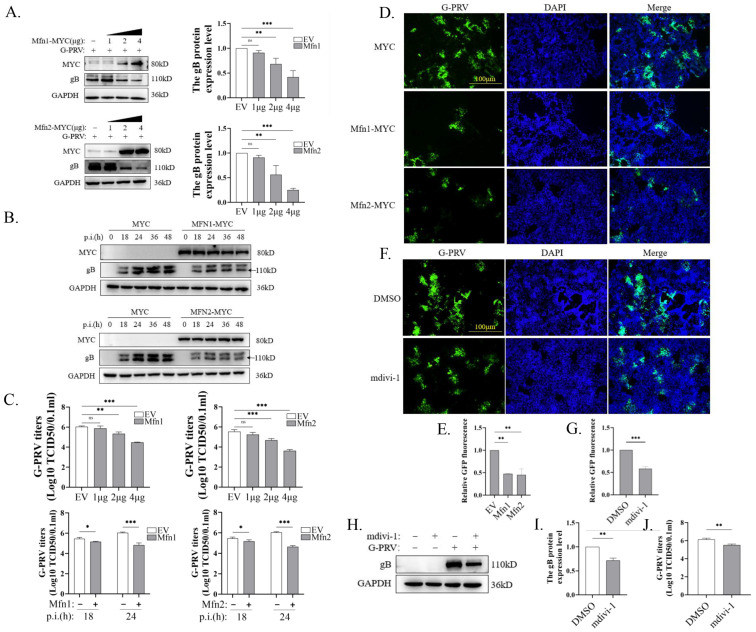
The promotion of mitochondrial fusion inhibited PRV replication (Western Blot original figures see Supplementary Materials). (**A**,**B**) Western blot analysis and quantification of expression levels of PK-15 cells. Cells were transfected with EV, Mfn1 or Mfn2 plasmids for 24 h and then infected with G-PRV (1 moi) for (**A**) 24 h, (**B**) 18, 24, 36, or 48 h. (**C**) Analysis of the culture supernatants derived from panel A to B for PRV titer. (**D**–**G**) The levels of G-PRV replication (green) were visualized by an inverted fluorescence microscope upon G-PRV (1 moi) infection at 24 h. The GFP/DAPI fluorescence intensity ratios of each group were compared to quantify the data. Scale bar: 100 μm. (**H**,**I**) Western blot analysis and quantification of gB expression levels. Cells were infected with G-PRV (1 moi) for 24 h in the absence or presence of mdivi-1 (25 µM). (**J**) Analysis of the culture supernatants derived from panel H for PRV virus titer. Data are mean ± SD values (n = 3 per group). *, **, and *** indicate statistically significant differences with *p* < 0.05, *p* < 0.01, and *p* < 0.001, respectively. ns indicates not statistically significant.

Viruses promote self-replication by regulating a variety of cell biological events through a range of strategies. The function of mitochondrial fusion has been demonstrated to confer protection against a number of diseases and viral infections [13,16,27]. Therefore, we sought to examine whether the restoration of mitochondrial fusion function could influence the course of PRV infection by overexpressing Mfn1 and Mfn2.Mfn1 or Mfn2 expression plasmids were transfected into PK-15 cells along with PRV infection. The expression of PRV gB (glycoprotein B) protein was found to be decreased by the overexpression of Mfn1 or Mfn2 in a dose-dependent manner (Figure 2A). Similarly, the overexpression of Mfn1 or Mfn2 downregulated gB protein expression from 18 to 48 hpi (Figure 2B). Through TCID_50_ experiments, we also found that the overexpression of Mfn1 or Mfn2 downregulated PRV virus titers in dose- and time-dependent manners (Figure 2C). We then infected PK-15 cells with G-PRV and found that virus titers were decreased when cells were transfected with Mfn1 or Mfn2 plasmids (Figure 2D,E). Mdivi-1 is a widely used inhibitor of mitochondrial fission [35].To simulate the restoration of mitochondrial fusion function achieved by Mfn overexpression, we treated PRV-infected PK-15 cells with mdivi-1. Consistent with this approach, mdivi-1 inhibited PRV replication, as demonstrated by the IFA (Figure 2F,G), Western blot (Figure 2H,I), and TCID_50_ (Figure 2J) results.

### 3.3. Blocking Mitochondrial Fusion Promotes PRV Infection

**Figure 3:**
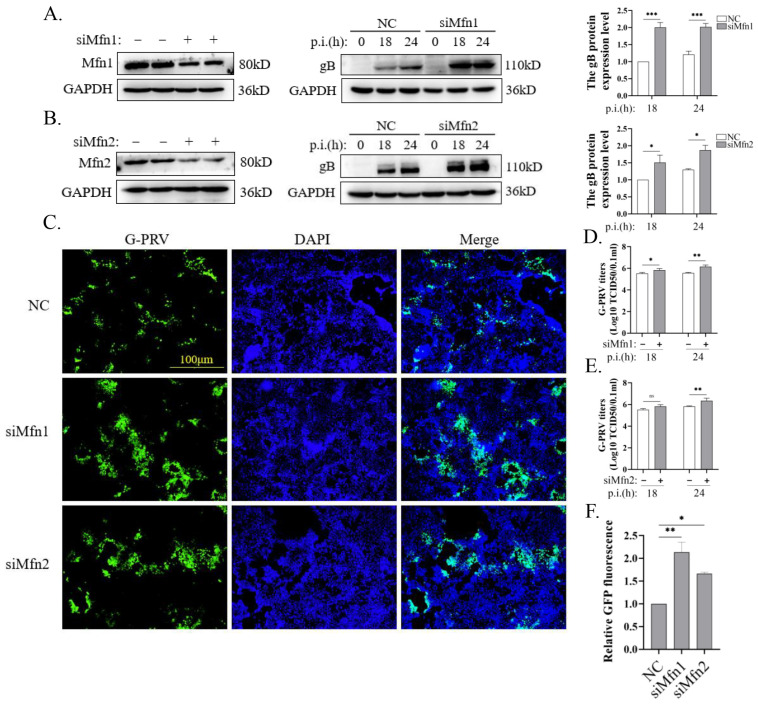
Blocking mitochondrial fusion promoted PRV replication (Western Blot original figures see Supplementary Materials). (**A**,**B**) Western blot analysis and quantification of different protein expression levels of PK-15 cells. Cells were transfected with NC or siMfn1 or siMfn2 siRNAs for 24 h and then infected with G-PRV (moi 1) for 18 and 24 h. (**D**,**E**) Analysis of the culture supernatants derived from panel A to B for PRV titer. (**C**–**F**) The levels of G-PRV replication (green) were visualized by an inverted fluorescence microscope (OLYMPUS, DP74) upon G-PRV (1 moi) infection at 24 h. The GFP/DAPI fluorescence intensity ratios of each group were compared to quantify the data. Scale bar: 100 μm. Data are mean ± SD values (n = 3 per group). *, **, and *** indicate statistically significant differences with *p* < 0.05, *p* < 0.01, and *p* < 0.001, respectively. ns indicates not statistically significant.

To further validate the inhibition of PRV replication through Mfn-mediated mitochondrial fusion, we designed siRNAs targeting *Mfn1* or *Mfn2*. The expressions of the corresponding proteins were successfully knocked down in PK-15 cells, as shown in Figure 3A,B (left). The expression of PRV gB protein was increased via the knockdown of *Mfn1* or *Mfn2* in PK-15 cells infected with G-PRV at 18 and 24 hpi (Figure 3A,B, middle and right), along with the increased GFP fluorescence intensity and virus titer (Figure 3C–F). These data demonstrate that Mfn proteins are necessary for the inhibition of PRV replication.

### 3.4. Promotion of Mitochondrial Fusion Alleviates Mitochondrial Damage and Decreases Mitochondrial Number Caused by PRV Infection

**Figure 4:**
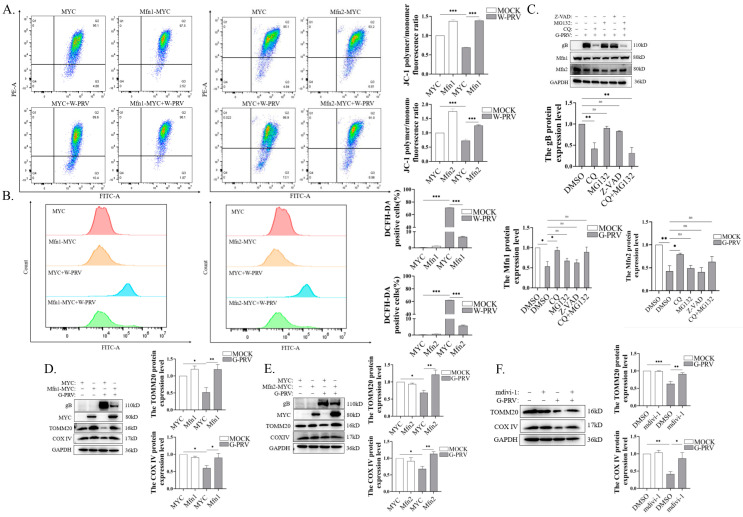
The promotion of mitochondrial fusion alleviated mitochondrial damage and decreased the mitochondrial number caused by PRV infection (Western Blot original figures see Supplementary Materials). (**A**,**B**) Flow cytometry with JC-1 (**A**) or DCFH-DA (**B**) staining of PK-15 cells. Cells were transfected with EV or Mfn1 or Mfn2 plasmids for 24 h and then infected with W-PRV (1 moi) for 24 h. Decreased red/green fluorescence ratio of JC-1 represents disrupted MMP. (**C**) Western blot analysis and quantification of the expression levels of PK-15 cells. Cells were infected with G-PRV (1 moi) for 24 h in the absence or presence of CQ (50 μM), MG132 (10 μM), or Z-VAD (10 μM). (**D**,**E**) Western blot analysis and quantification of TOMM20 and COX IV expression levels of PK-15 cells. Cells were transfected with EV or Mfn1 or Mfn2 plasmids for 24 h and then infected with G-PRV (moi 1) for 24 h. (**F**) Western blot analysis and quantification of TOMM20 and COX IV expression levels of PK-15. Cells were infected with G-PRV (1 moi) for 24 h in the absence or presence of mdivi-1(25 μM). *, **, and *** indicate statistically significant differences with *p* < 0.05, *p* < 0.01, and *p* < 0.001, respectively. ns indicates not statistically significant.

In our previous study, mitochondrial damage (shown by decreased mitochondrial membrane potential and elevated reactive oxygen species) and a decreased mitochondrial number (shown by the decreased expressions of TOMM20 and COX IV proteins) were observed in PK-15 cells with impaired mitochondrial dynamics during PRV infection [22]. This indicates the existence of a potential correlation between mitochondrial damage and impaired mitochondrial dynamics in PRV infection. Moreover, given that the expression of mitochondrial fusion proteins was suppressed in PRV infection, we postulated that Mfns may exert a regulatory effect on mitochondrial damage and the mitochondrial number in PRV infection. To test this hypothesis, Mfns were overexpressed in PK-15 cells before PRV infection, and then the mitochondrial membrane potential (MMP) and reactive oxygen species (ROS) levels were evaluated. The overexpression of Mfns resulted in a notable recovery in MMP levels and a considerable reduction in ROS (Figure 4A,B). Furthermore, in order to detect the pathways by which PRV downregulates Mfn expression, the mitophagy inhibitor CQ, the proteasome inhibitor MG132, and the apoptosis inhibitor Z-VAD-FMK were employed. As shown in Figure 4C, only CQ was able to reverse the downregulation of Mfn proteins induced by PRV infection, indicating that PRV degrades Mfns via mitophagy. Notably, the gB protein levels were inhibited due to CQ treatment (Figure 4C), which is consistent with our previous findings [22]. Considering that CQ treatment may enhance the function of the proteasome [36], the cells were treated with CQ+MG132 to exclude the effect of the proteasome on PRV replication, and the data showed that the level of gB protein expression of PRV was significantly decreased (Figure 4C). The TOMM20 and COX IV expression levels were significantly increased due to the overexpression of Mfns in PRV-infected PK-15 cells (Figure 4D,E). Similarly, treatment with mdivi-1 resulted in increases in TOMM20 and COX IV. (Figure 4F). Taken together, the above data suggest that Mfn-mediated fusion functions alleviated PRV-induced mitochondrial damage and reduced mitochondrial numbers.

### 3.5. Blocking Mitochondrial Fusion Enhances PRV-Induced Mitochondrial Damage

**Figure 5:**
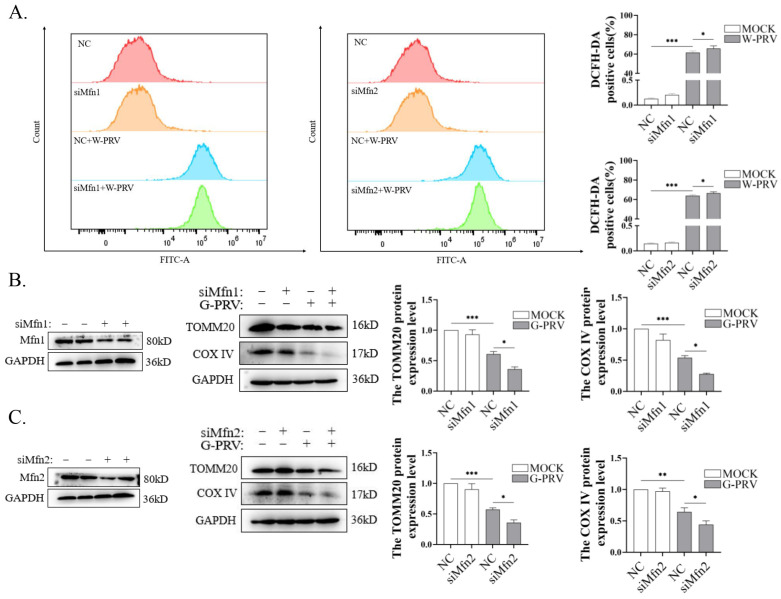
Blocking mitochondrial fusion enhanced PRV-induced mitochondrial damage and mitophagy (Western Blot original figures see Supplementary Materials). (**A**) Flow cytometry with DCFH-DA staining of PK-15 cells. Cells were transfected with NC, siMfn1, or siMfn2 siRNAs for 24 h and then infected with W-PRV (1 moi) for 24 h. (**B**,**C**) Western blot analysis and quantification of Mfn1, Mfn2, TOMM20, and COX IV expression levels in PK-15 cells. Cells were transfected with NC or siMfn1 or siMfn2 siRNAs for 24 h and then infected with G-PRV (1 moi) for 24 h. *, **, and *** indicate statistically significant differences with *p* < 0.05, *p* < 0.01, and *p* < 0.001, respectively.

To further explore the role of Mfns in alleviating PRV-induced mitochondrial damage, Mfns were knocked down by siRNAs. Enhanced ROS levels induced by PRV infection were detected when *Mfn1* or *Mfn2* were knocked down (Figure 5A). Moreover, the expressions of TOMM20 and COX IV were further downregulated due to the knockdown of Mfns after PRV infection (Figure 4B,C). The above data suggest that Mfn-mediated mitochondrial fusion function could mitigate mitochondrial damage and reduced the mitochondrial numbers during PRV infection.

## 4. Discussion

Viruses are completely dependent on the resources of the host cell for replication. In order to obtain a cellular environment conducive to replication, viruses strategically regulate biological events in the host cell, resulting in dramatic changes in cellular and subcellular structure and function [37,38].Mitochondria serve as pivotal hubs for cellular energy metabolism and are increasingly recognized as crucial platforms for antiviral immune signaling. Viruses also strategically alter the mitochondrial dynamics to promote their replication [16]. In this study, we demonstrated that PRV infection disrupted the mitochondrial fusion/fission balance by degrading mitochondrial fusion proteins and promoting the activation of fission proteins.

Mitochondrial fusion and division are the direct factors affecting its morphology and function. Under normal conditions, damaged mitochondria are separated from the health ones, and remaining healthy parts fuse back into the complete mitochondrial network. Upon viral infection, the number of damaged mitochondria increases. These damaged mitochondria are segregated from healthy ones more rapidly than under normal conditions, resulting in insufficient numbers of healthy mitochondria available for reintegration into the mitochondrial network. Consequently, the balance of mitochondrial dynamics is disrupted, and mitophagy is activated to clear virus-damaged mitochondria [39]. Hepatitis B virus (HBV) and hepatitis C virus (HCV) infection subvert mitochondrial dynamics via the promotion of fission and activate mitophagy to support viral persistence [40,41]. Classical swine fever virus (CSFV) infection induces mitochondrial fission and, resultantly, mitophagy to promote viral replication [42]. However, dengue virus (DENV) disturbs mitochondrial dynamics by inducing mitochondrial elongation and inhibiting mitochondrial fission, as evidenced by the decreased phosphorylation of Drp1 at ser 616, which supports viral replication [43]. In our study, PRV infection decreased mitochondrial fusion protein (Mfn1, Mfn2, and OPA1) expression and promoted mitochondrial fission protein Drp1 phosphorylation at ser 616 and mitochondrial localization, which is consistent with the IFA results showing a broken mitochondria network. This indicates that PRV disturbed the balance of mitochondrial dynamics via the promotion of mitochondrial fission (Figure 1), suggesting that maintaining the fusion process may do harm to viral persistence [40,41]. To explore the effects of mitochondrial dynamics on PRV replication, we utilized two methods to restore mitochondrial fusion, including the overexpression of Mfn1 and Mfn2, and treatment with a mitochondrial fission inhibitor, mdivi-1. We found that restoring mitochondrial fusion inhibited viral replication, and blocking mitochondria fusion with the knockdown of Mfn1 and Mfn2 was beneficial to viral replication (Figure 2 and Figure 3). Mdivi-1 has been reported to play a regulatory role in herpesvirus replication. Mdivi-1 treatment intensified HSV-1 infection-induced impaired mitophagy and promoted HSV-1 replication in neuronal cells and mouse brain tissue [20]. Human herpesvirus 8 (HHV-8) infection promotes mitochondrial clearance by activating mitophagy to support virus replication, and mdivi-1 inhibits the production of HHV-8 virions [44]. Our study further supports the role played by mdivi-1 in herpesvirus infection. Our data also show that Mfn-mediated mitochondria fusion was crucial for the inhibition of PRV proliferation. Notably, PRV downregulated the mRNA levels of Mfn1 and Mfn2, which may play an important role in PRV replication. It has been demonstrated that transcription factors regulate the stability of target genes and inhibit the transcription process, thus playing an important regulatory role in viral replication [45]. In addition, it has been reported that PRV’s immediate early protein IE180 affects the cascade of gene expression for the development of the murine cerebellum [46]. Future studies should take these two directions fully into account, thus offering the possibility of clarifying the core mechanisms by which PRV infection regulates mitochondrial dynamics.

Subsequently, we investigated the pathway through which PRV downregulates Mfn1 and Mfn2 protein levels. By using inhibitors targeting three distinct pathways, we demonstrated that PRV degrades Mfn1 and Mfn2 via the mitophagy pathway. However, Mfn1 and Mfn2 have different effects in different virus infections. During influenza virus (H1N1) infection, Mfn2 was reported to accelerate the polyubiquitination and degradation of mitochondrial antiviral signaling protein MAVS, impairing IFN-I production and thereby promoting viral replication [47]. Furthermore, human immunodeficiency virus type 1 (HIV-1) infection increased Mfn1 and Mfn2 protein levels, protecting against MMP depolarization, which contributes to inhibiting apoptosis and supporting viral proliferation [28]. In our previous study, we found that PRV infection induced mitophagy to promote the mitophagic degradation of MAVS, thereby inhibiting IFN-I production and facilitating viral replication [22]. Since mitochondrial dynamic imbalance precedes the occurrence of mitophagy, we hypothesized that promoting mitochondrial fusion to inhibit PRV replication may be associated with the suppression of mitophagy initiation. Indeed, our data show that the restoration of mitochondrial fusion alleviated the degree of mitochondrial damage and inhibited mitophagy (Figure 4 and Figure 5). It should be noted that our study has certain limitations. Although we have demonstrated that PRV degrades Mfn1 and Mfn2 through the mitophagy pathway, the precise molecular mechanism requires further elucidation, and the key PRV-encoded proteins responsible for this phenomenon remain to be identified in future studies. Additionally, existing research has reported that Mfns can be cleaved by certain viral proteins [29], so this possibility should be considered in future investigations of PRV infection. Nevertheless, our study provides evidence that PRV infection induces Mfn degradation via mitophagy, highlighting the crucial role of mitophagy in PRV replication and offering novel insights into the mechanisms by which PRV regulates mitochondrial dynamics.

In summary, we have demonstrated that PRV infection blocks mitochondrial fusion by decreasing Mfn protein levels and restoring fusion inhibits viral replication. These findings offer a new direction for PRV prevention and control. Furthermore, a novel example of Mfns functioning as anti-PRV agents has been provided, but the targets through which Mfns function require further investigation in order to provide a theoretical basis for vaccine development. Notably, the conclusions of this study were primarily derived from the PK-15 cell model. Although this cell line is widely used for in vitro PRV research [48,49], its divergence from the natural host environment during infection warrants careful interpretation. For instance, PK-15 cells lack intact immune regulation and organ-specific microenvironments, which may not fully recapitulate the physiological role of mitochondrial dynamics in PRV-infected animals. Further investigations using PRV-infected animal models are required to validate the function of mitochondrial fusion under physiological conditions. While we acknowledge that this limitation may affect the generalizability of our findings, our study still provides a foundational framework for understanding the molecular interplay between PRV and host mitochondria.

## Data Availability

All relevant data are included in the article.

## Supplementary Materials

The following supporting information can be downloaded at: https://www.mdpi.com/article/10.3390/vetsci12040368/s1.
