# Supplementary material for: Mitofusin-Mediated Mitochondrial Fusion Inhibits Pseudorabies Virus Infection in Porcine Cells
Source: Vet Sci. 2025 Apr 15;12(4):368. doi: 10.3390/vetsci12040368 (PMC12030837; doi:10.3390/vetsci12040368)

Manuscript ID: vetsci-3517192

Western Blot origin image

Figure S1.B(left)

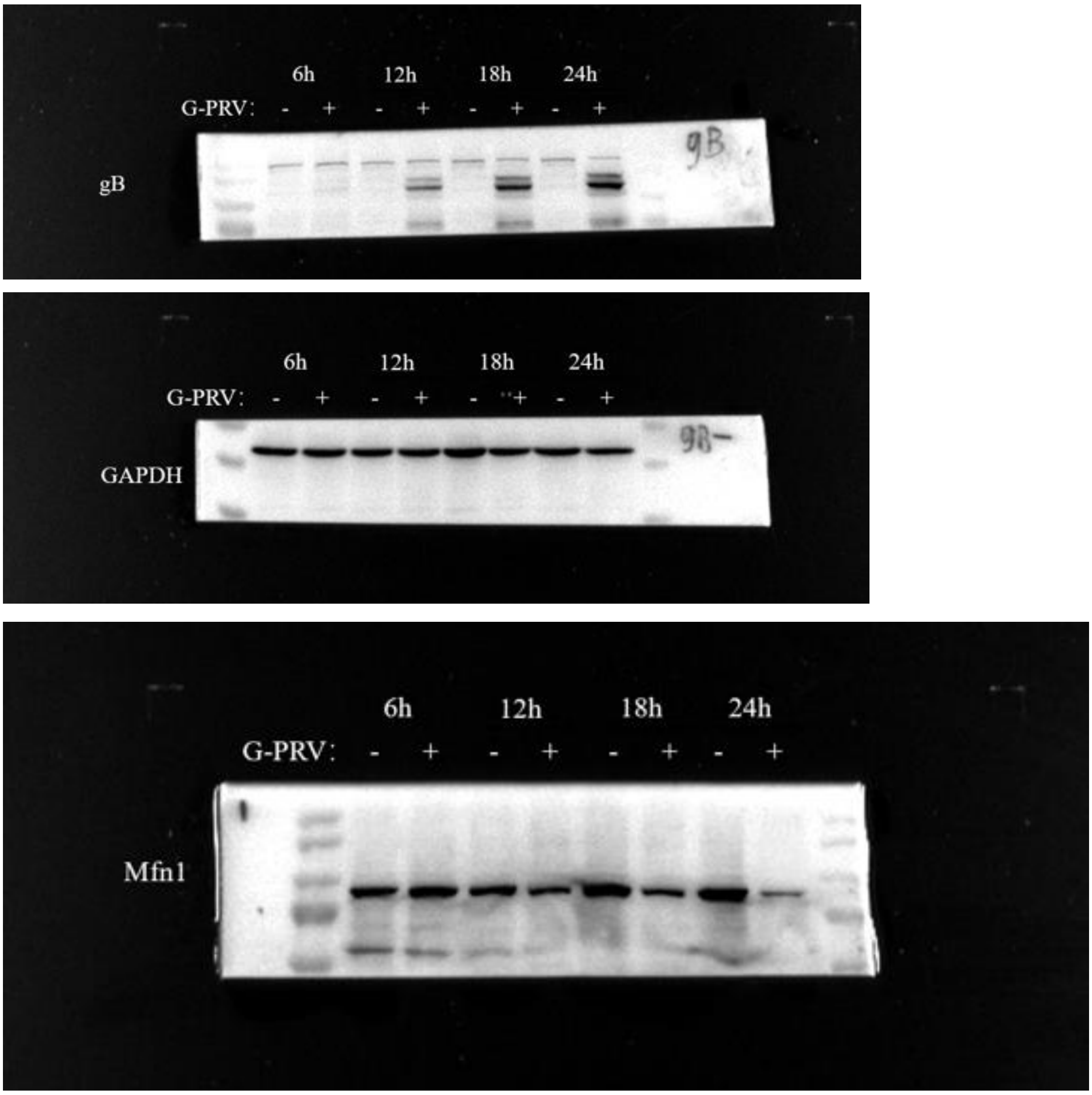

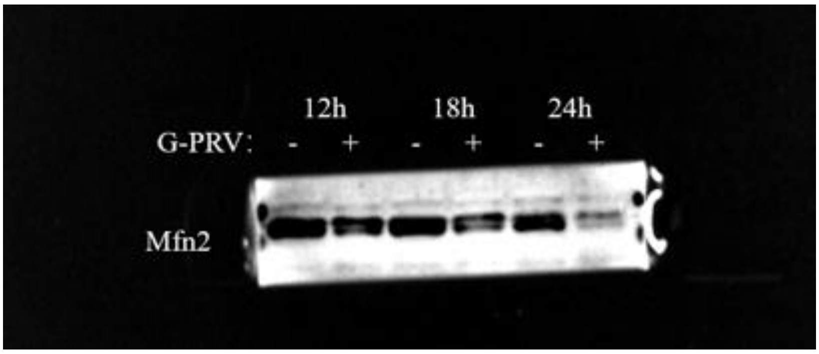

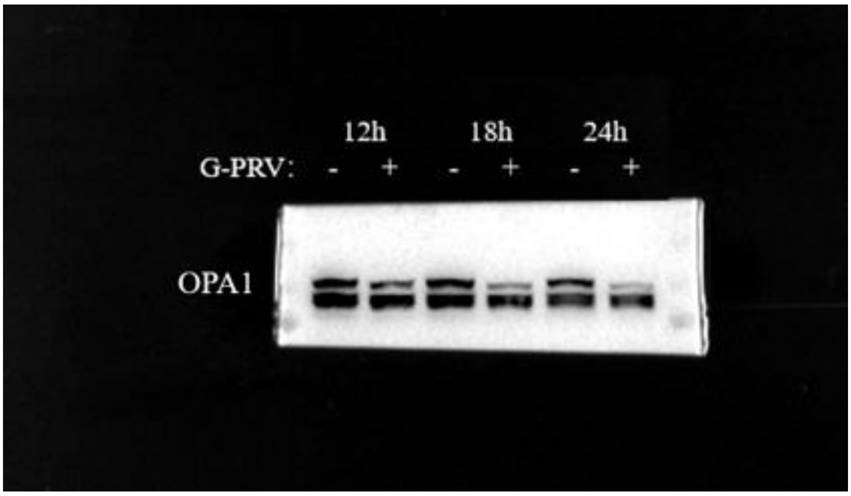

Figure S1.B(left) repeat 2

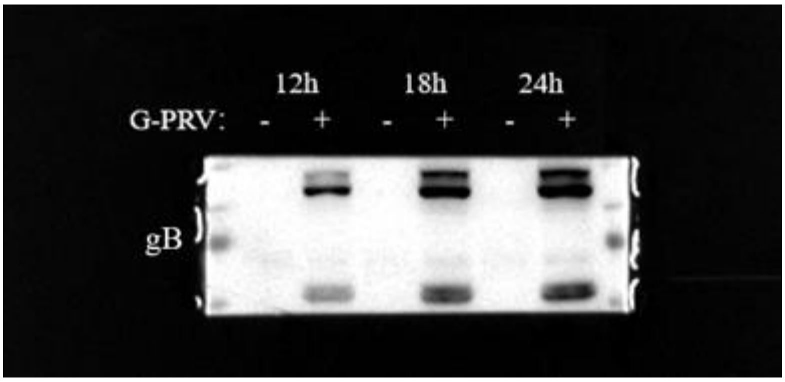

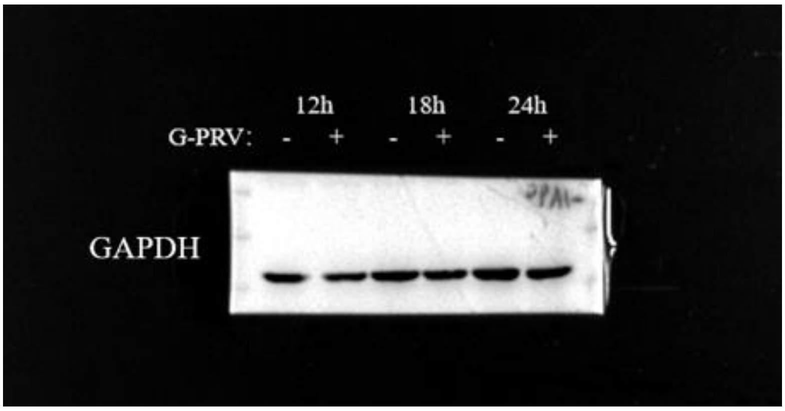

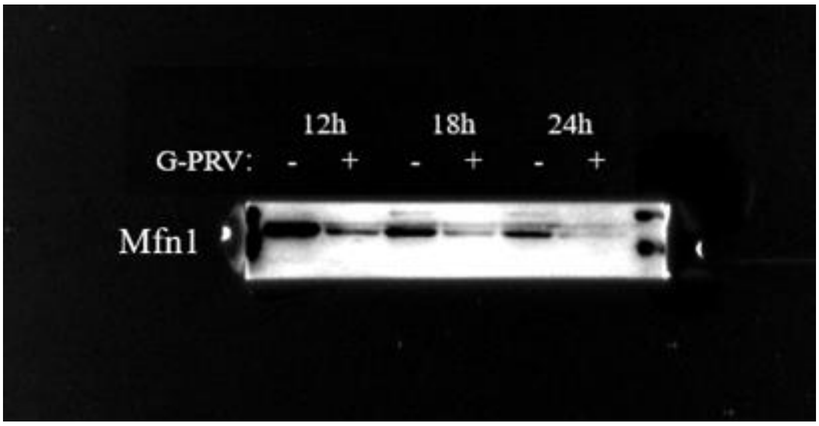
12h 18h 24h
G-PRV: - + - + - +
Mfn1

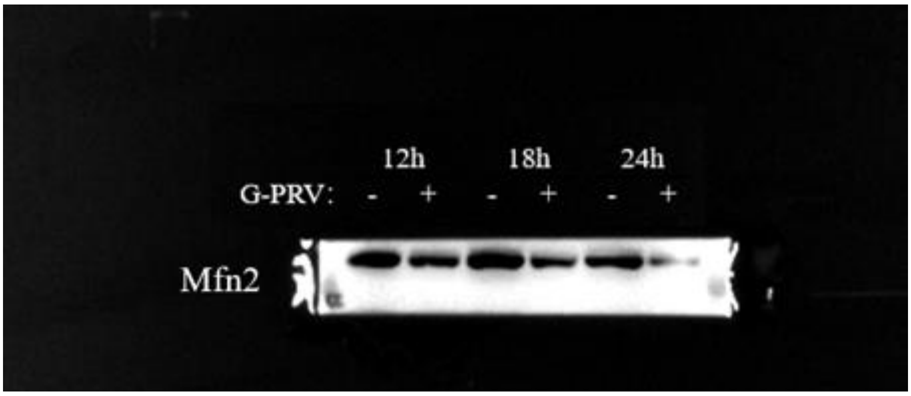
12h 18h 24h
G-PRV: - + - + - +
Mfn2

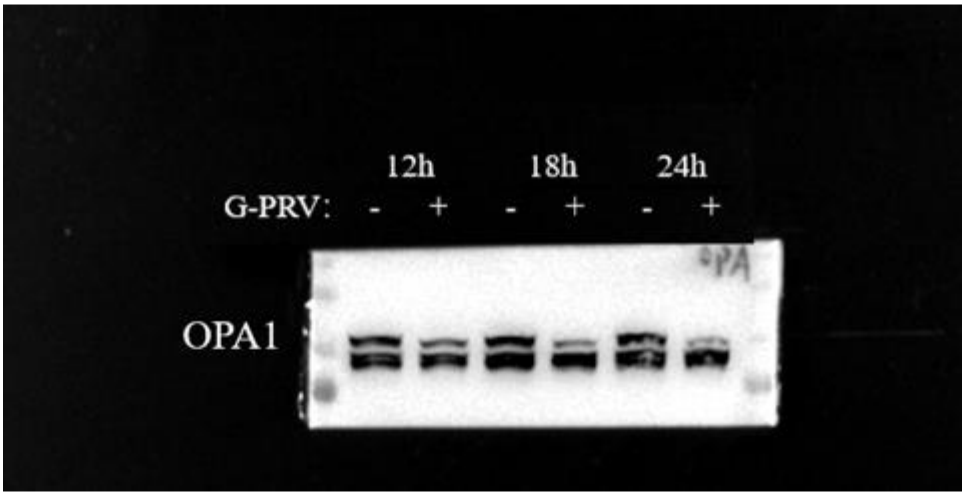
12h 18h 24h
G-PRV: - + - + - +
OPA1

Figure S1.B(left) repeat 3

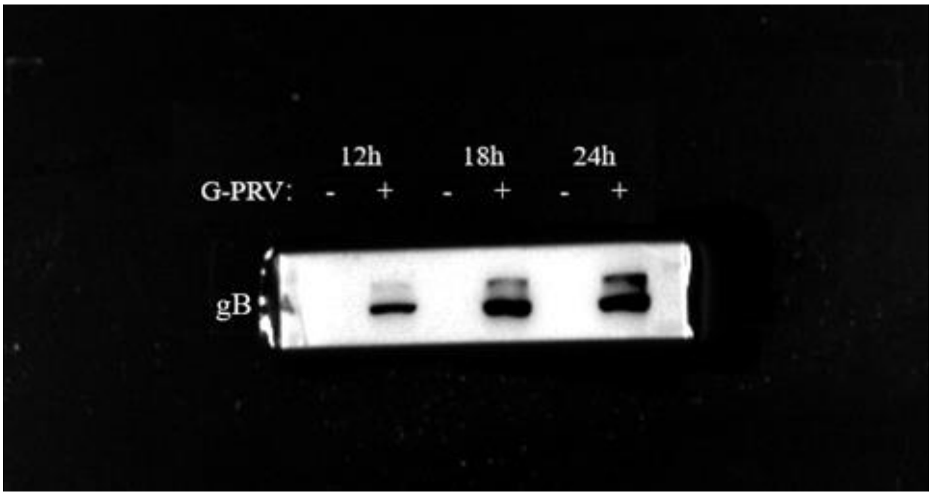

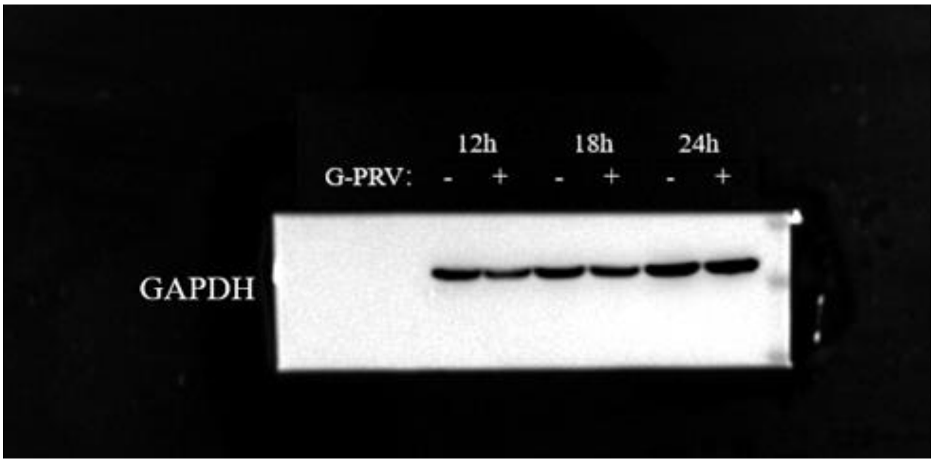

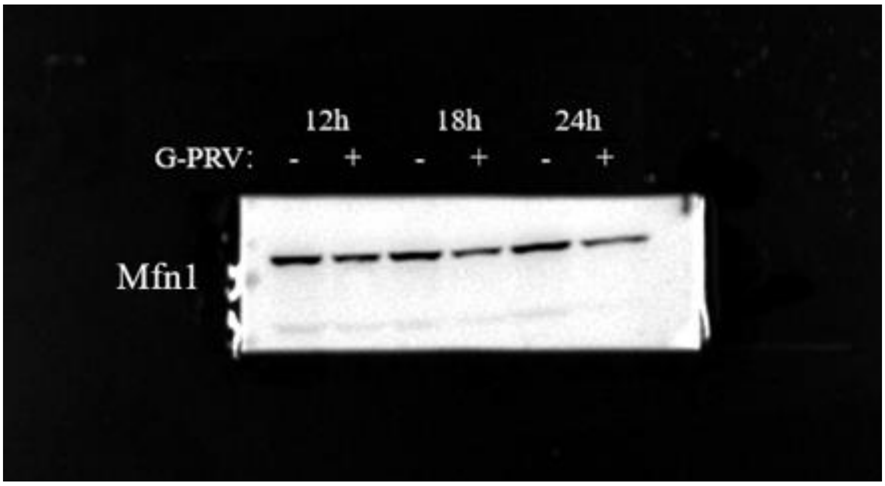

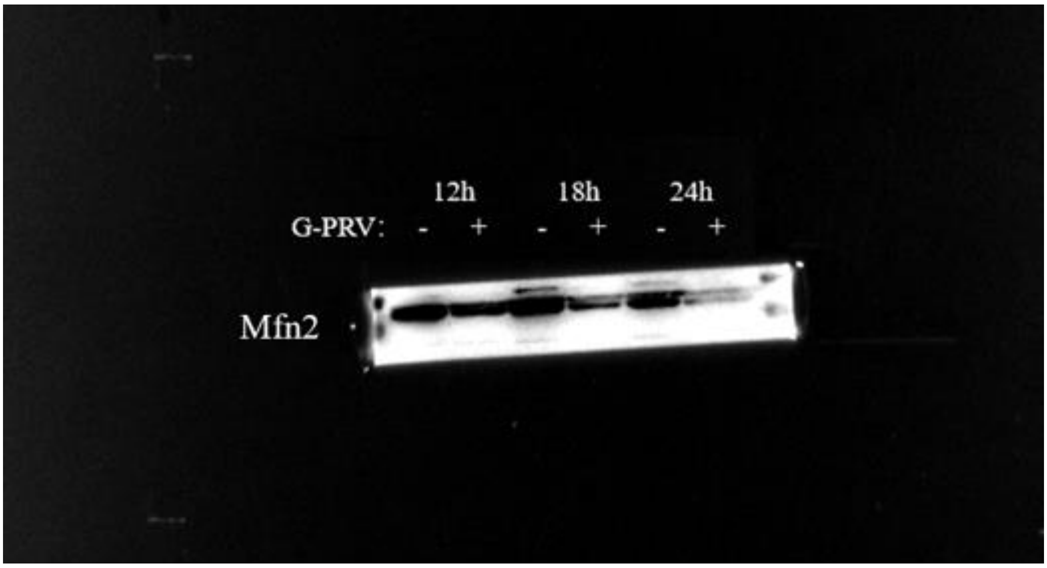

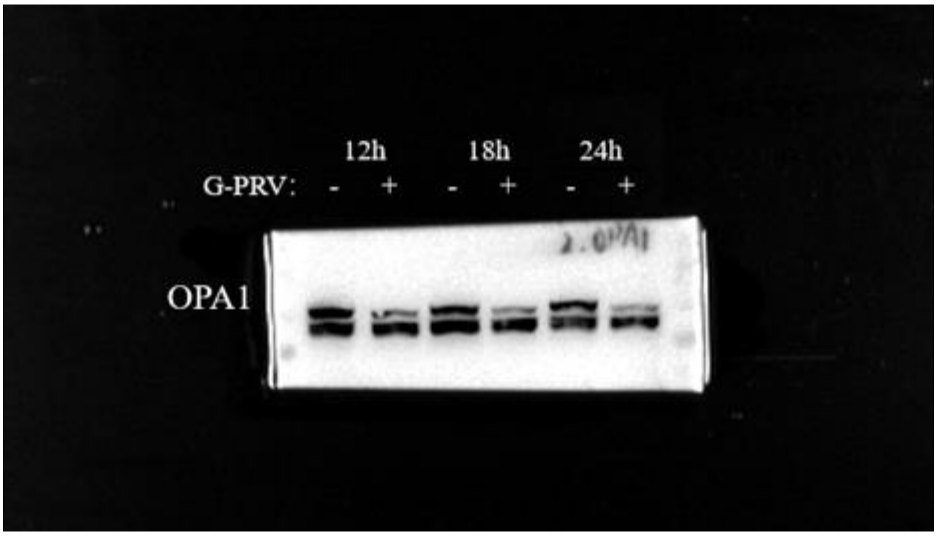

Figure S1.B(right)

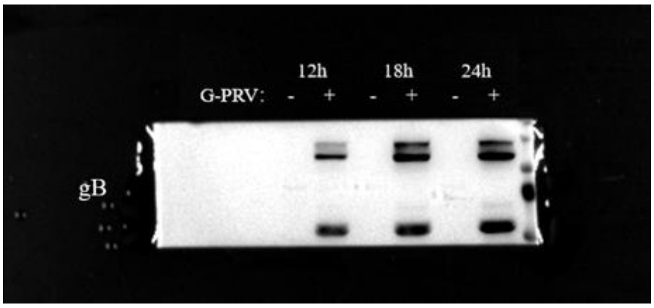

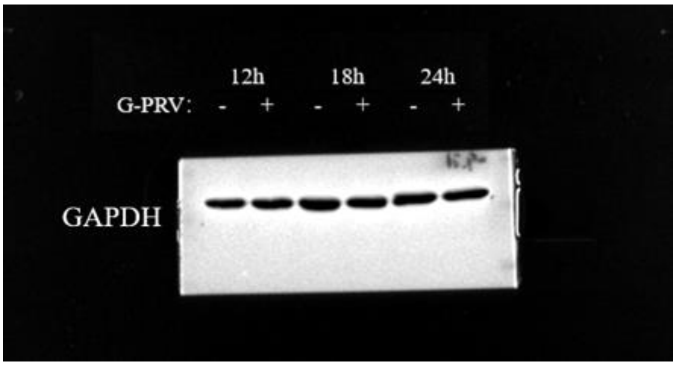

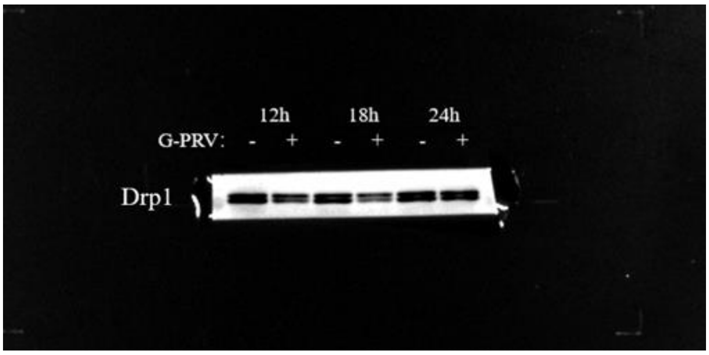

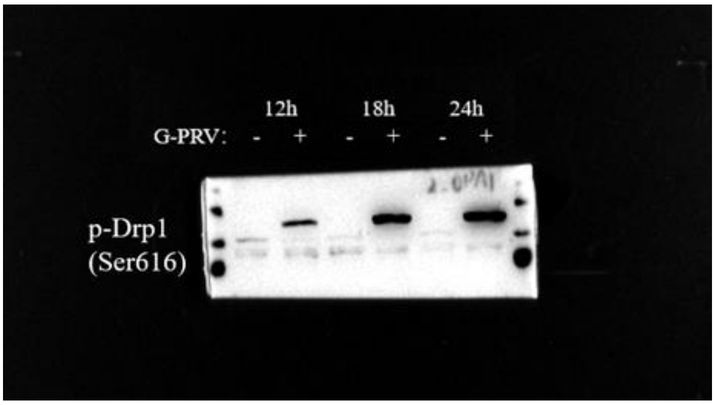

Figure S1.B(right) repeat 2

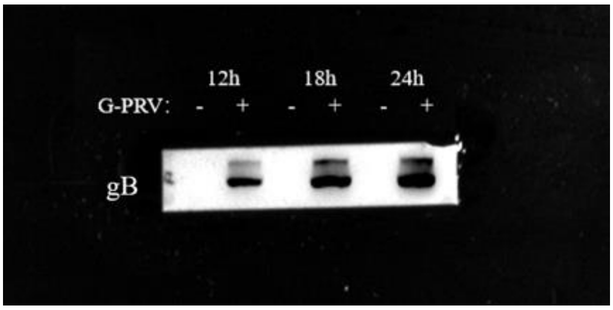

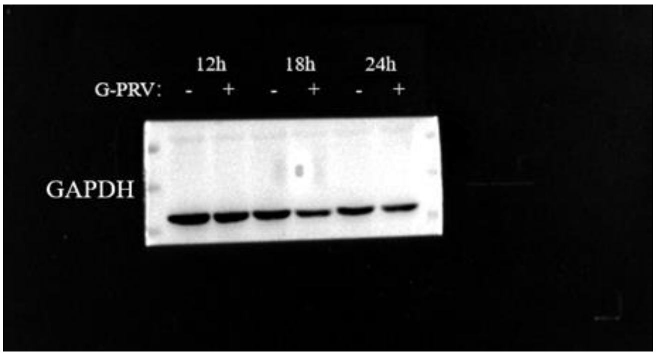

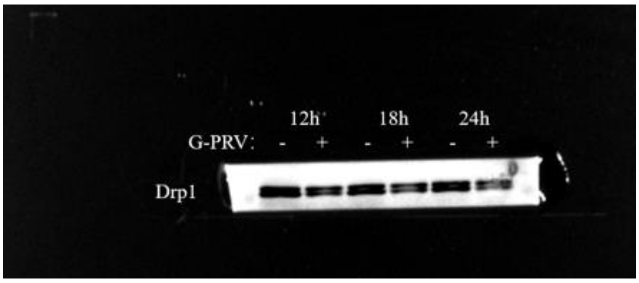

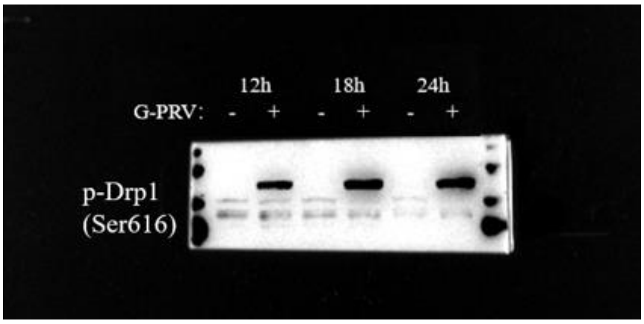

Figure S1.B(right) repeat 3

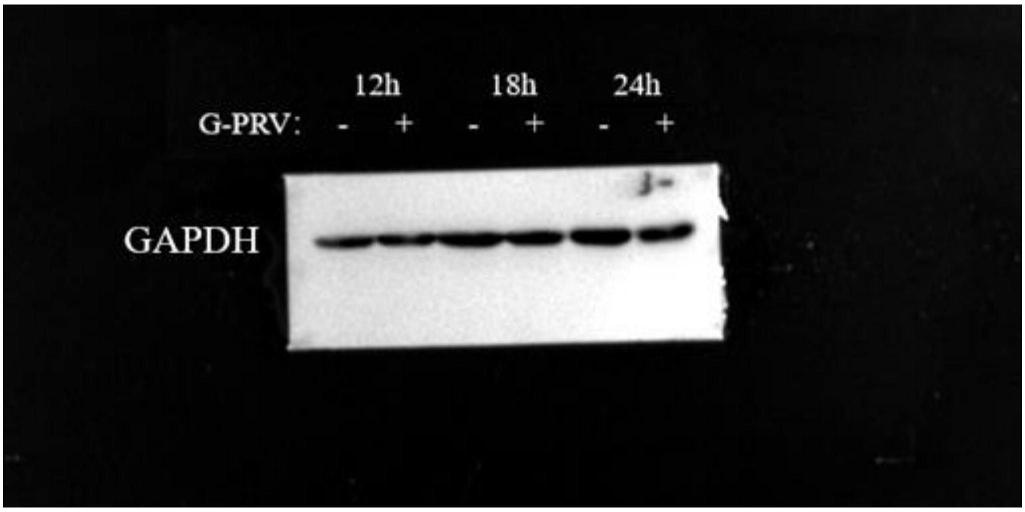

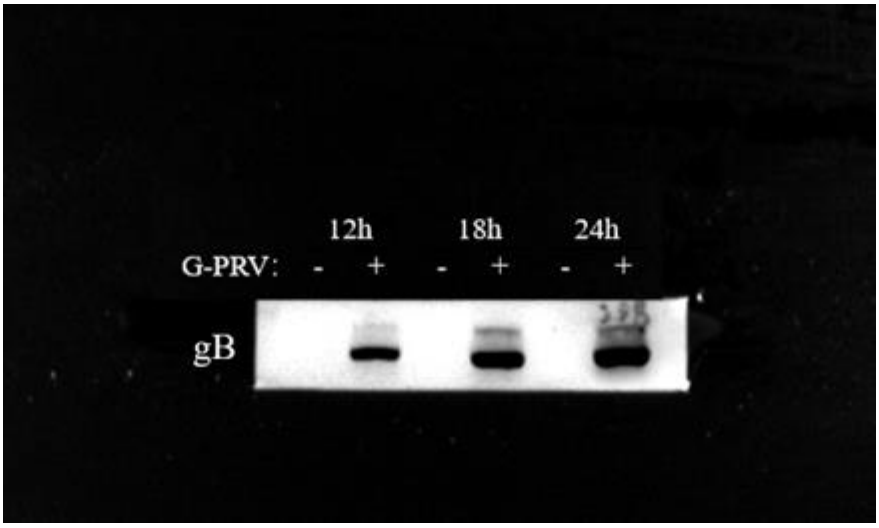
12h 18h 24h
G-PRV: - + - + - +
gB

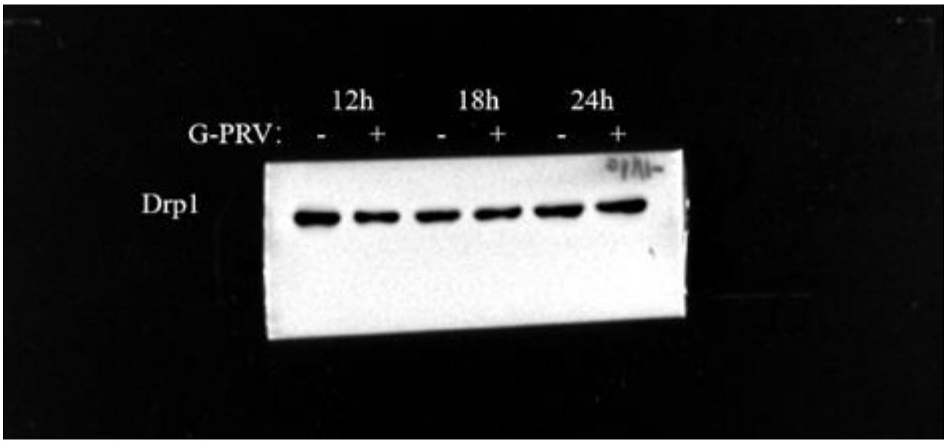
12h 18h 24h
G-PRV: - + - + - +
Drp1

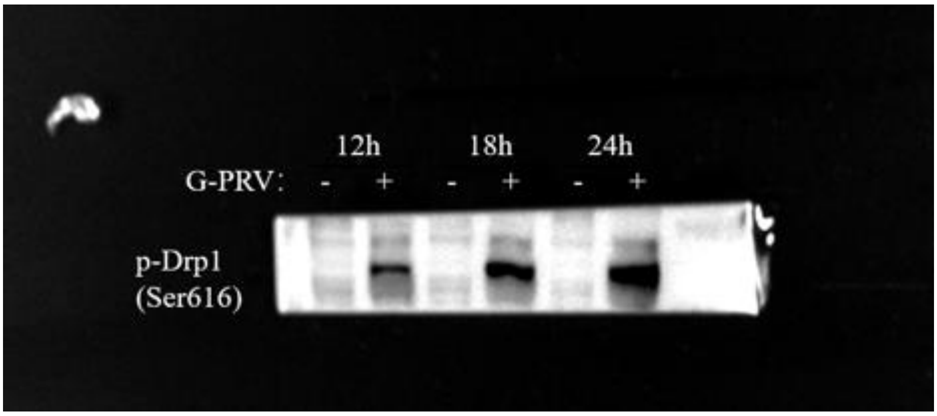
12h 18h 24h
G-PRV: - + - + - +
p-Drp1
(Ser616)

Figure S1. D

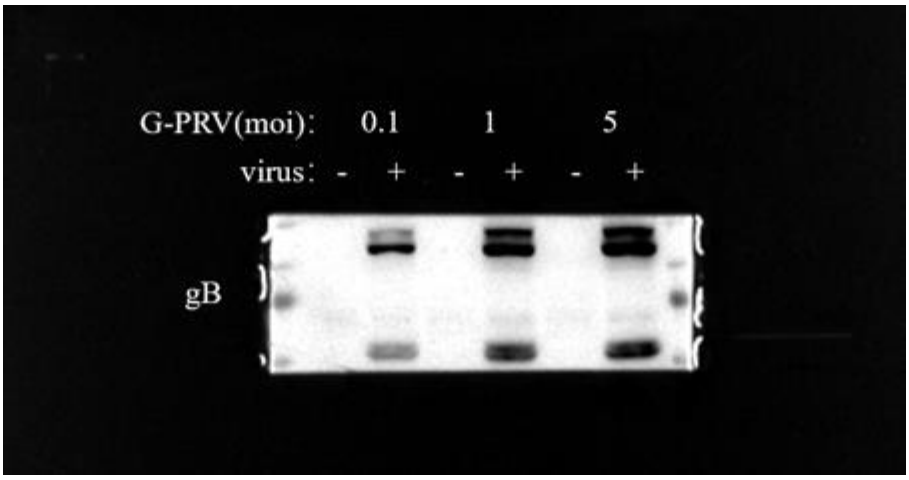
G-PRV(moi): 0.1 1 5
virus: - + - + - +
gB

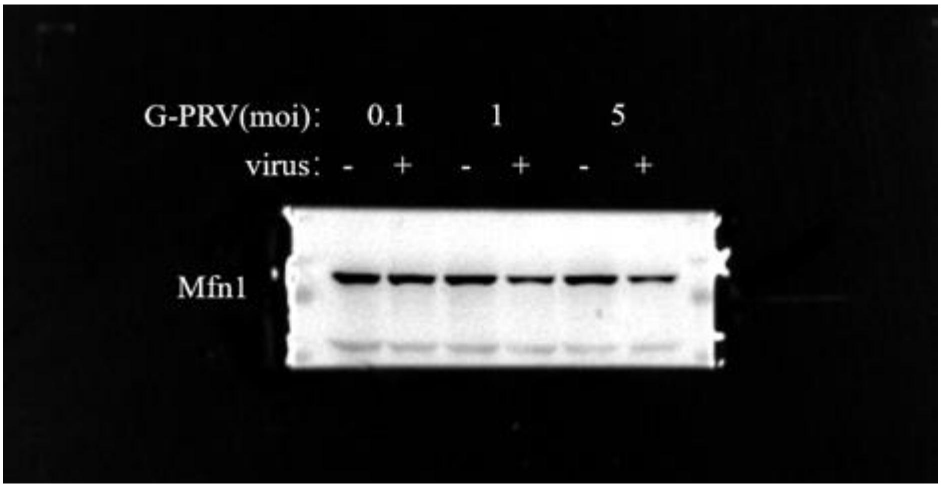
G-PRV(moi): 0.1 1 5
virus: - + - + - +
Mfn1

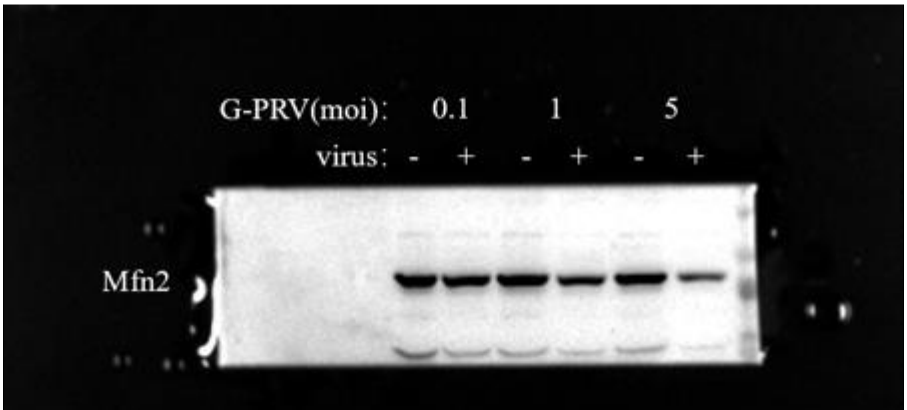
G-PRV(moi): 0.1 1 5
virus: - + - + - +
Mfn2

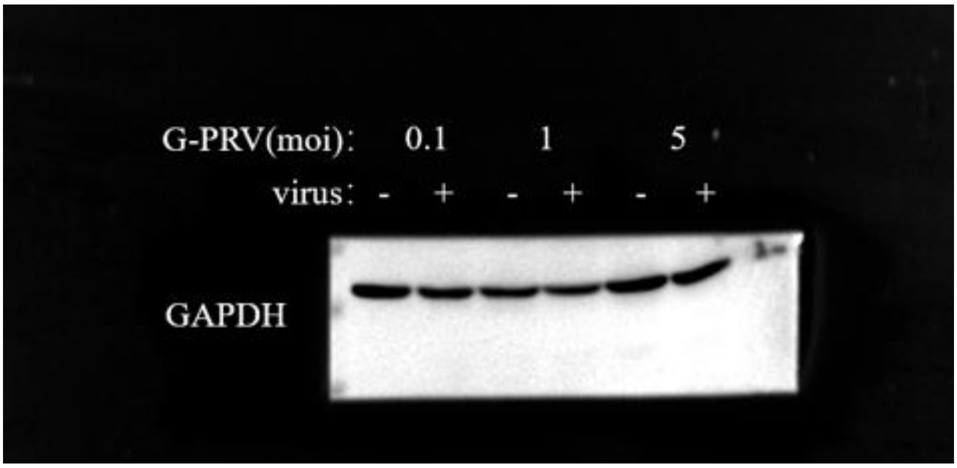

Figure S1E

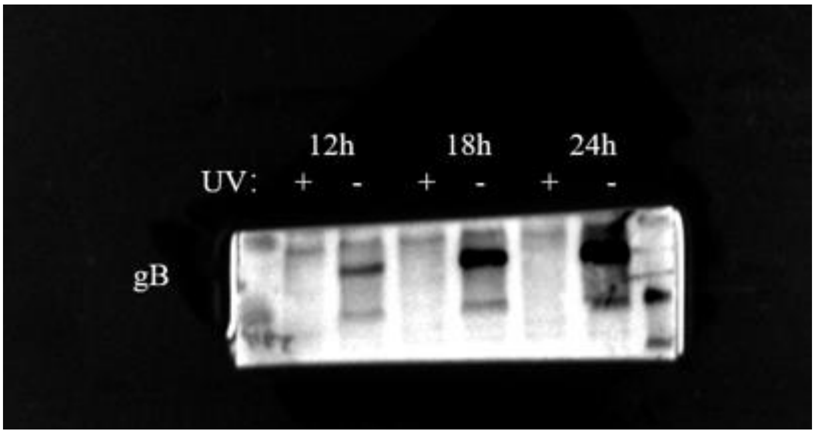

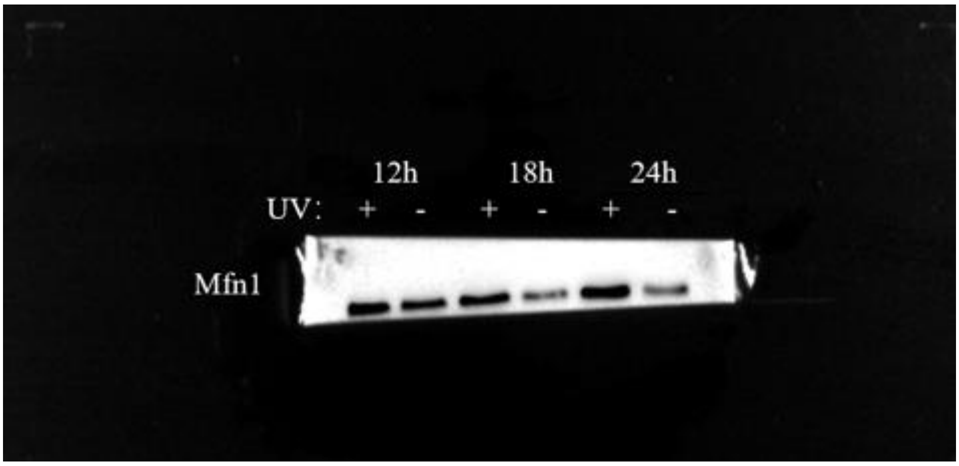

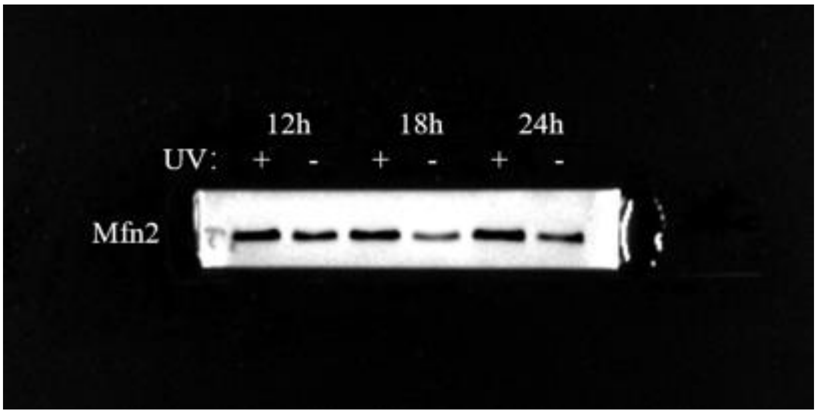

Figure S2A (up)

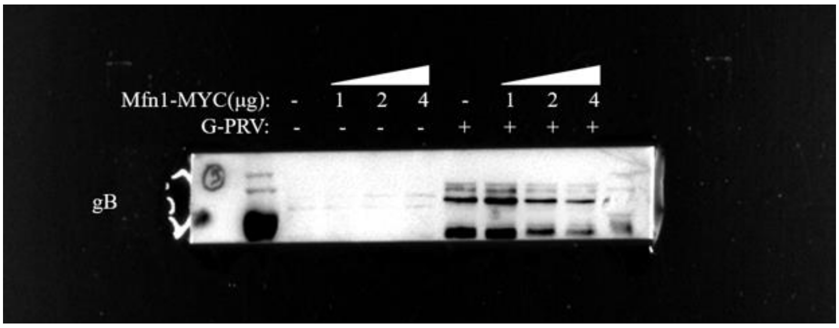

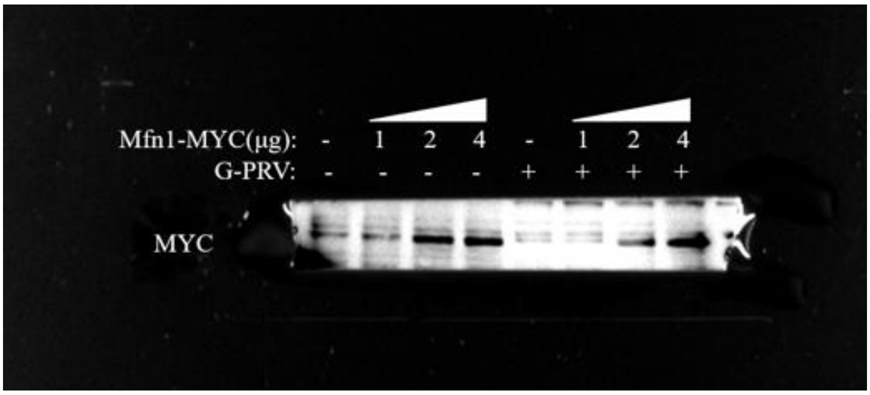

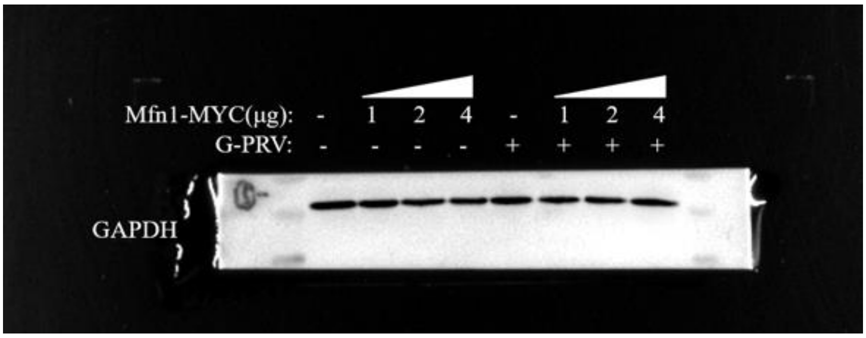

Figure S2A(up) repeat 2

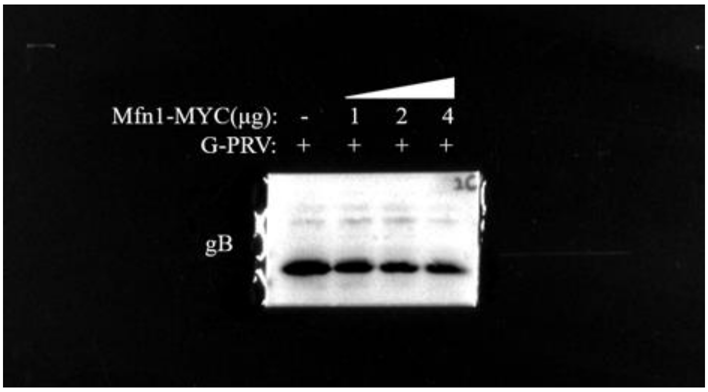

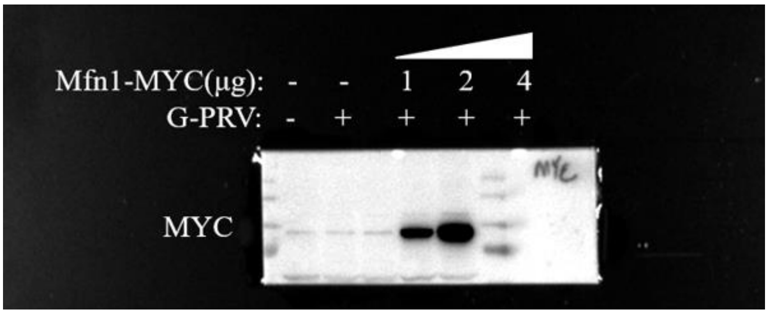

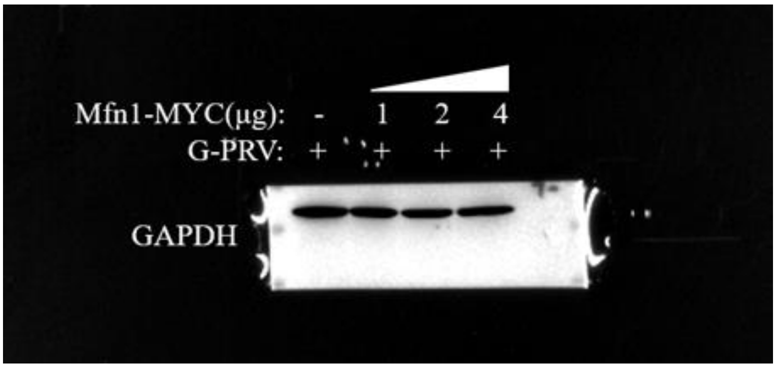

Figure S2A(up)repeat 3

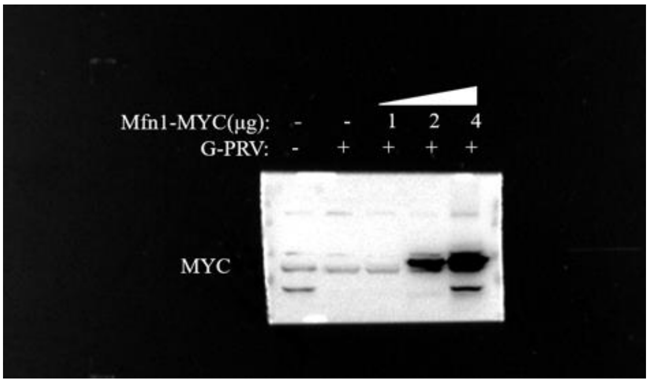

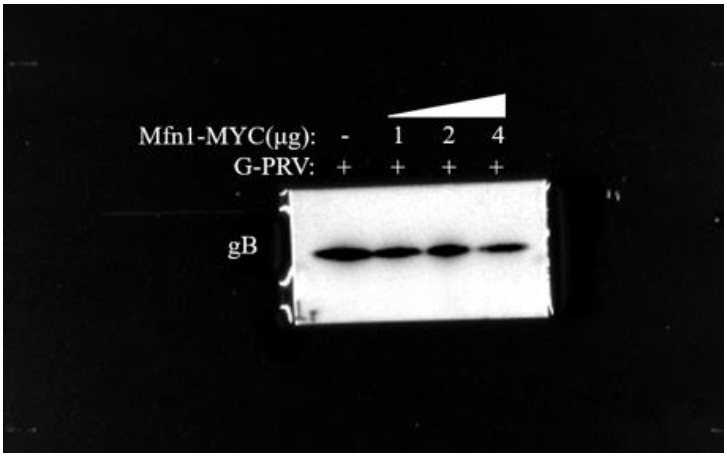

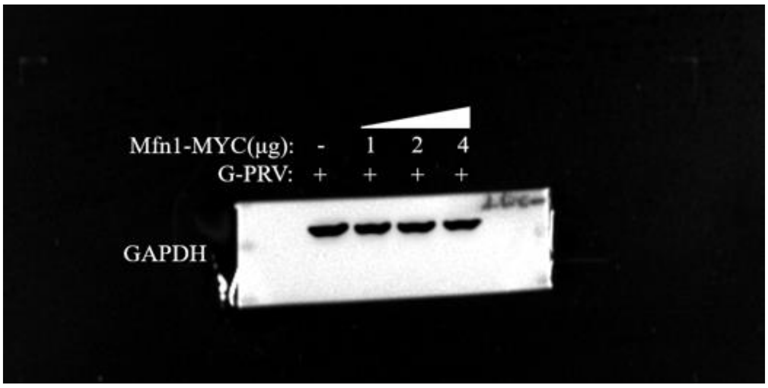

Figure S2A(down)

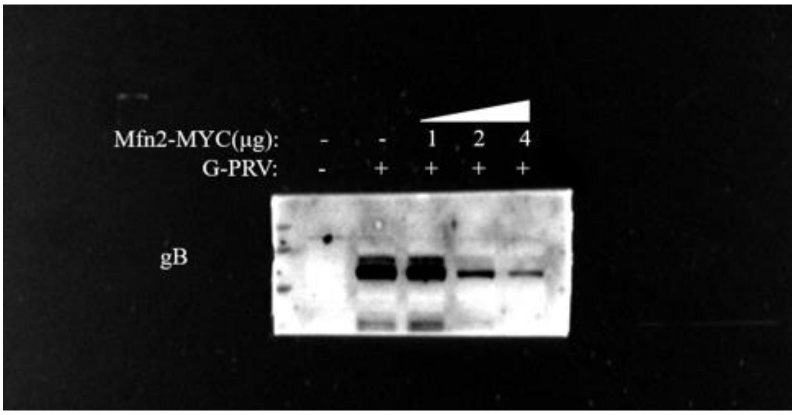

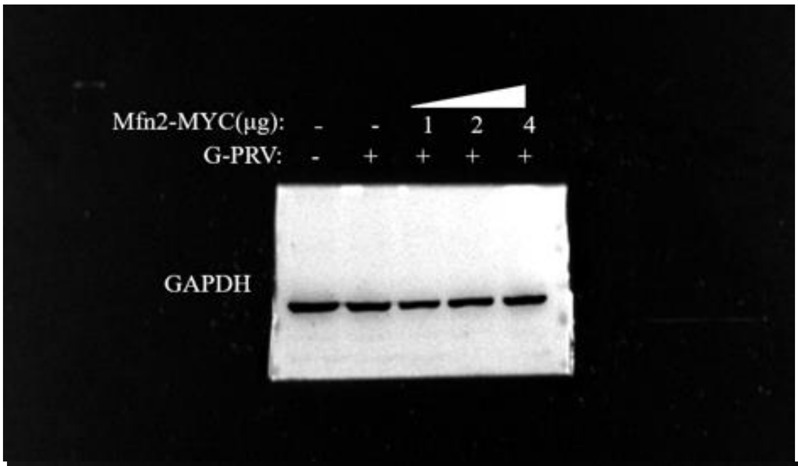

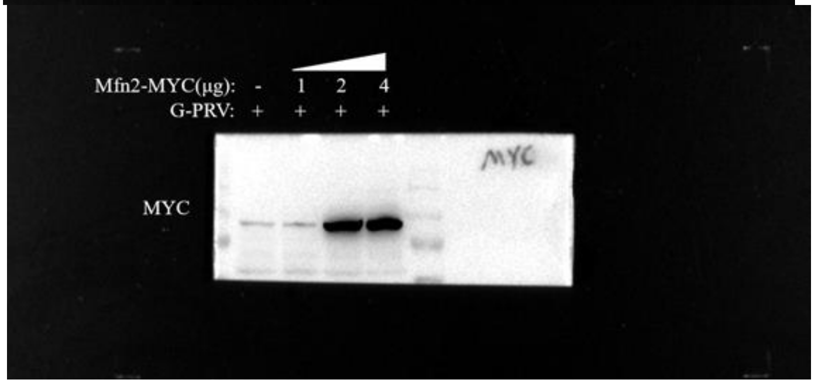

Figure S2A(down) repeat 2

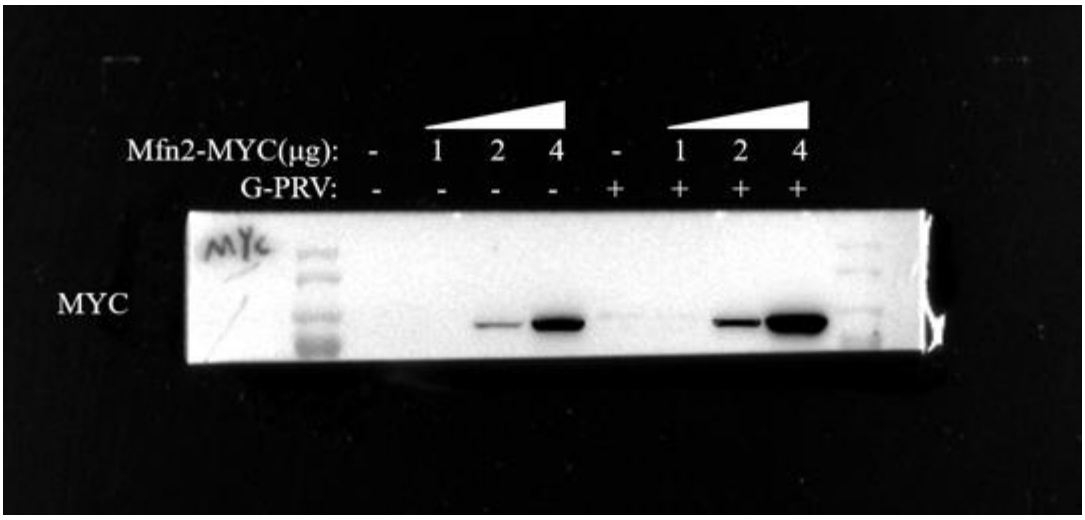

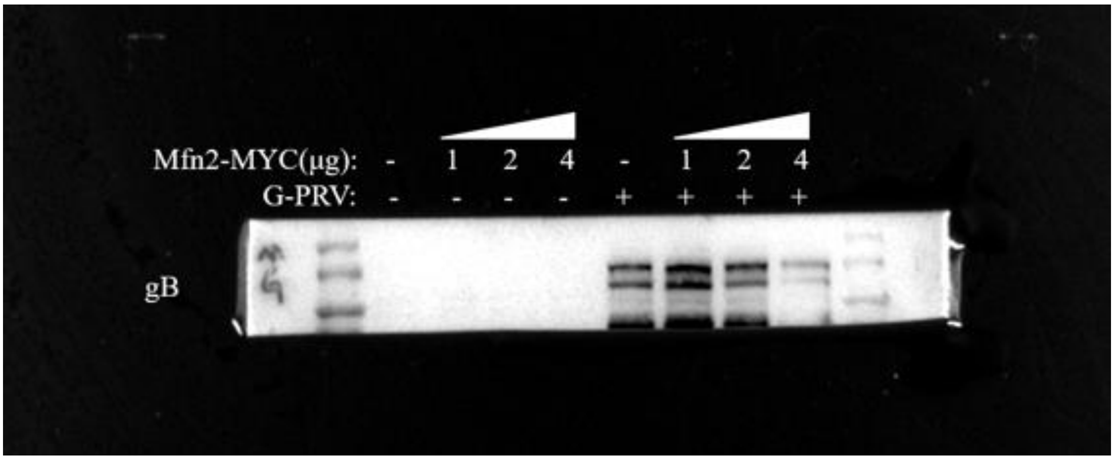

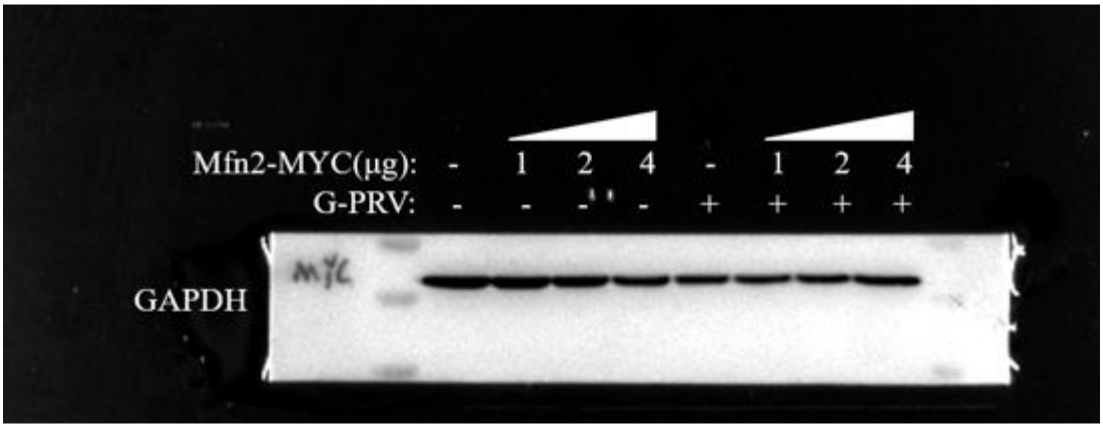

Figure S2A(down) repeat 3

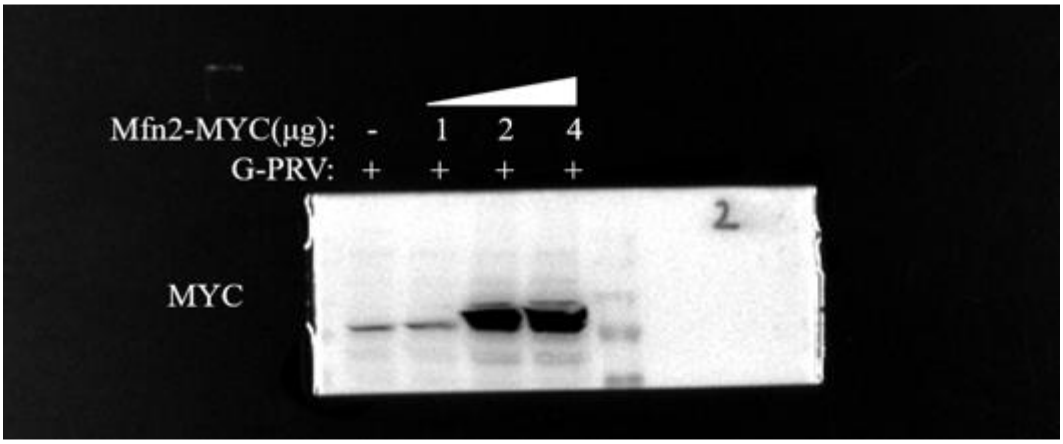

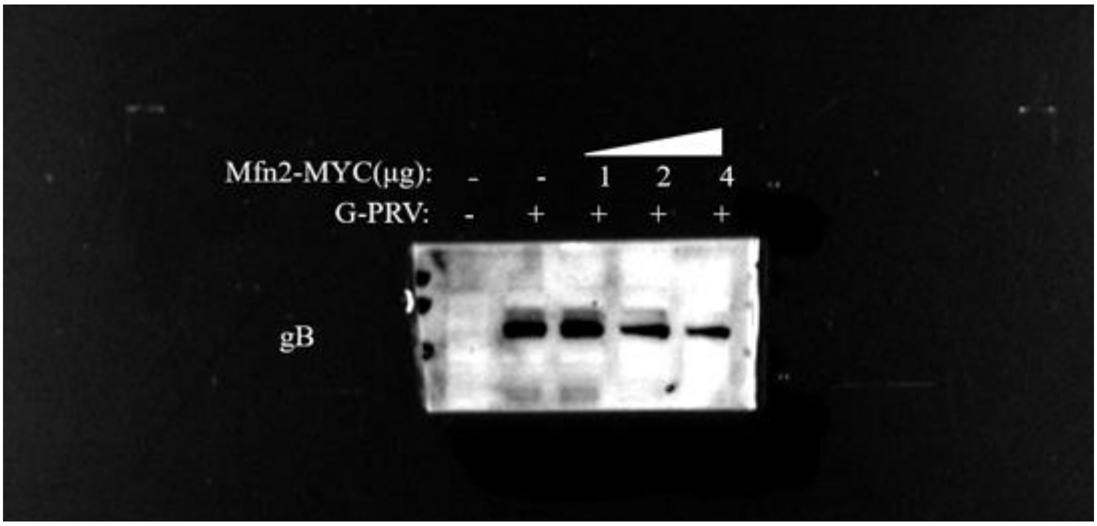

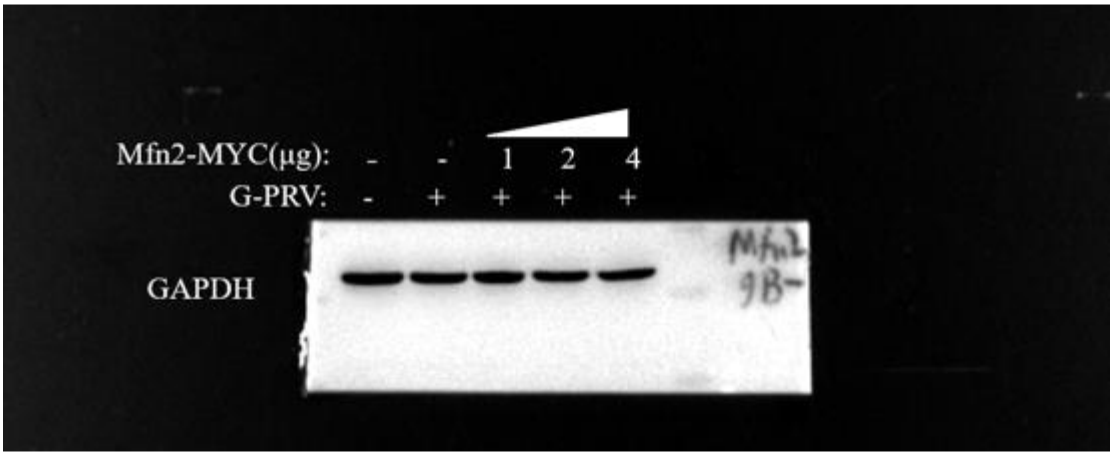

Figure S2B(up)

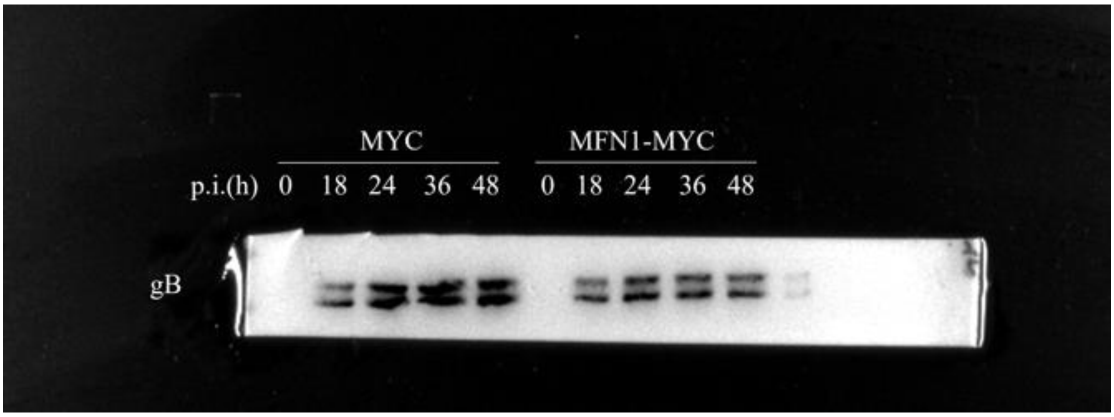

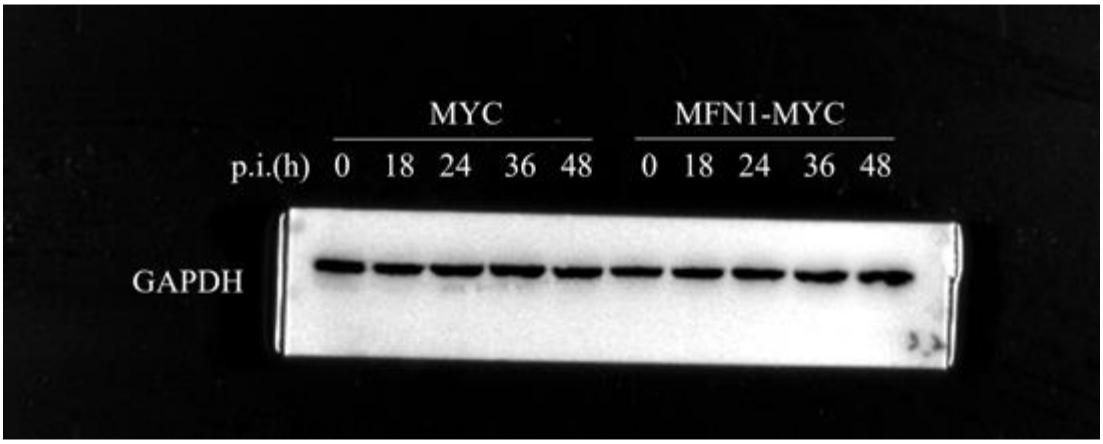

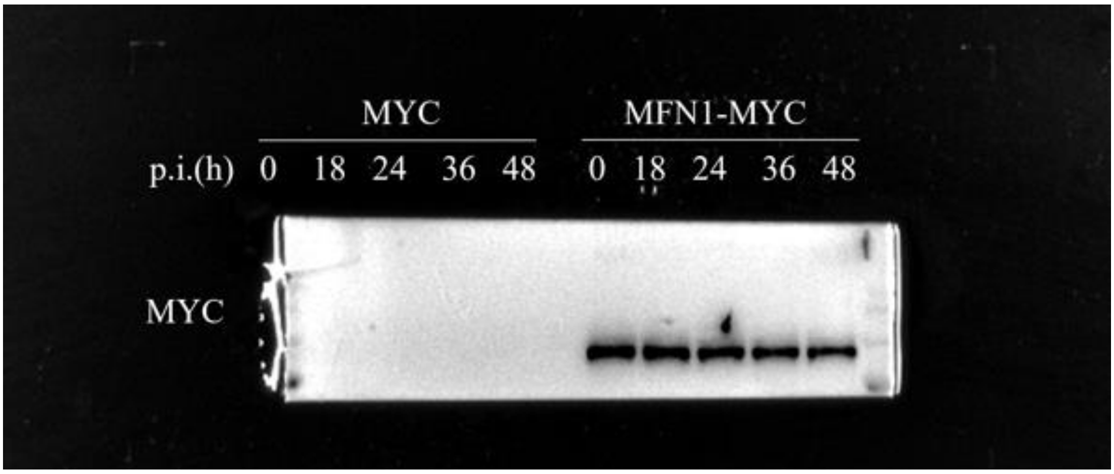

Figure S2B(down)

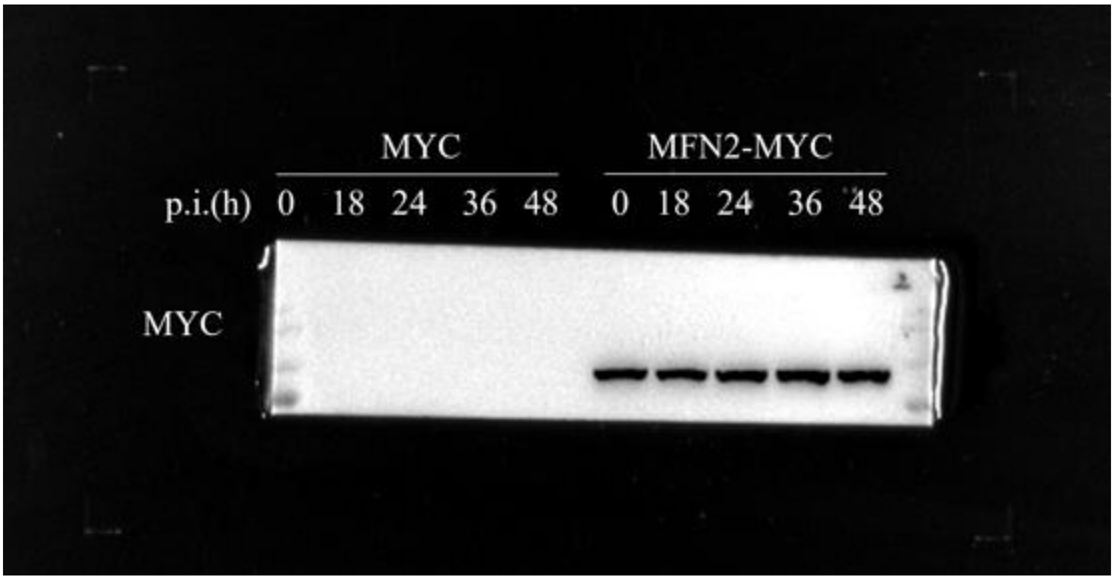

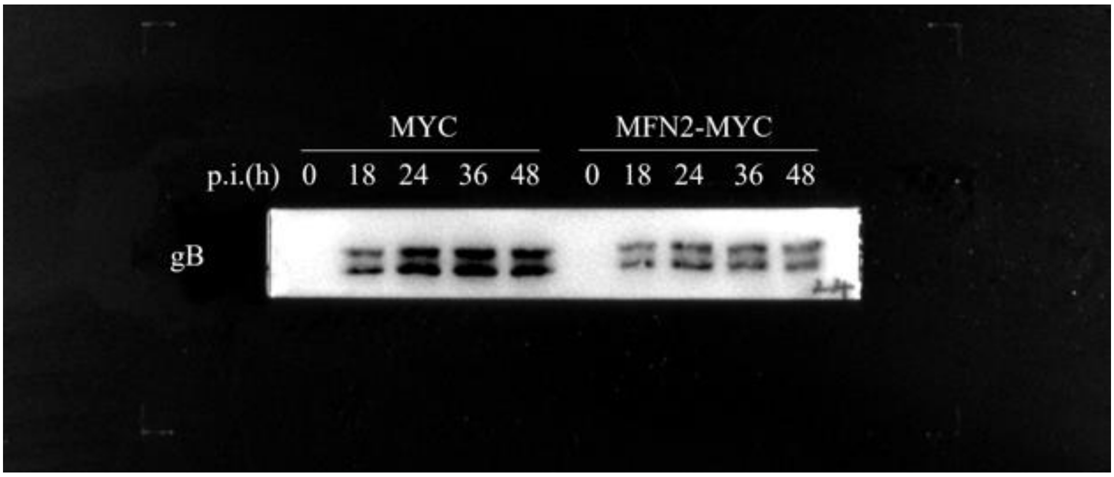
MYC
MFN2-MYC
p.i.(h) 0 18 24 36 48 0 18 24 36 48
gB

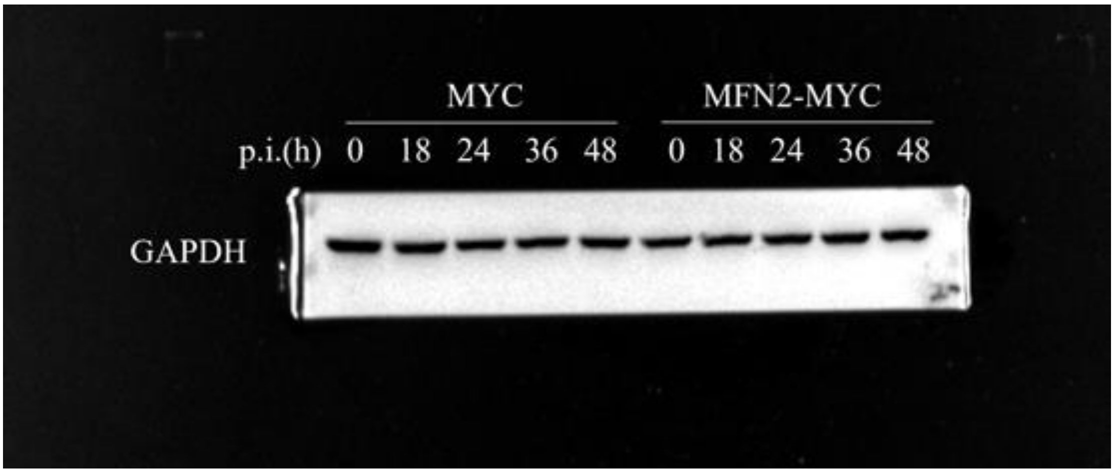
MYC
MFN2-MYC
p.i.(h) 0 18 24 36 48 0 18 24 36 48
GAPDH

Figure S2H

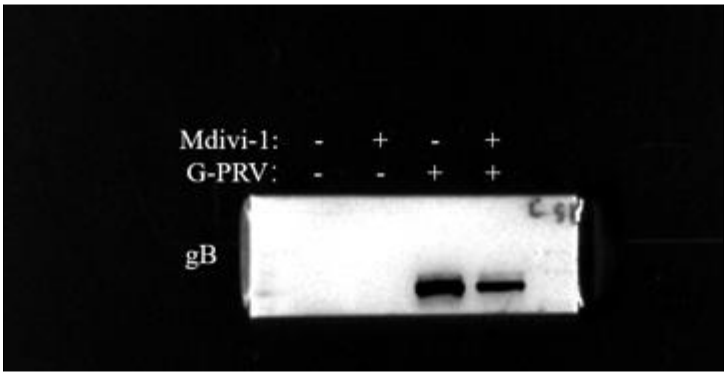
Mdivi-1: - + - +
G-PRV: - - + +
gB

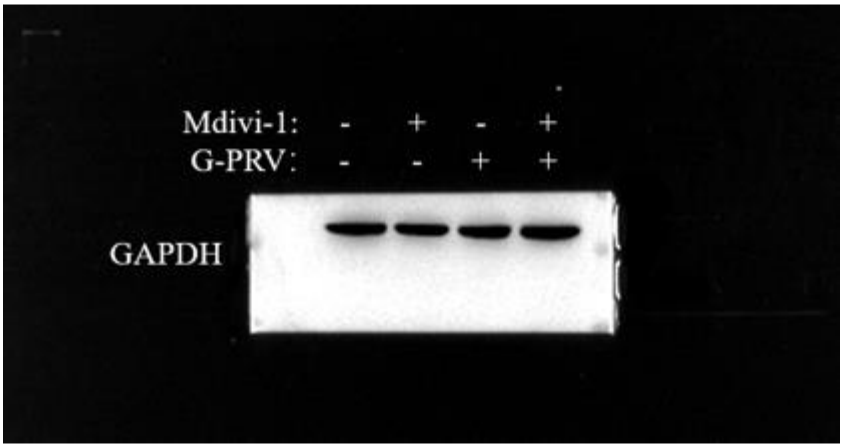

Figure S2H repeat 2

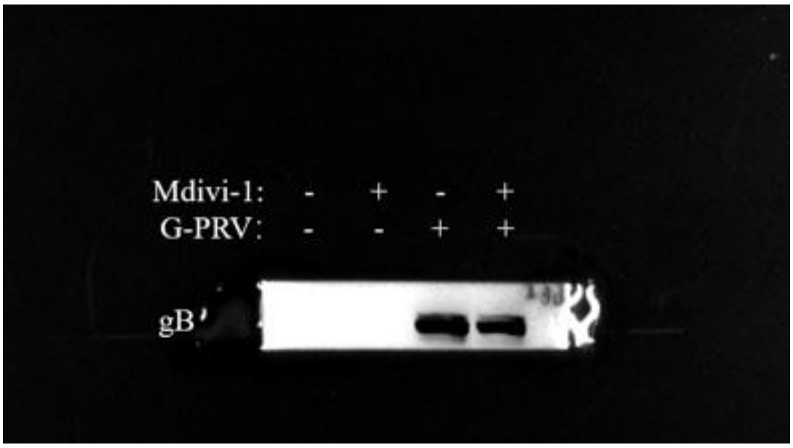

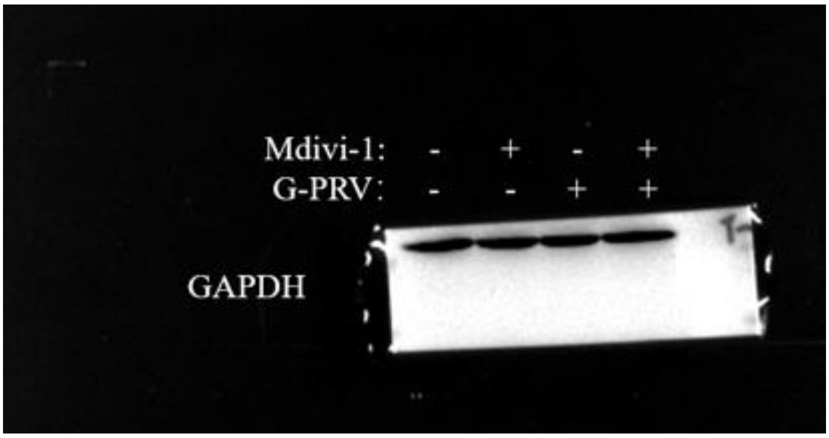

Figure S2H repeat 3

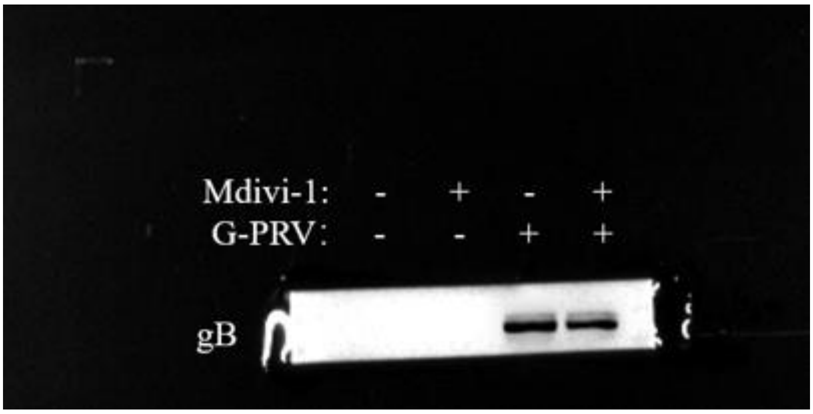

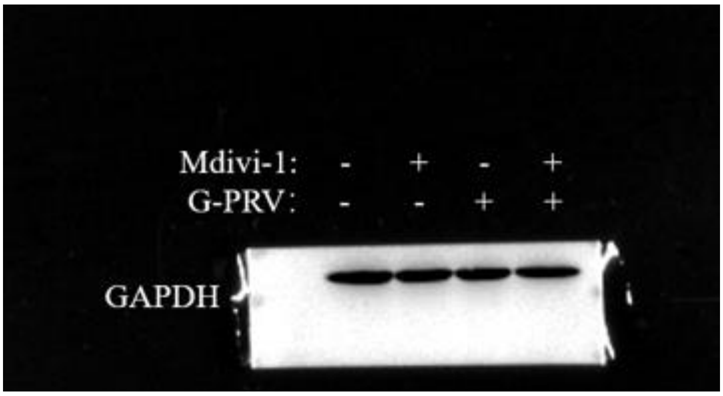

Figure S3A (left)

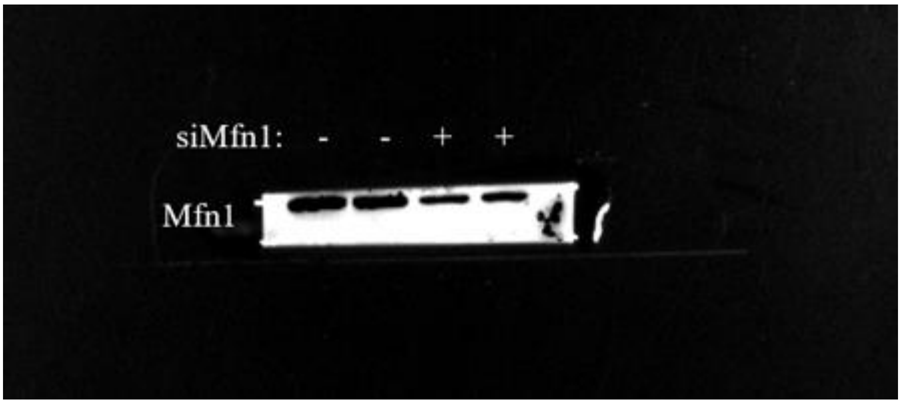

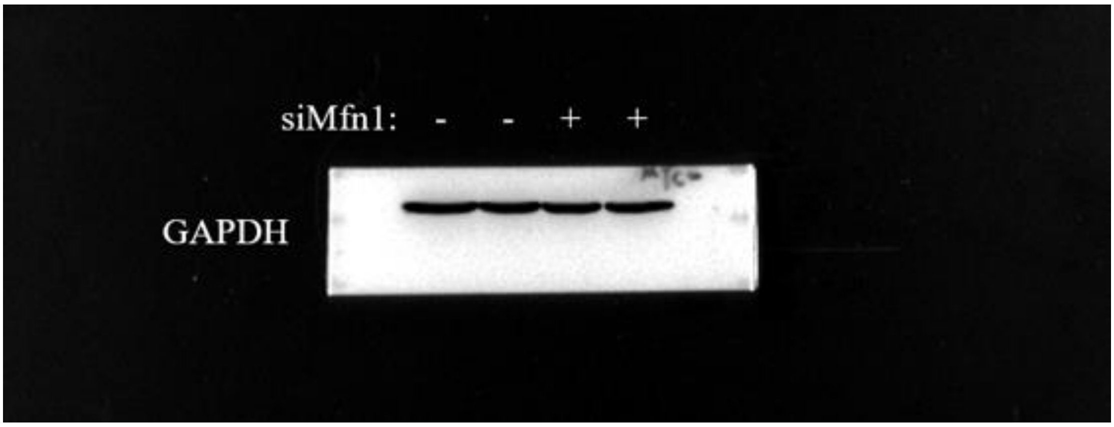

Figure S3A (middle)

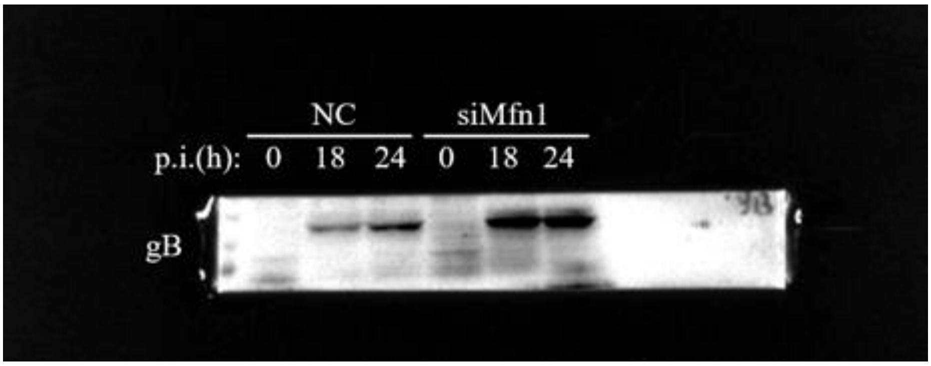

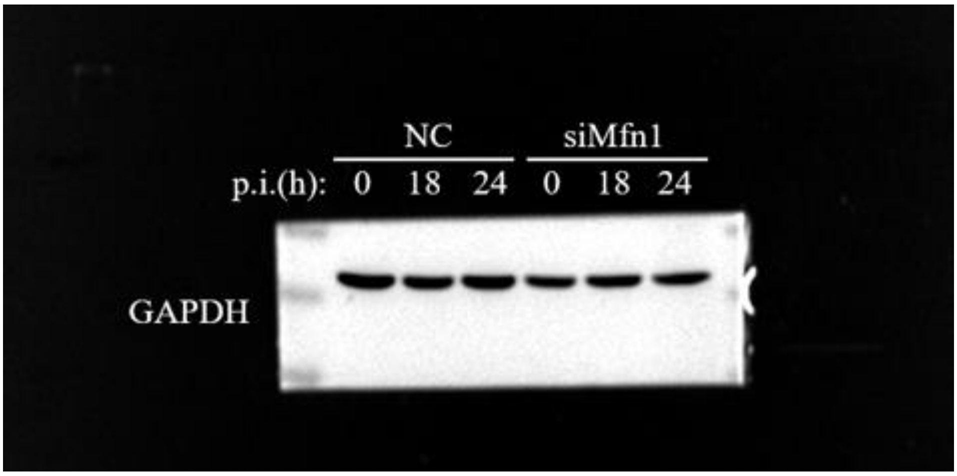

Figure S3A (middle) repeat 2

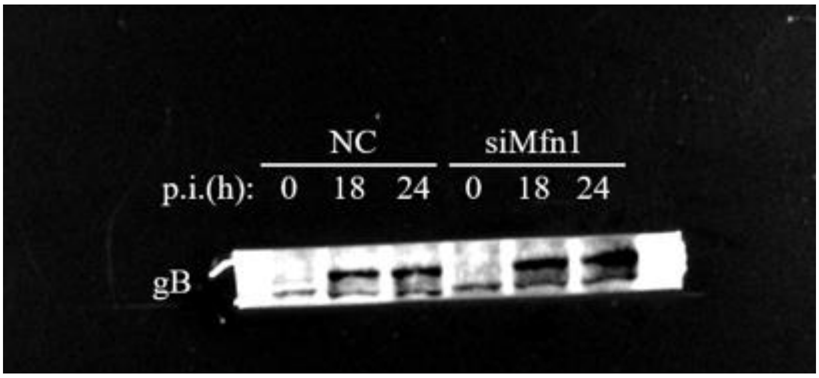

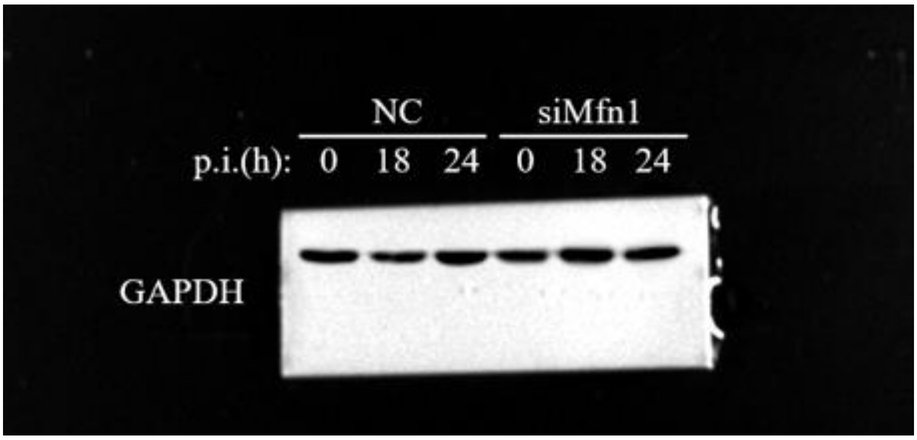

Figure S3A (middle) repeat 3

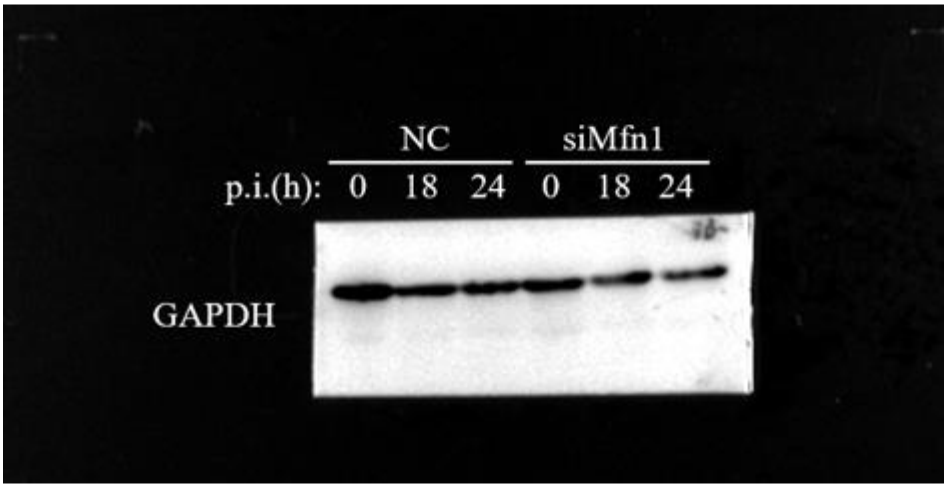

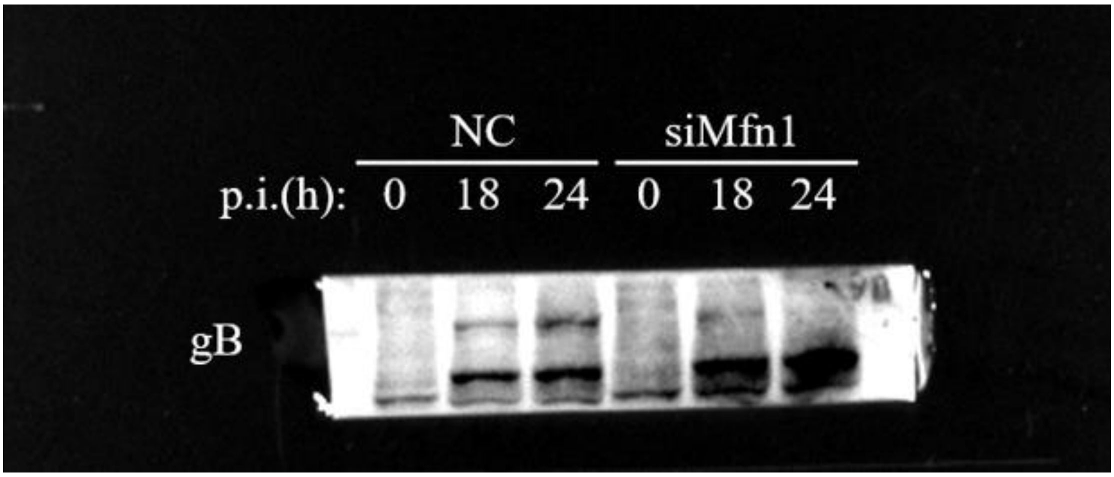

Figure S3B (left)

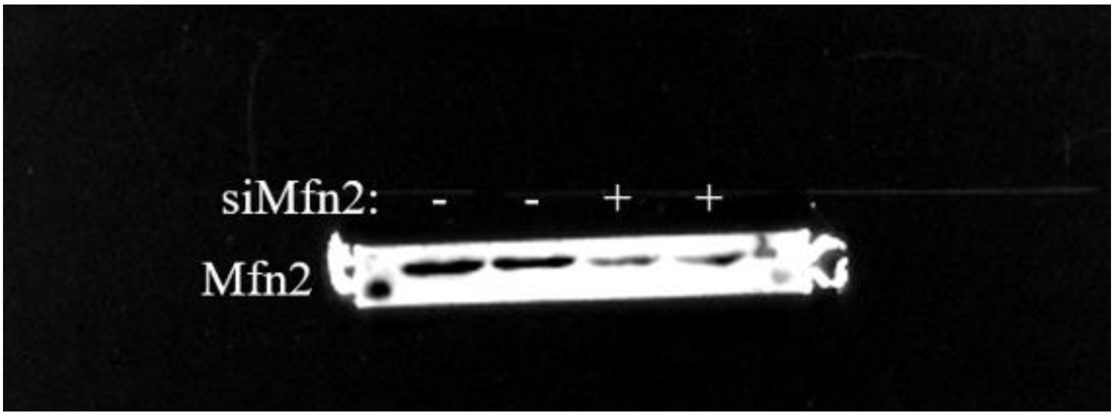

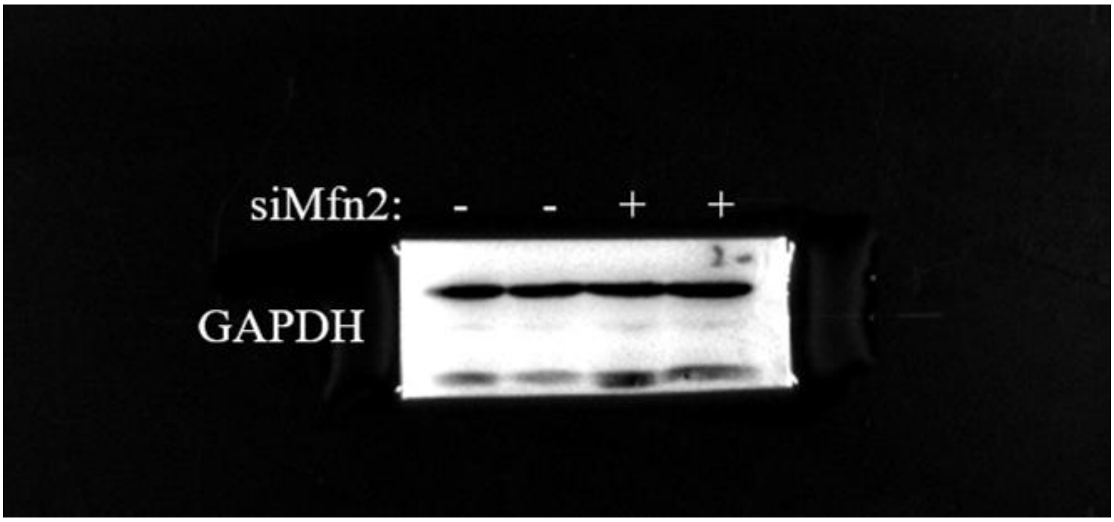

Figure S3B (middle)

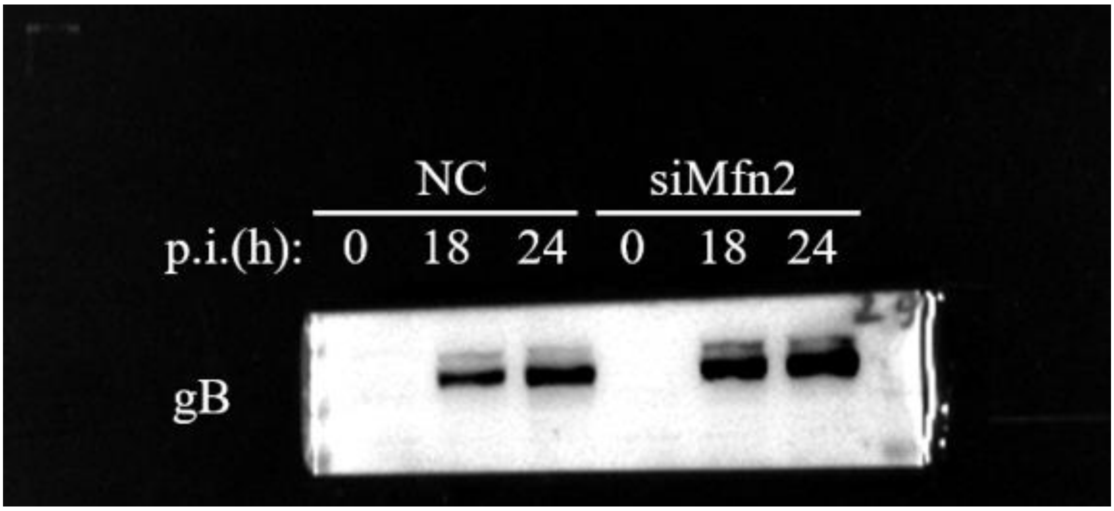

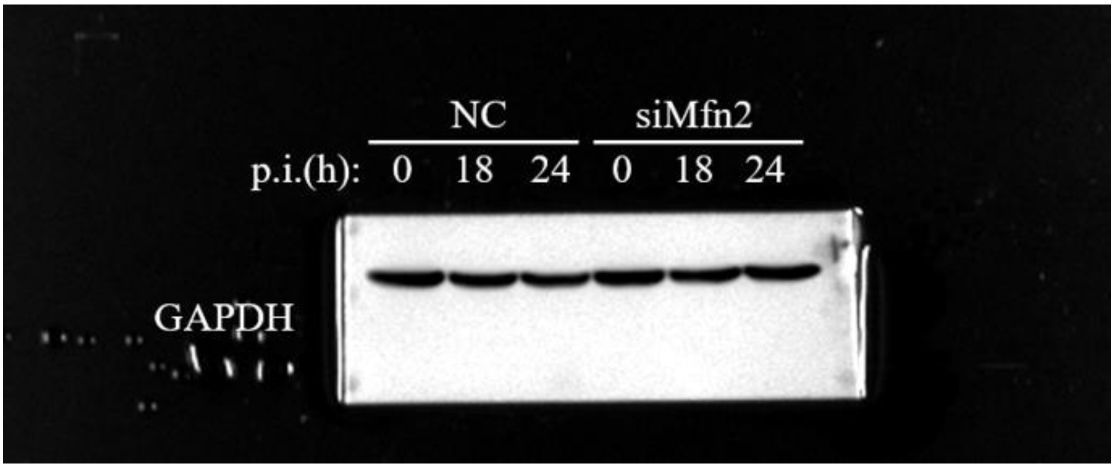

Figure S3B (middle) repeat 2

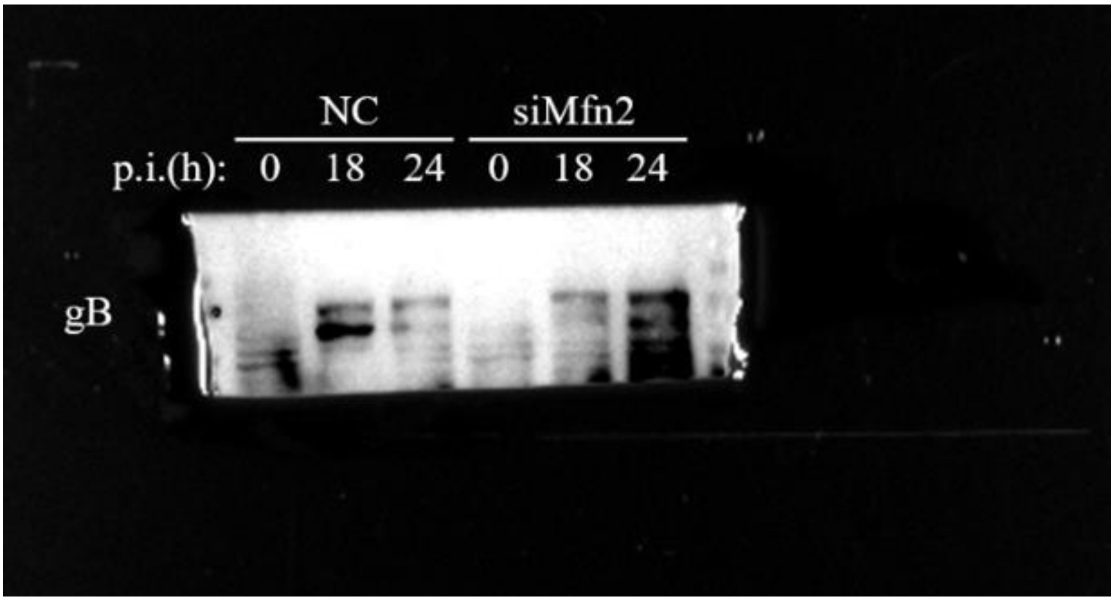

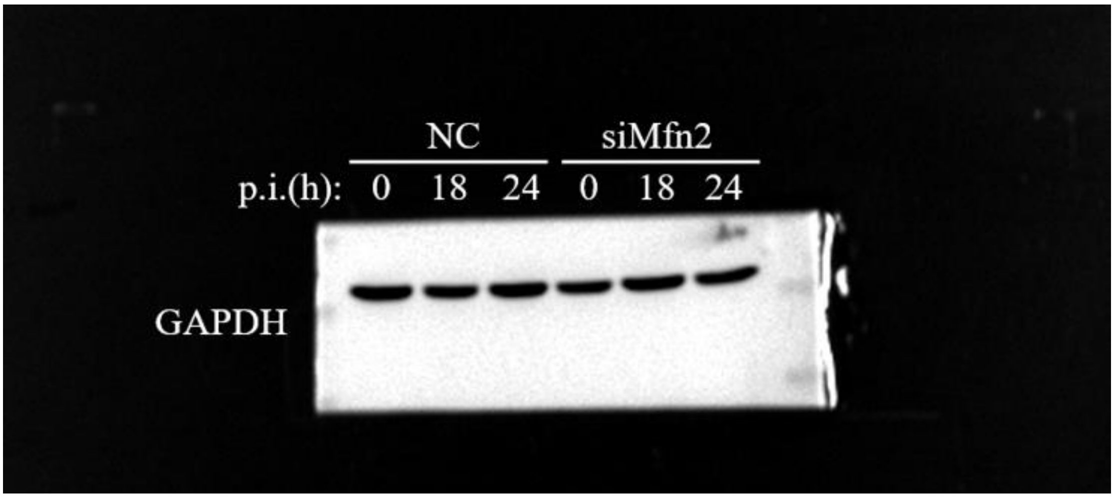

Figure S3B (middle) repeat 3

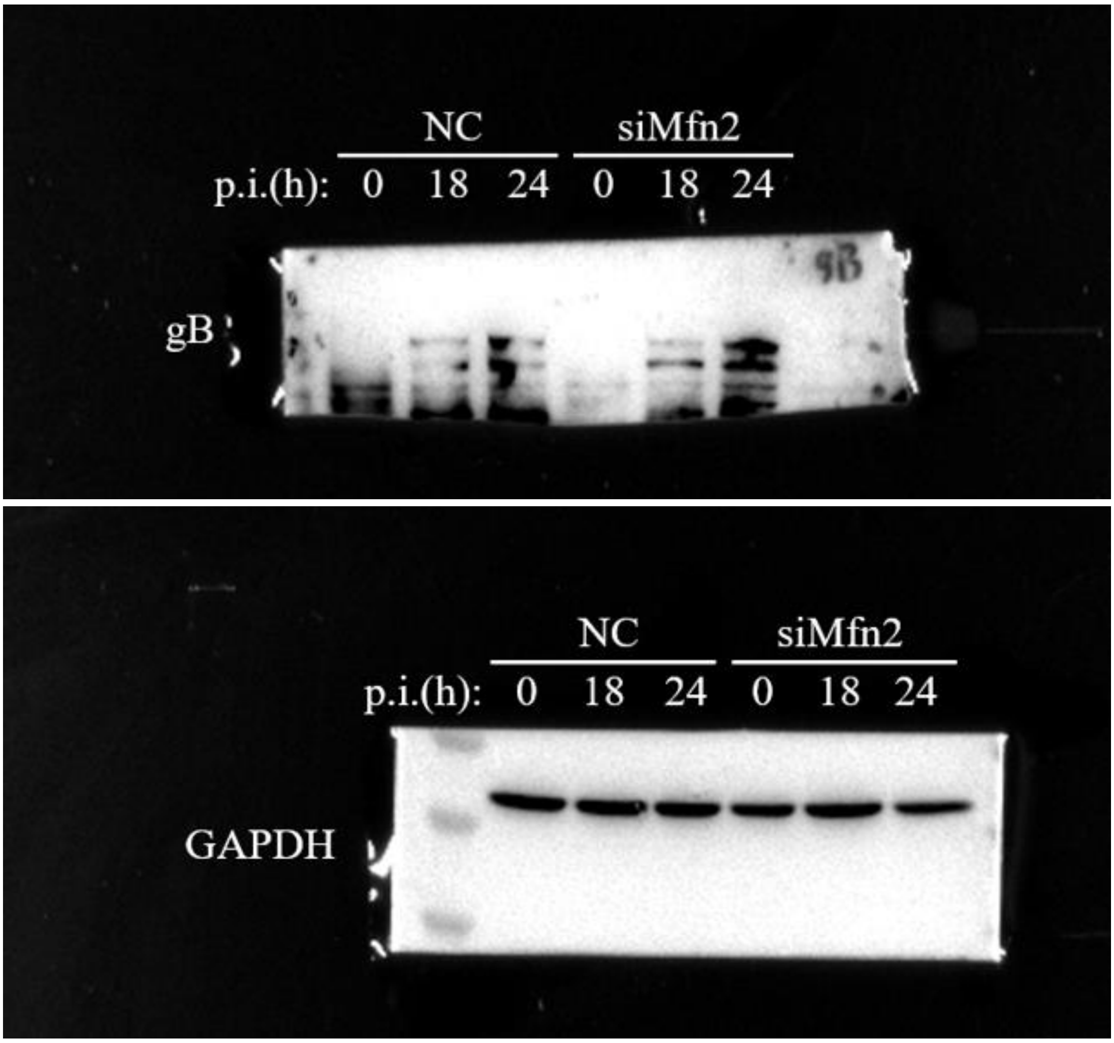

Figure S4C

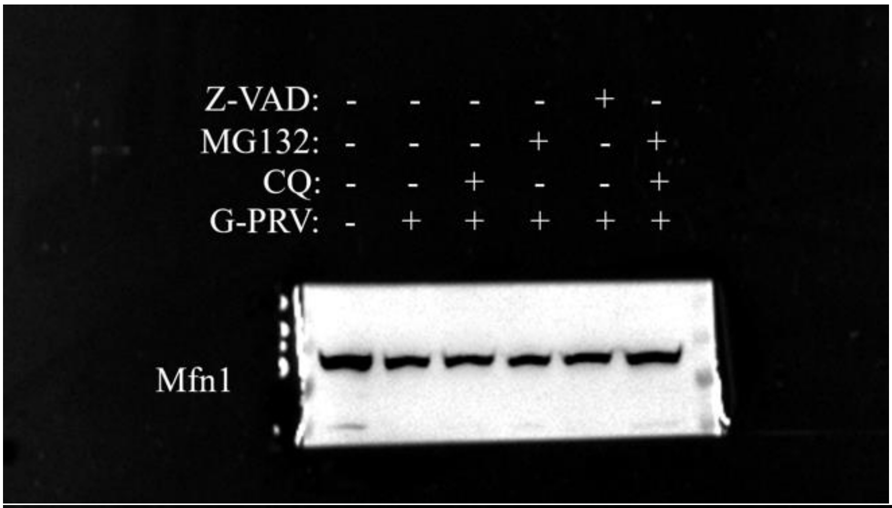
Z-VAD: - - - - + -
MG132: - - - + - +
CQ: - - + - - +
G-PRV: - + + + + +
Mfn1

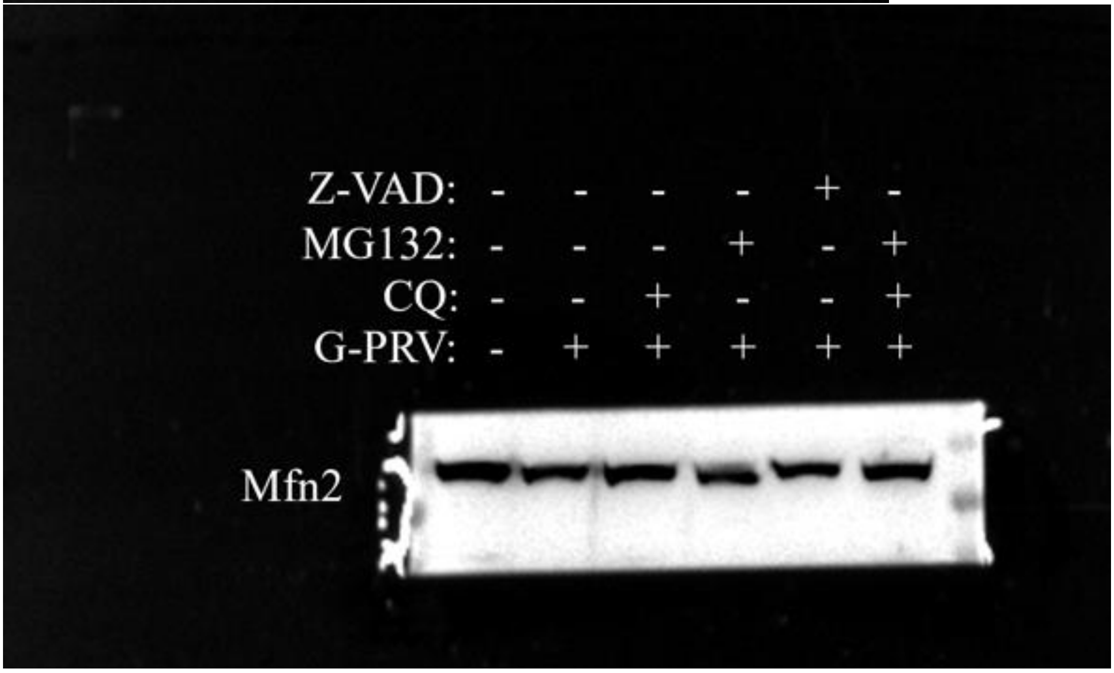
Z-VAD: - - - - + -
MG132: - - - + - +
CQ: - - + - - +
G-PRV: - + + + + +
Mfn2

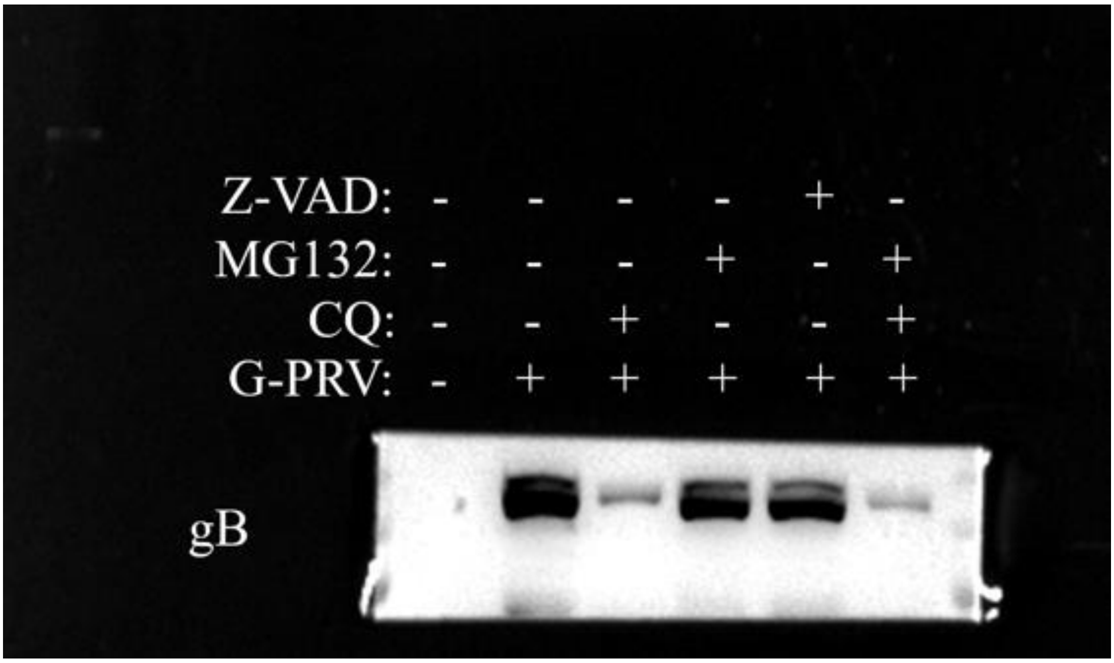
Z-VAD: - - - - + -
MG132: - - - + - +
CQ: - - + - - +
G-PRV: - + + + + +
gB

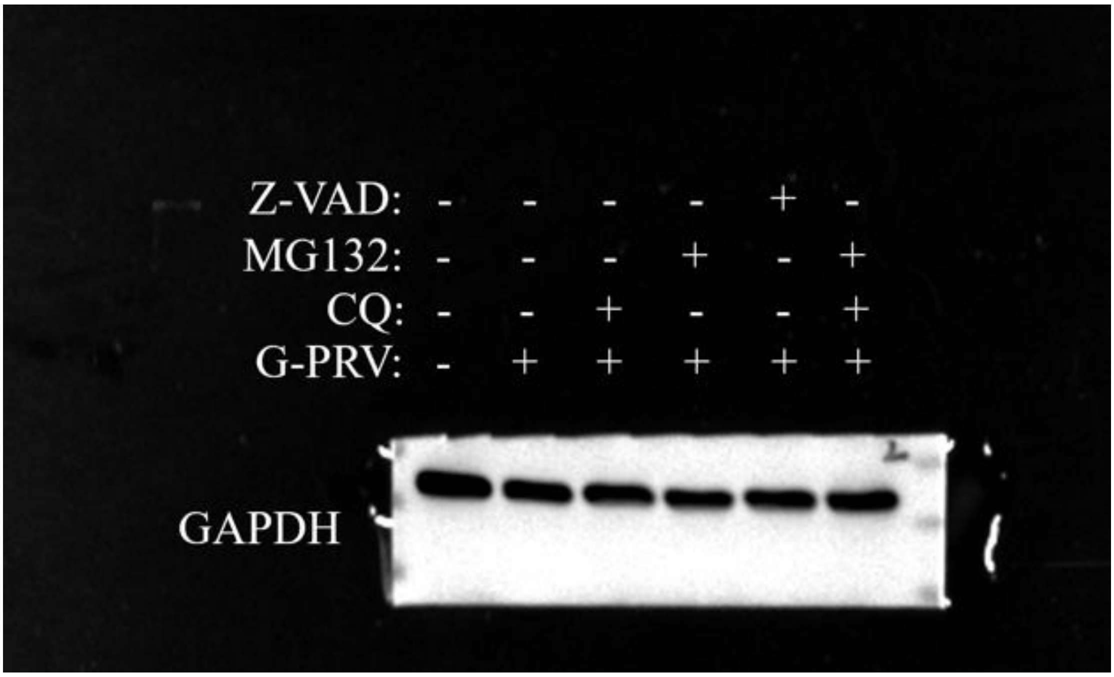

Figure S4C repeat 2

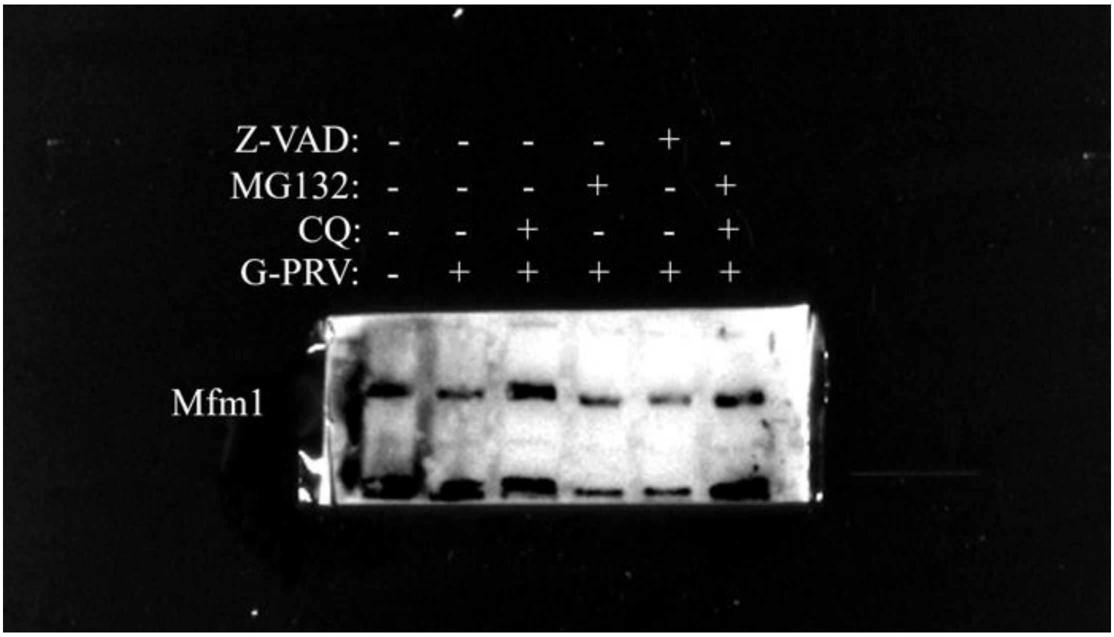

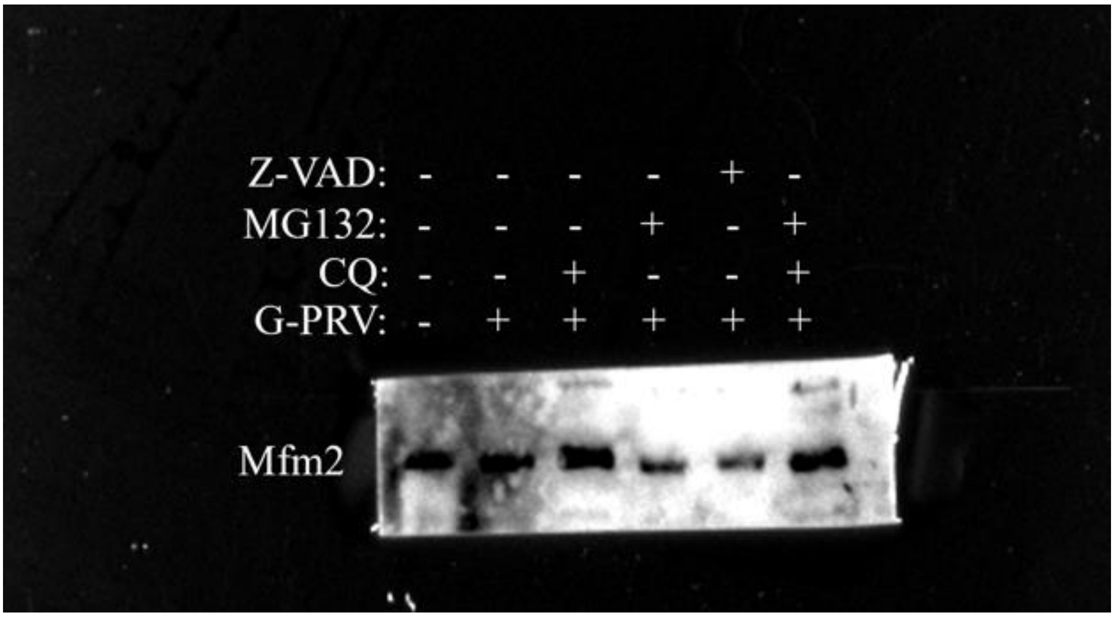
Z-VAD: - - - - + -
MG132: - - - + - +
CQ: - - + - - +
G-PRV: - + + + + +
Mfm2

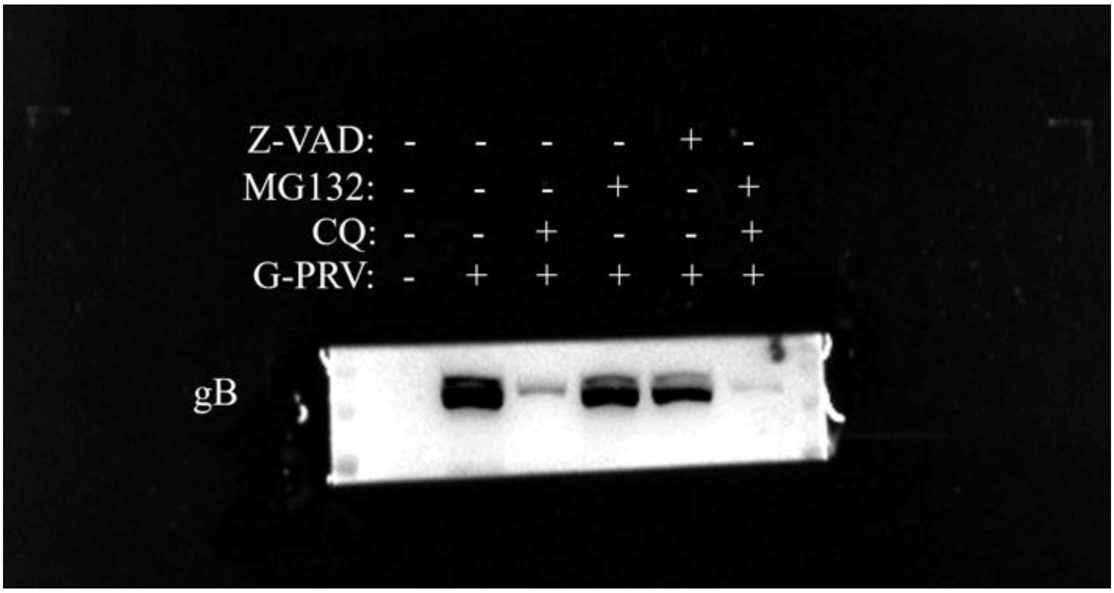
Z-VAD: - - - - + -
MG132: - - - + - +
CQ: - - + - - +
G-PRV: - + + + + +
gB

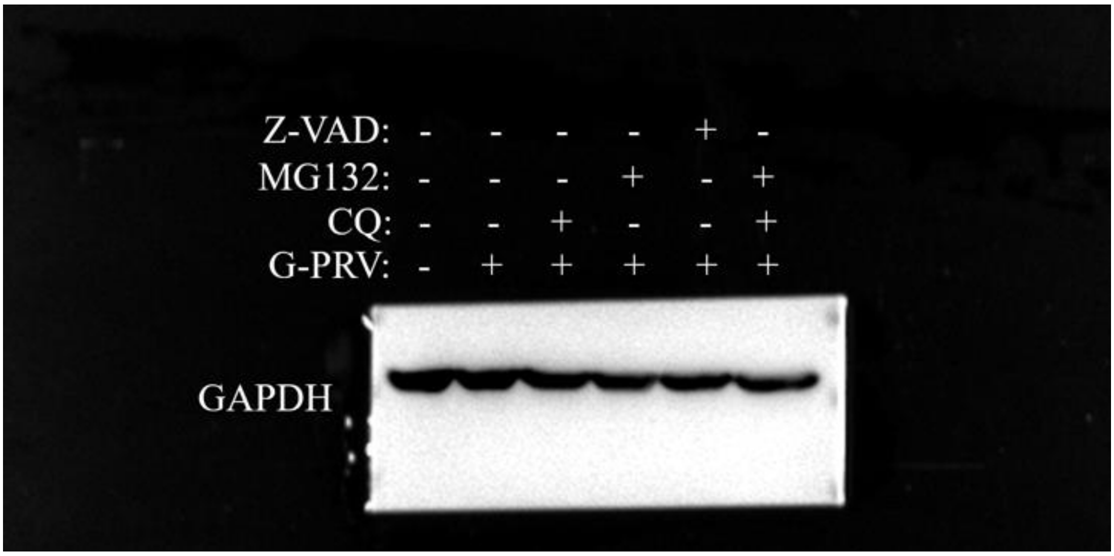
Z-VAD: - - - - + -
MG132: - - - + - +
CQ: - - + - - +
G-PRV: - + + + + +
GAPDH

Figure S4C repeat 3

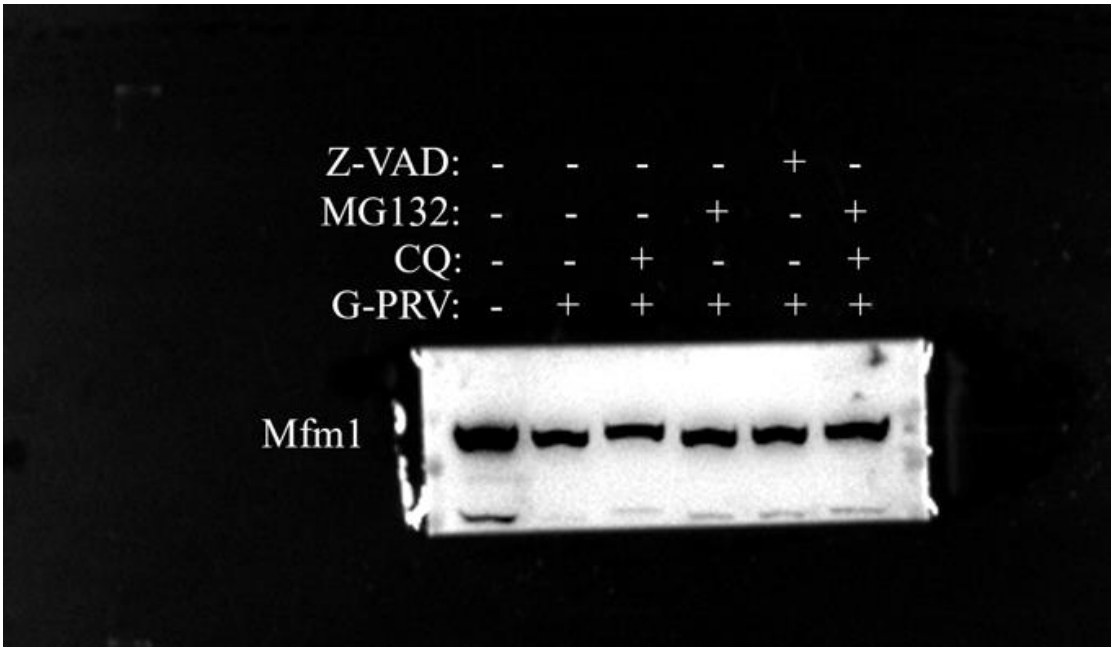

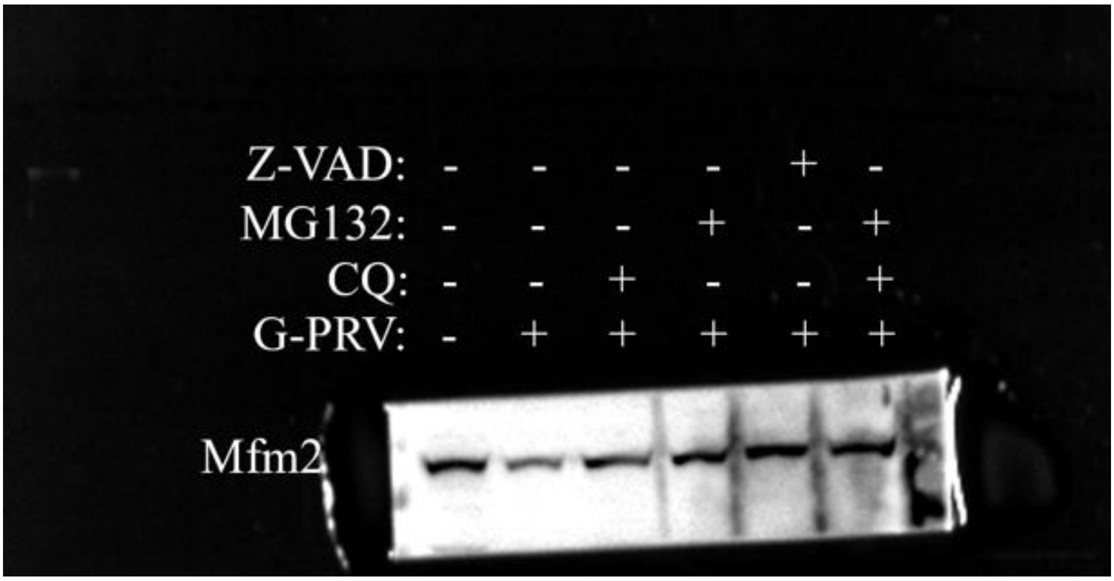

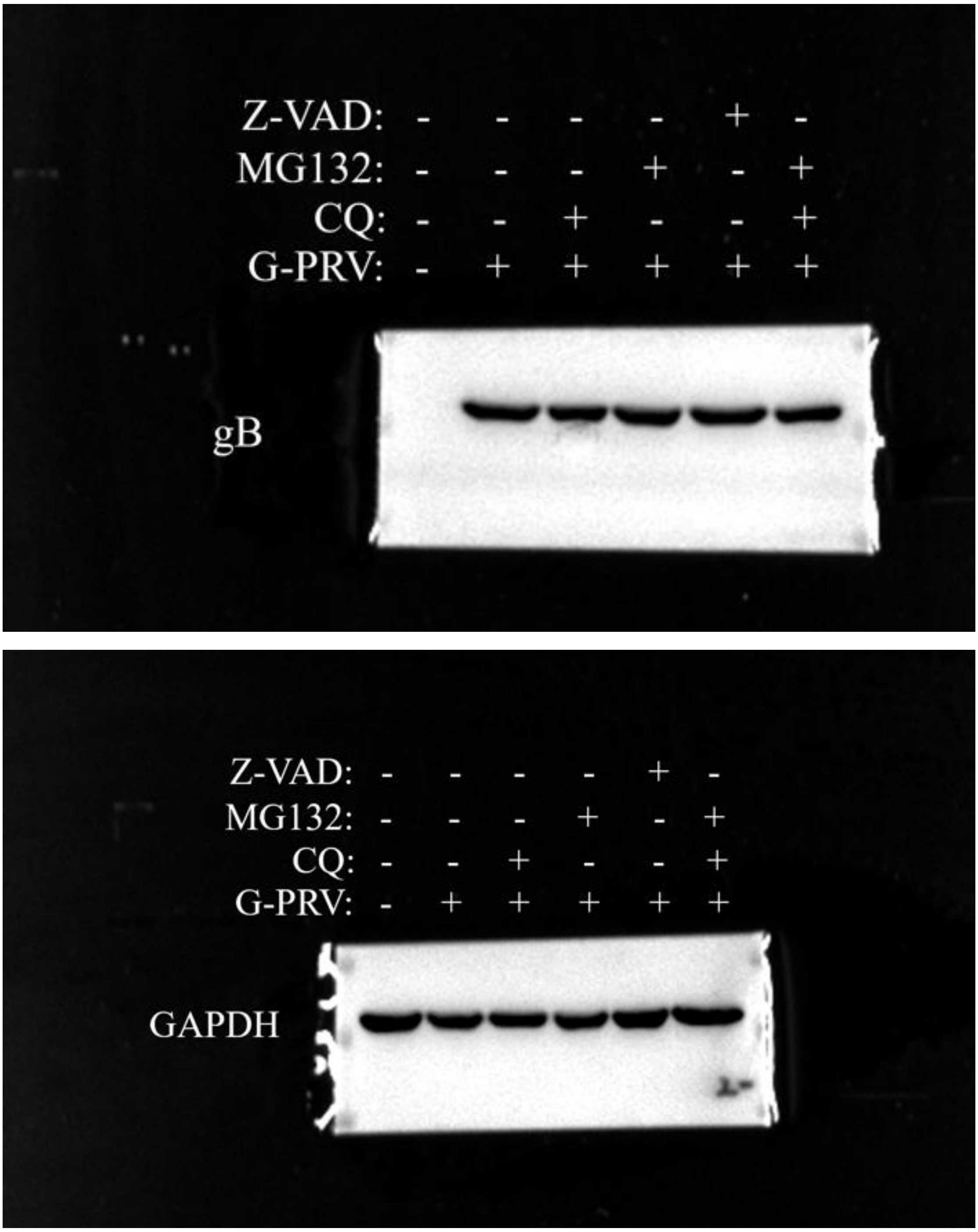

Figure S4D

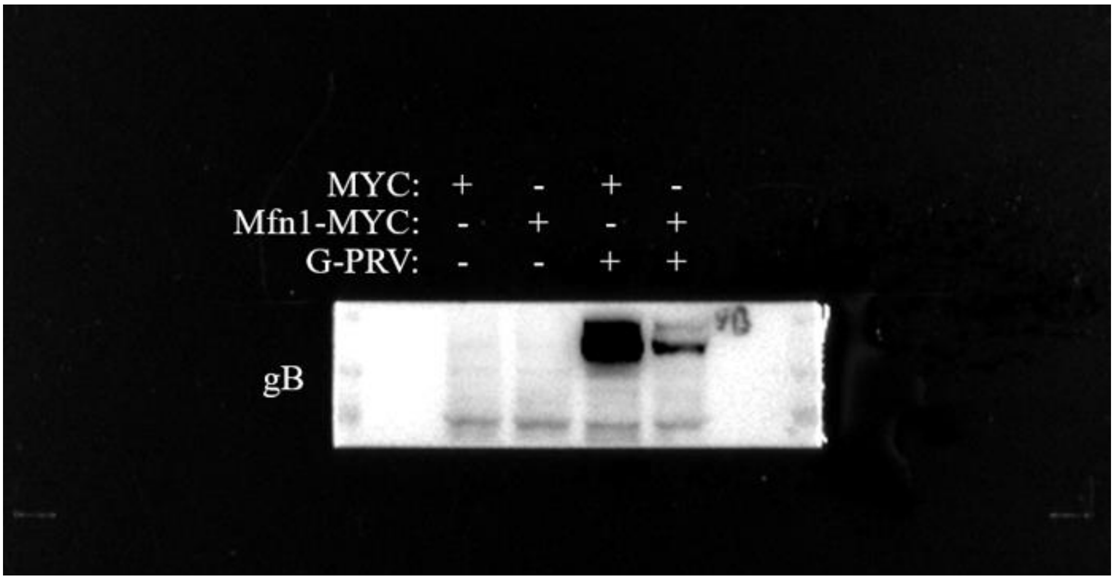
MYC: + - + -
Mfn1-MYC: - + - +
G-PRV: - - + +
gB

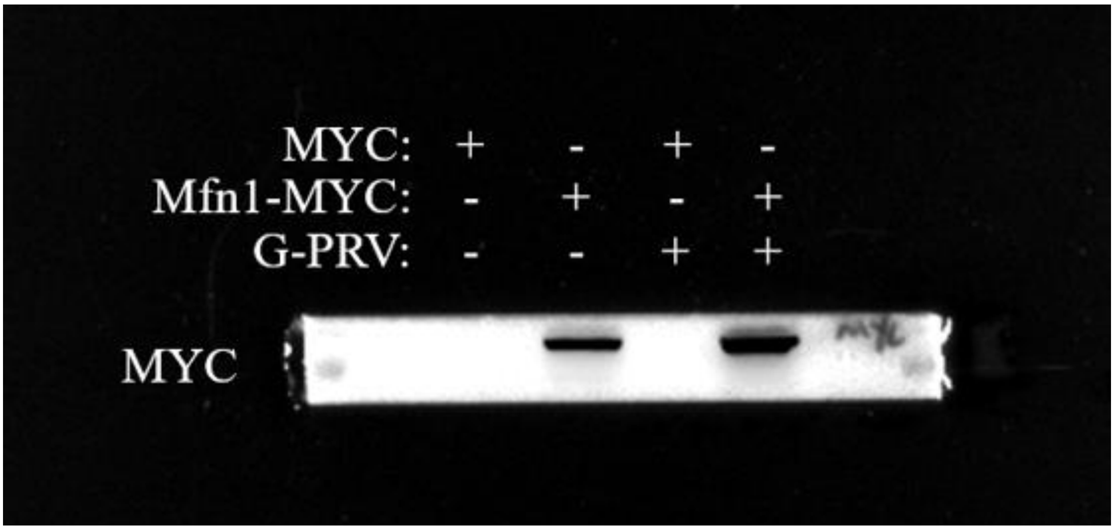
MYC: + - + -
Mfn1-MYC: - + - +
G-PRV: - - + +
MYC

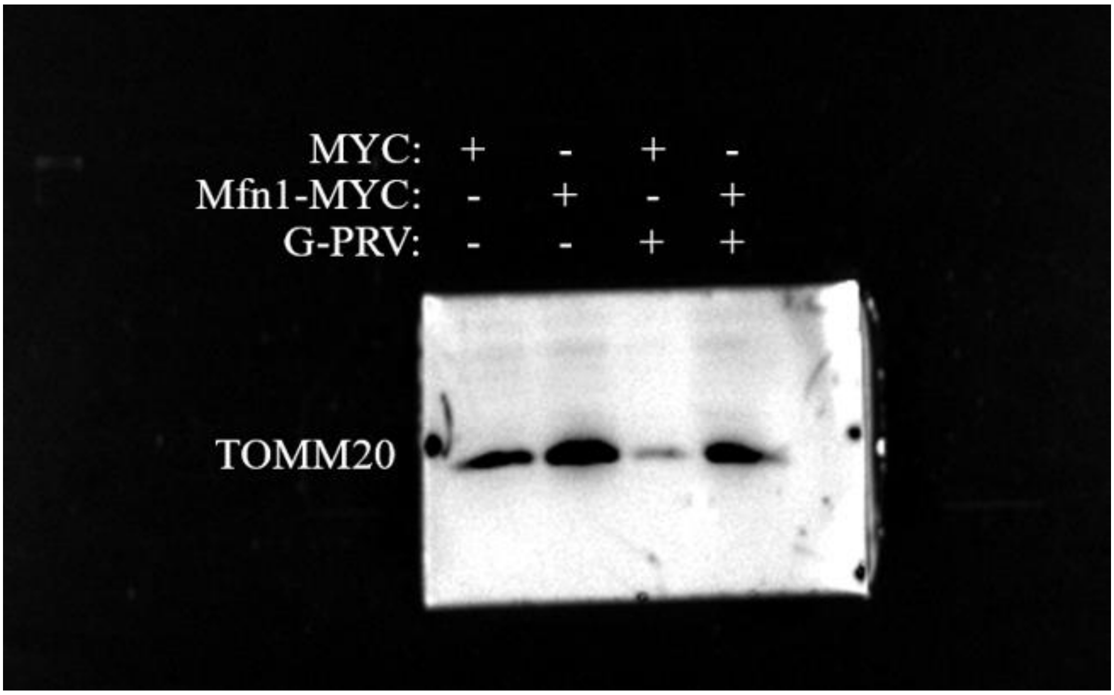
MYC: + - + -
Mfn1-MYC: - + - +
G-PRV: - - + +
TOMM20

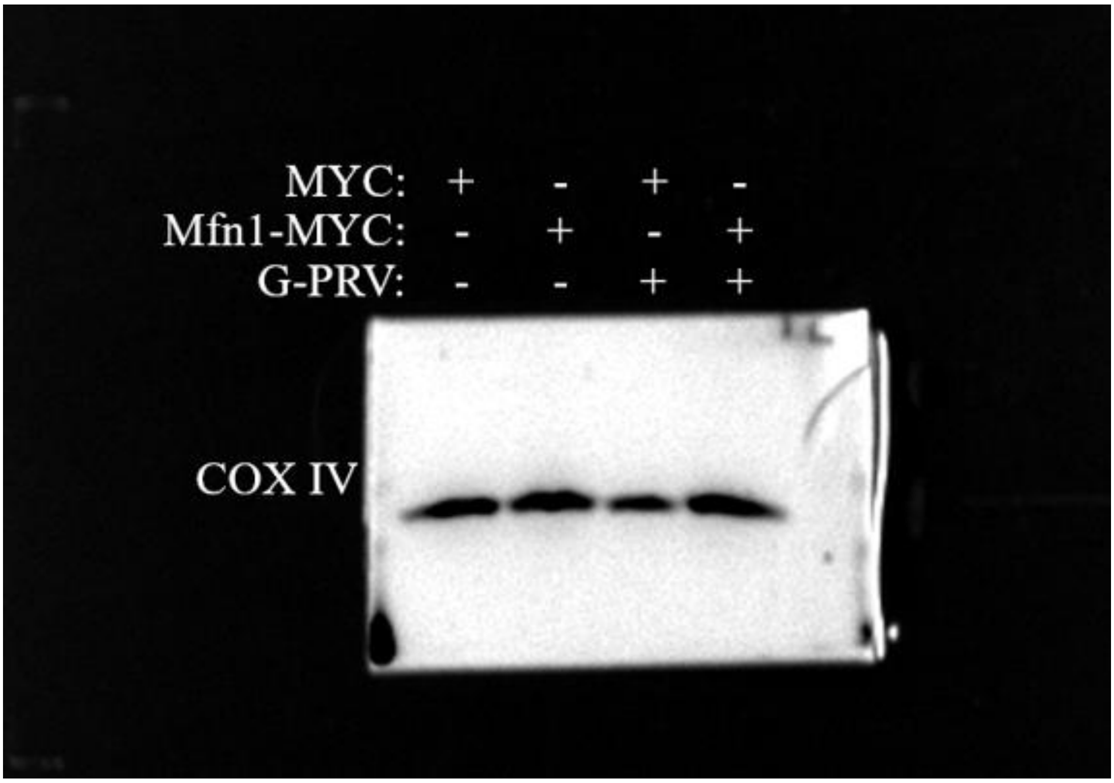
MYC: + - + -
Mfn1-MYC: - + - +
G-PRV: - - + +
COX IV

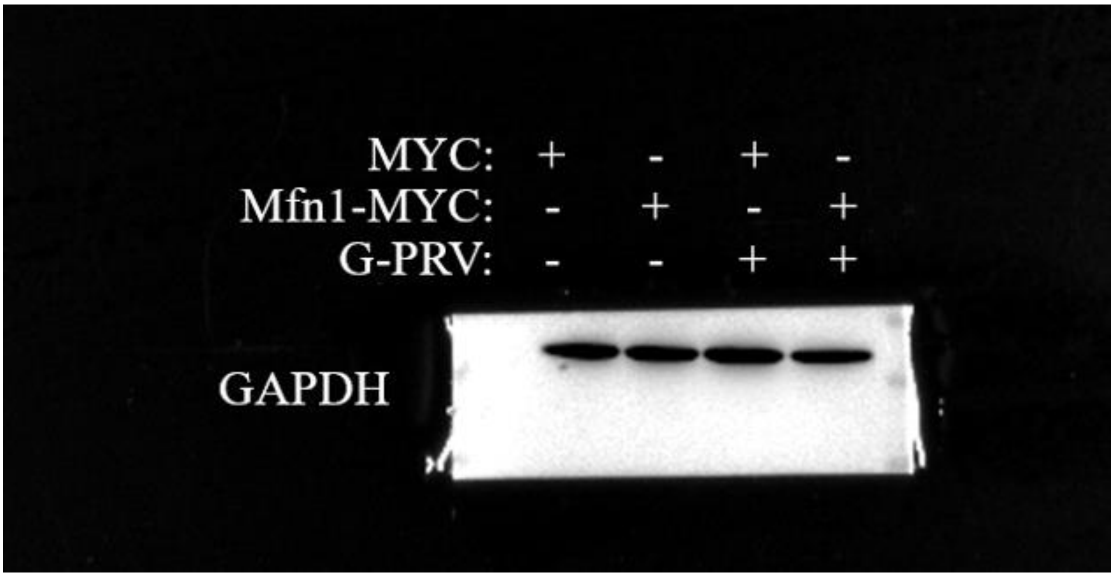
MYC: + - + -
Mfn1-MYC: - + - +
G-PRV: - - + +
GAPDH

Figure S4D repeat 2

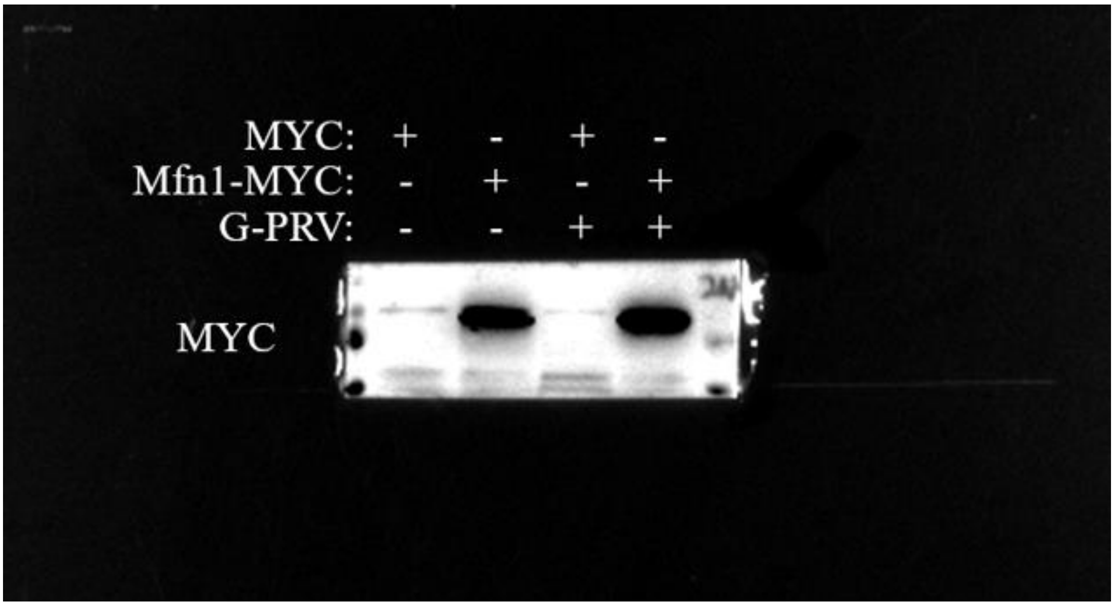

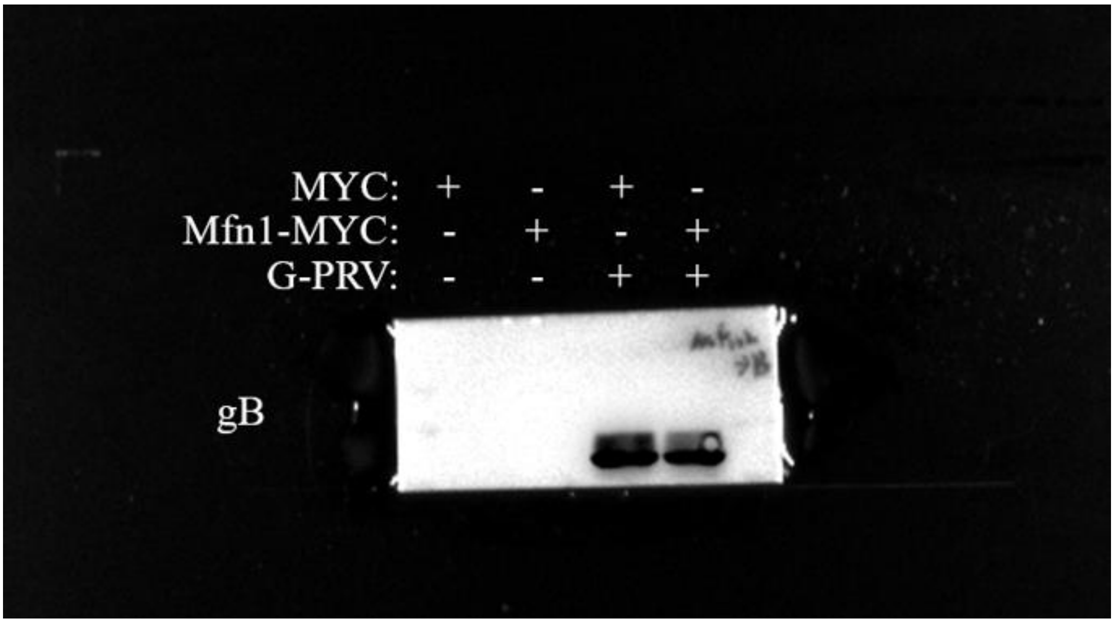

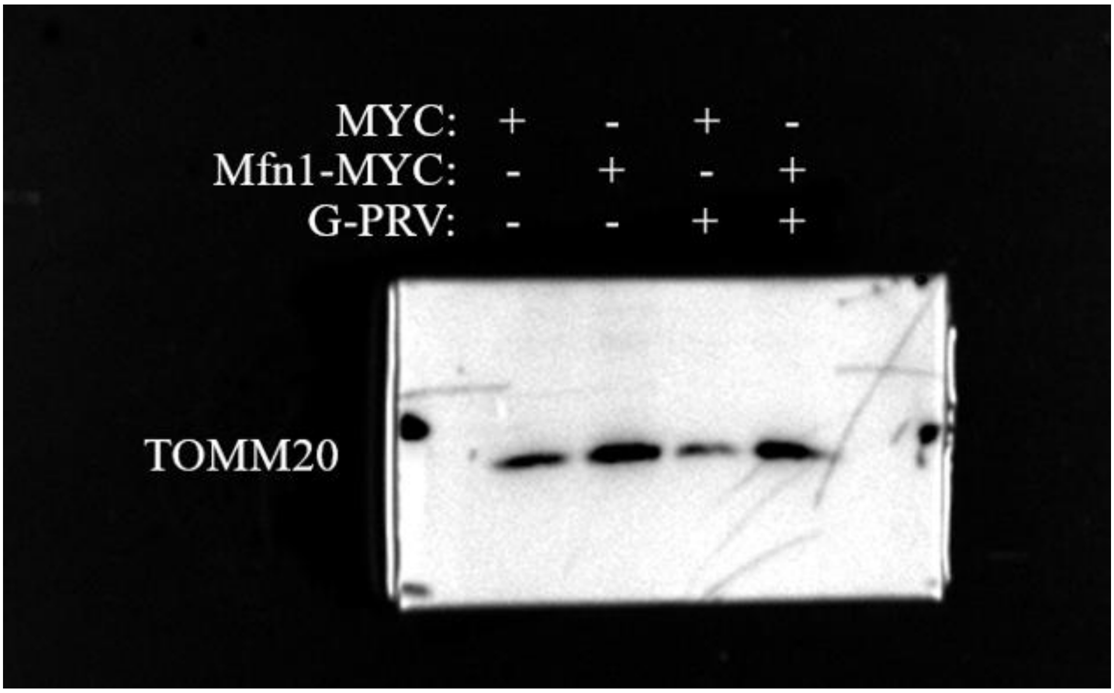
MYC: + - + -
Mfn1-MYC: - + - +
G-PRV: - - + +
TOMM20

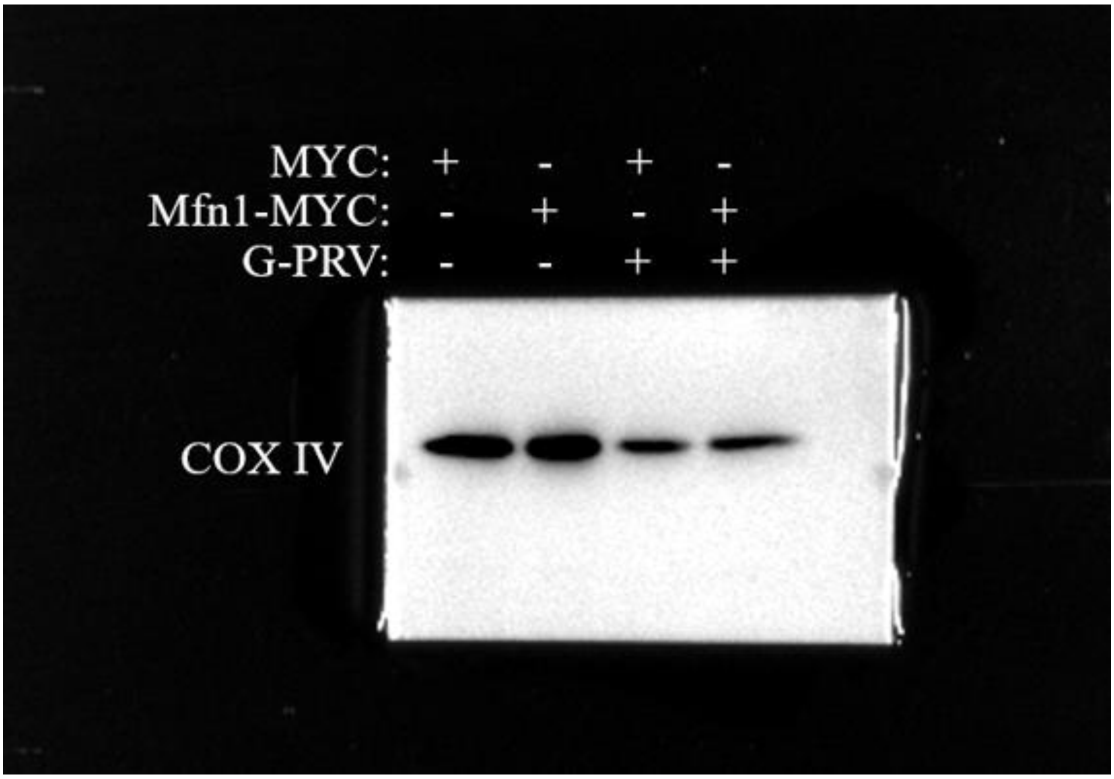
MYC: + - + -
Mfn1-MYC: - + - +
G-PRV: - - + +
COX IV

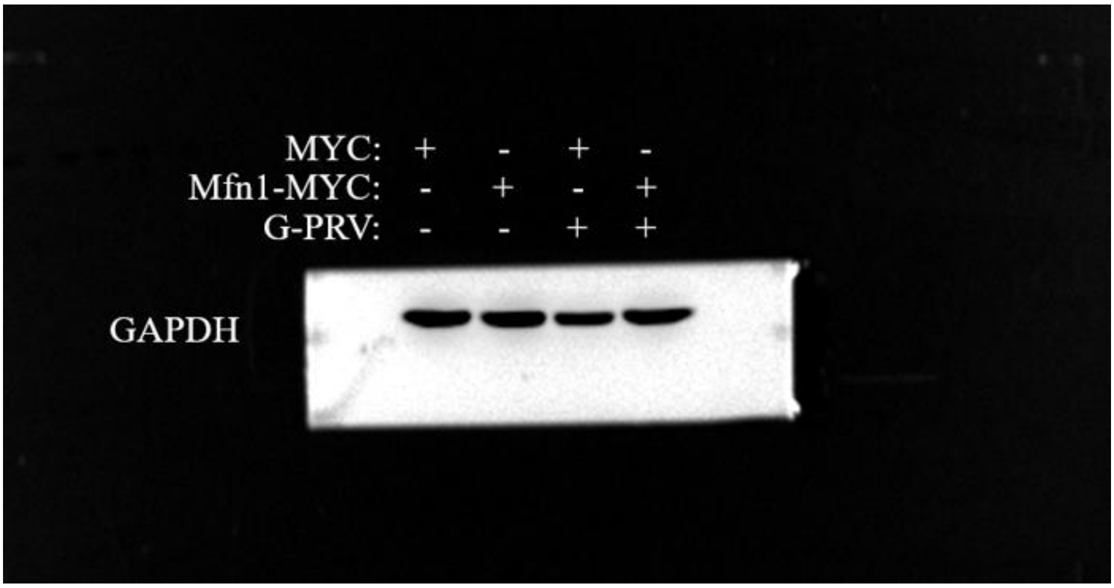

Figure S4D repeat 3

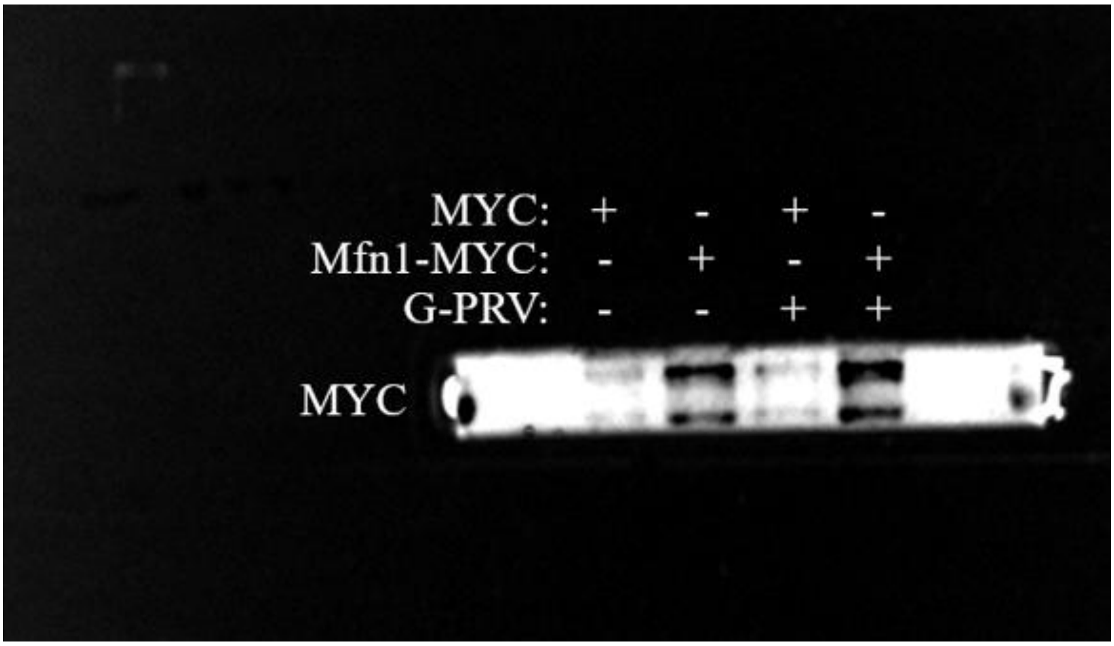

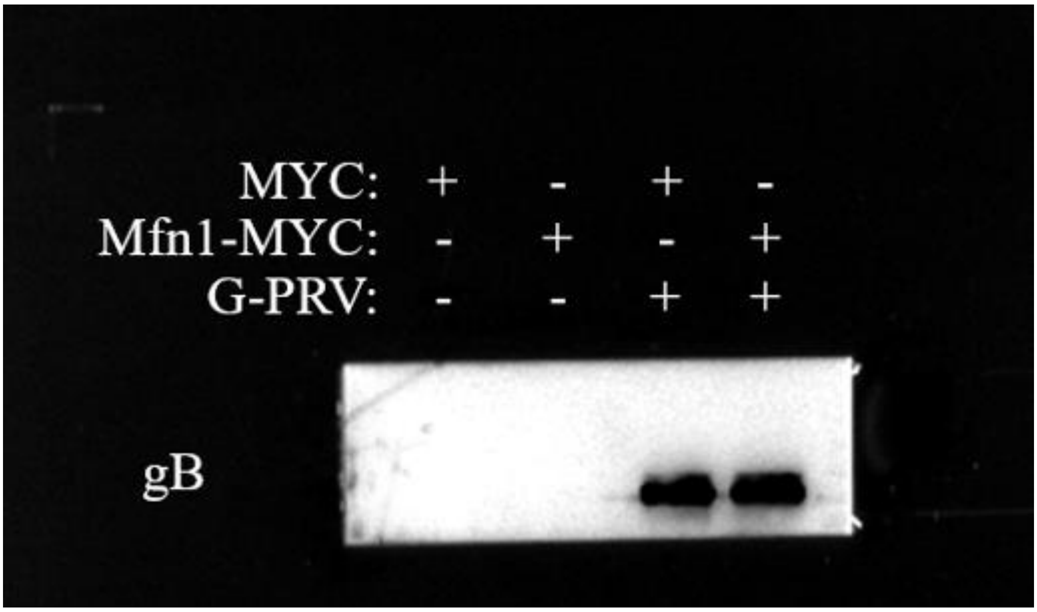
MYC: + - + -
Mfn1-MYC: - + - +
G-PRV: - - + +
gB

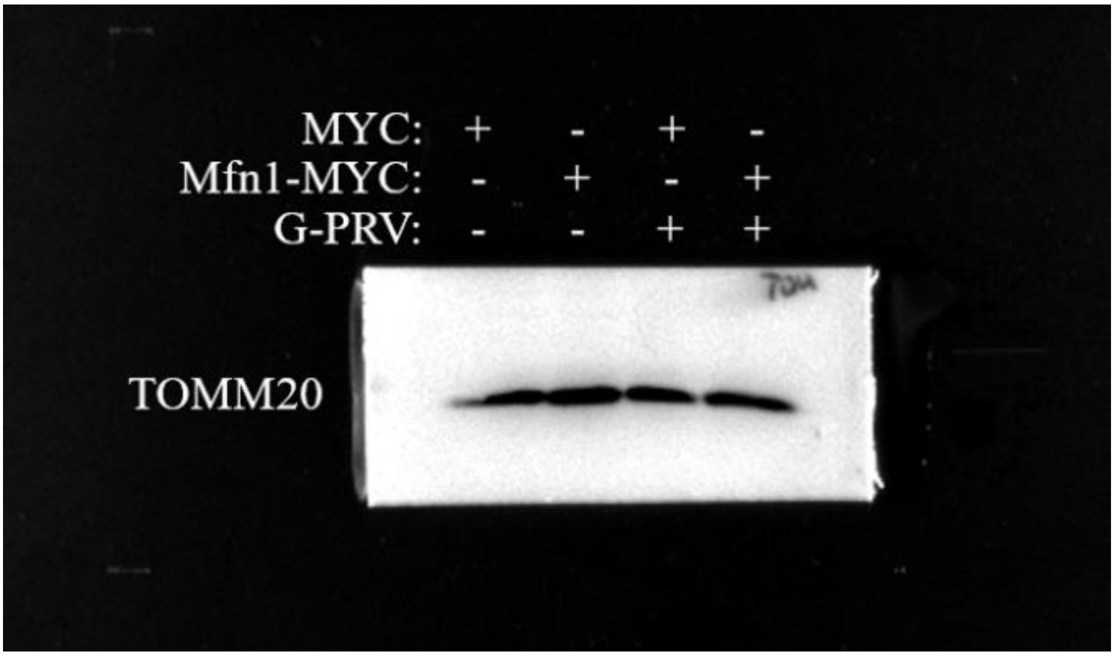
MYC: + - + -
Mfn1-MYC: - + - +
G-PRV: - - + +
TOMM20

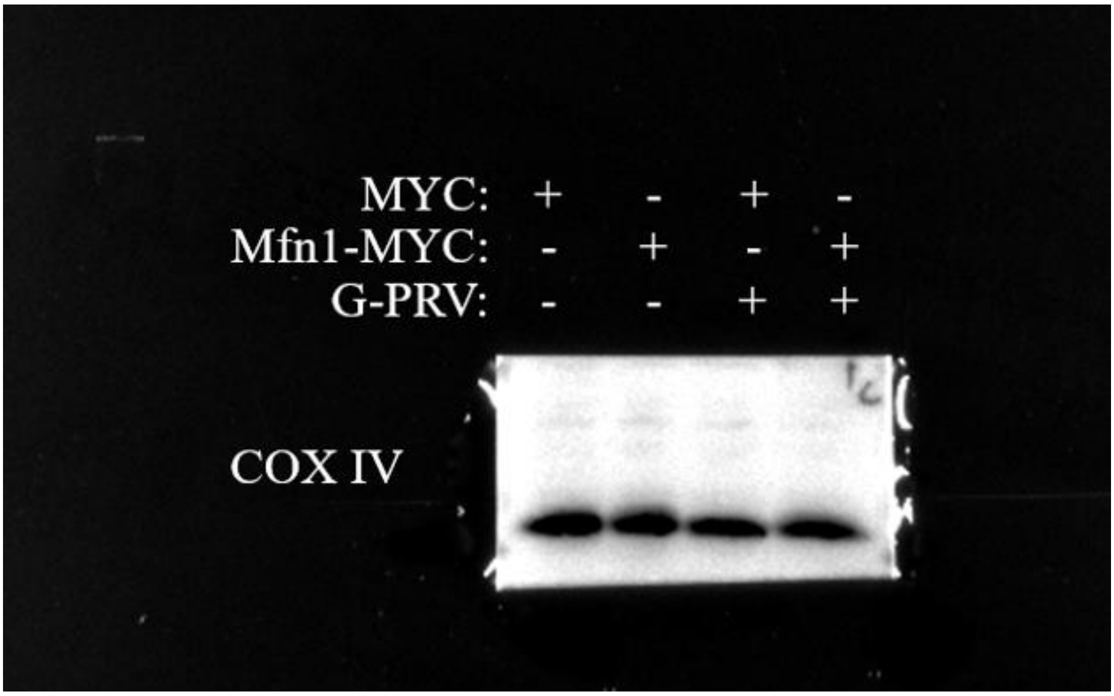

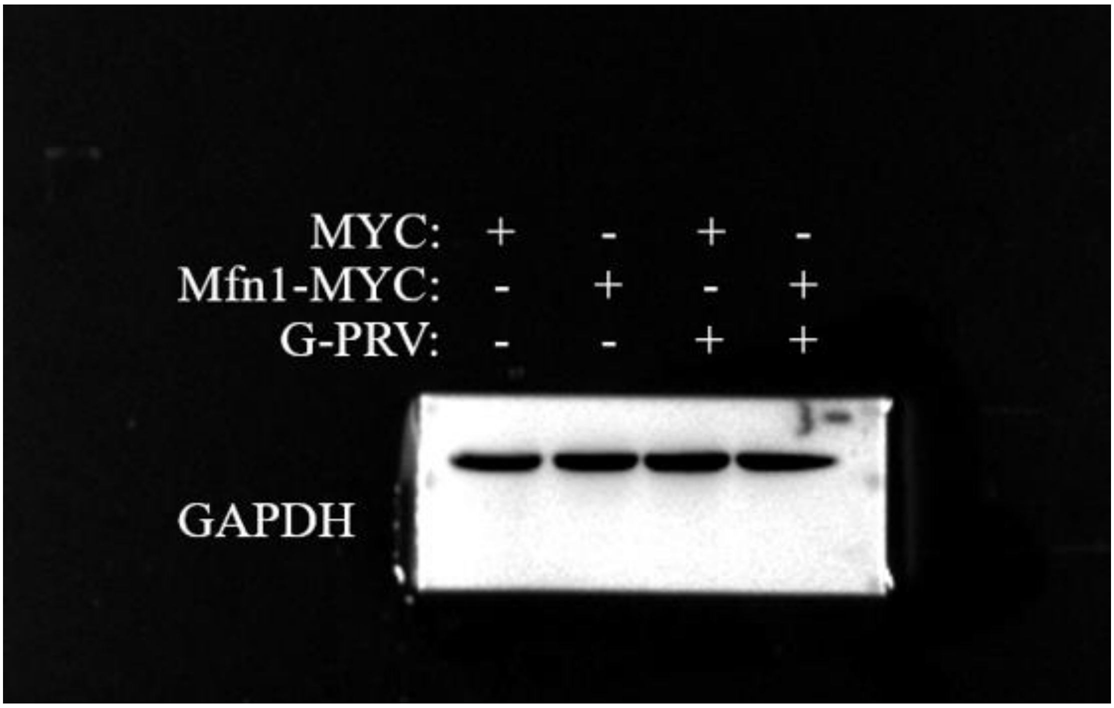

Figure S4E

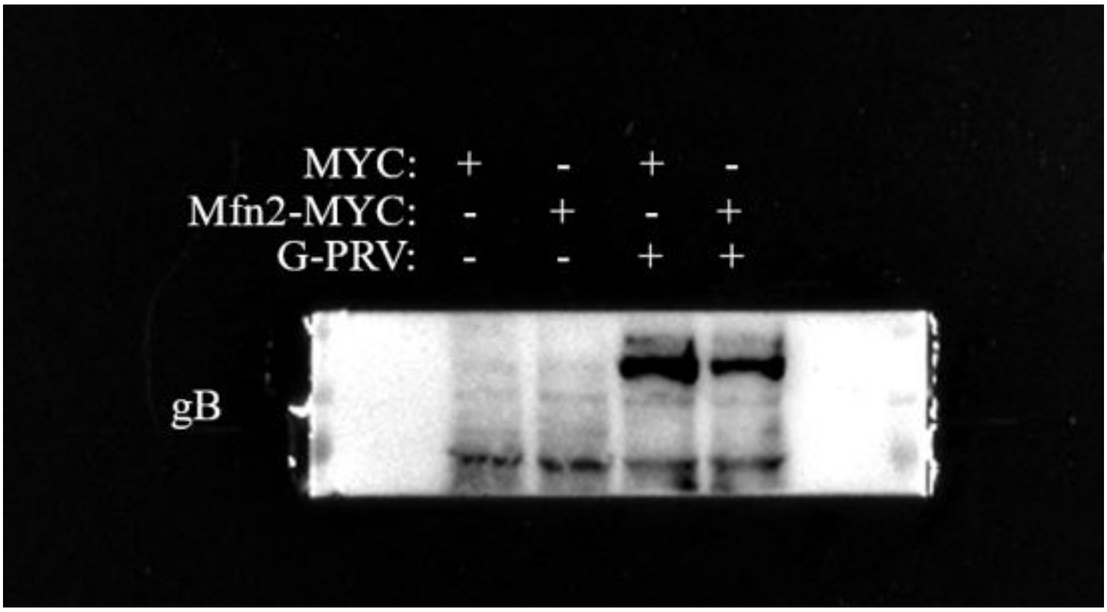
MYC: + - + -
Mfn2-MYC: - + - +
G-PRV: - - + +
gB

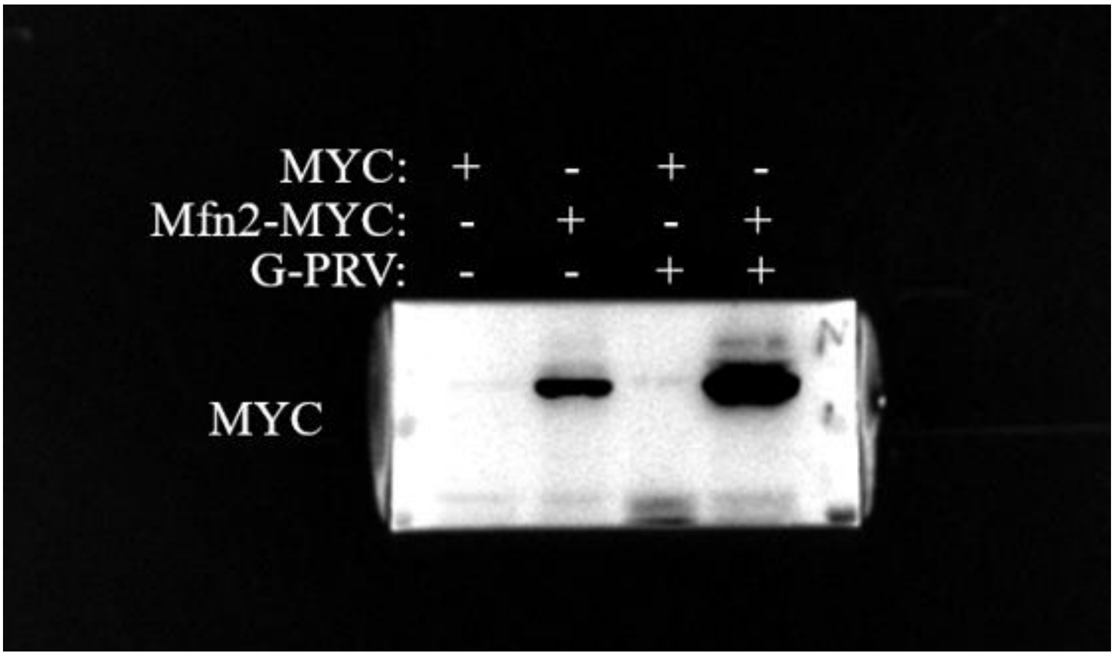
MYC: + - + -
Mfn2-MYC: - + - +
G-PRV: - - + +
MYC

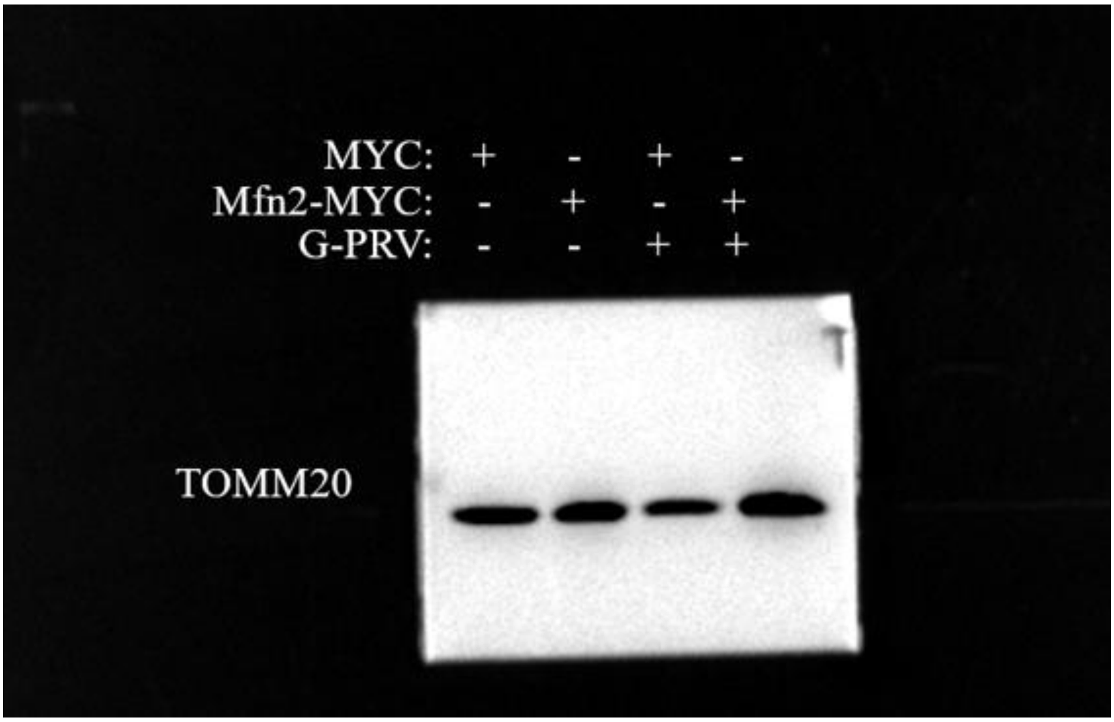
MYC: + - + -
Mfn2-MYC: - + - +
G-PRV: - - + +
TOMM20

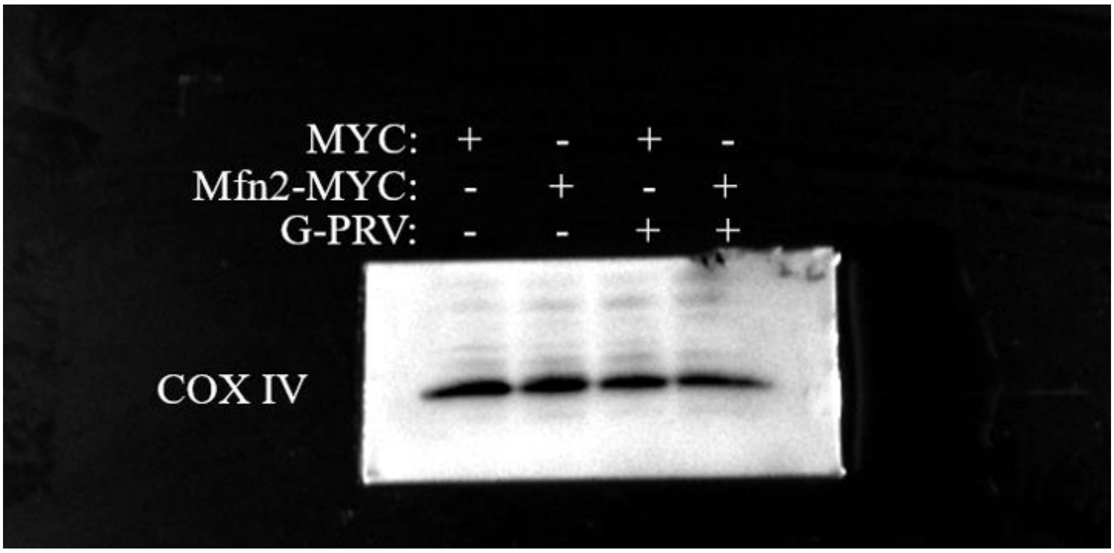
MYC: + - + -
Mfn2-MYC: - + - +
G-PRV: - - + +
COX IV

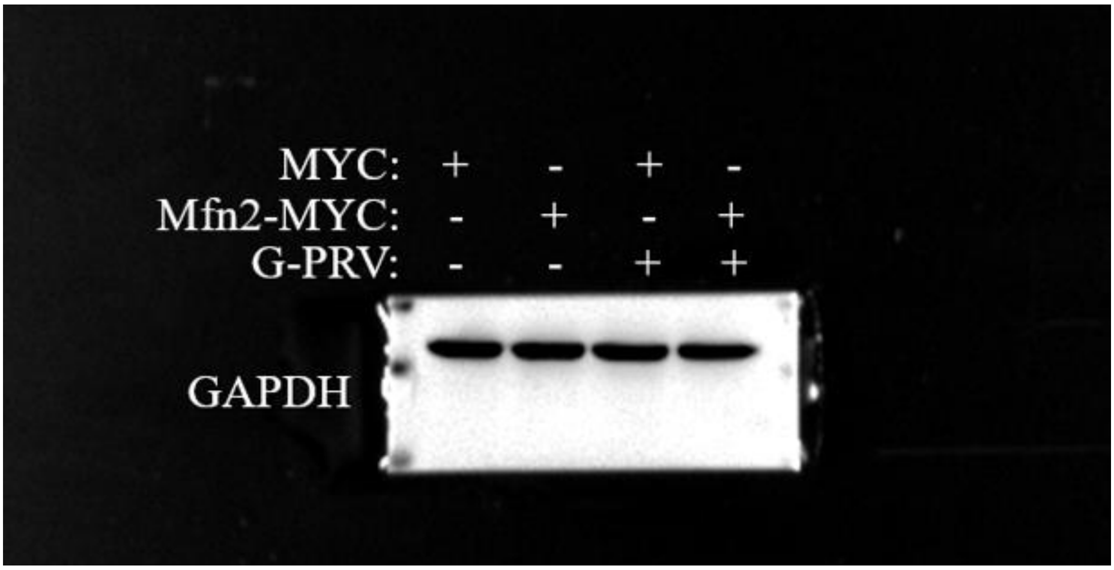

Figure S4E repeat 2

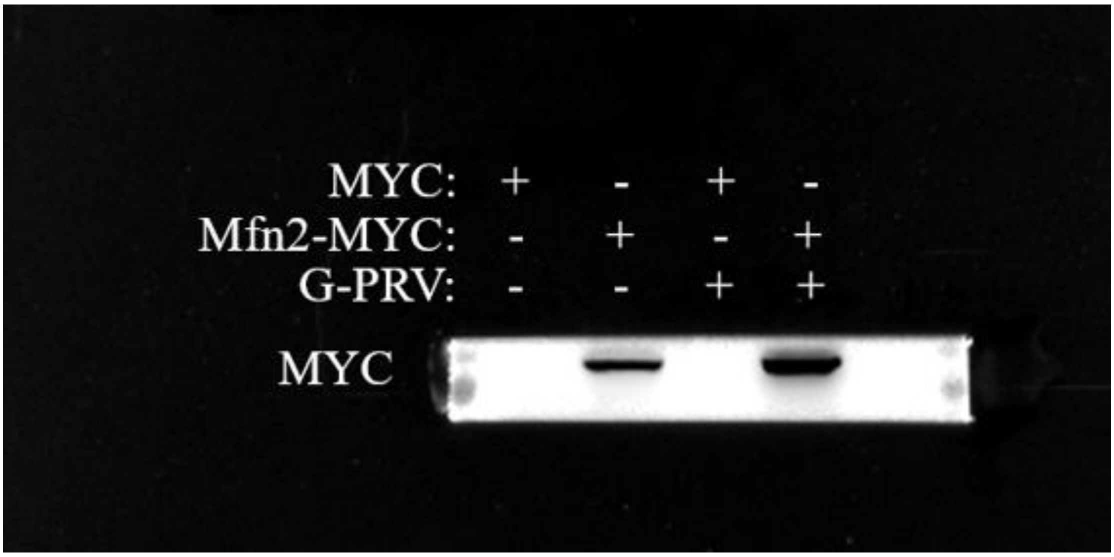

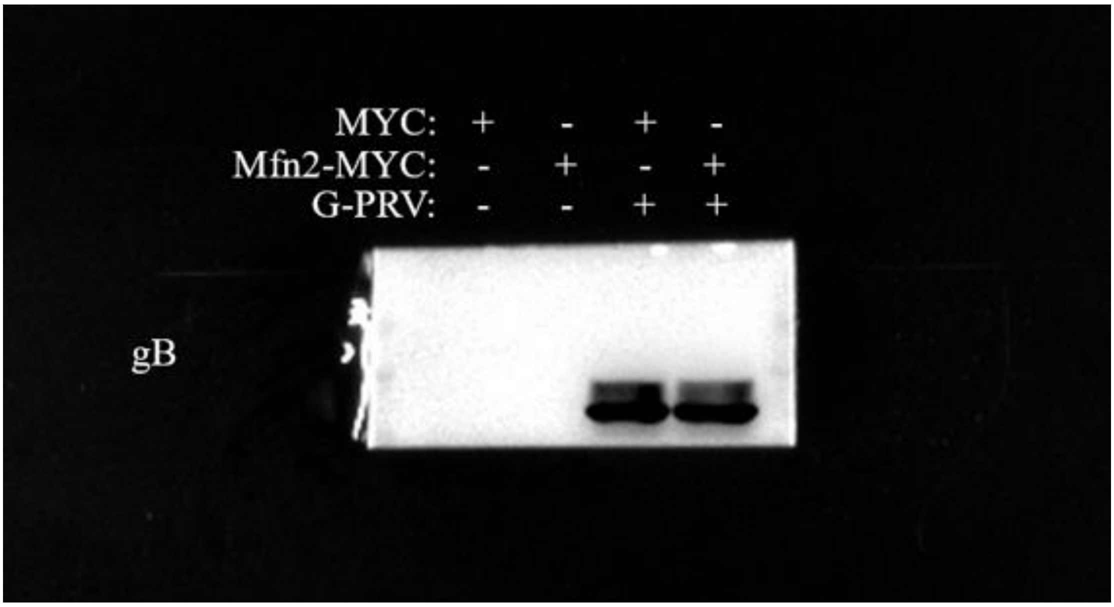
MYC: + - + -
Mfn2-MYC: - + - +
G-PRV: - - + +
gB

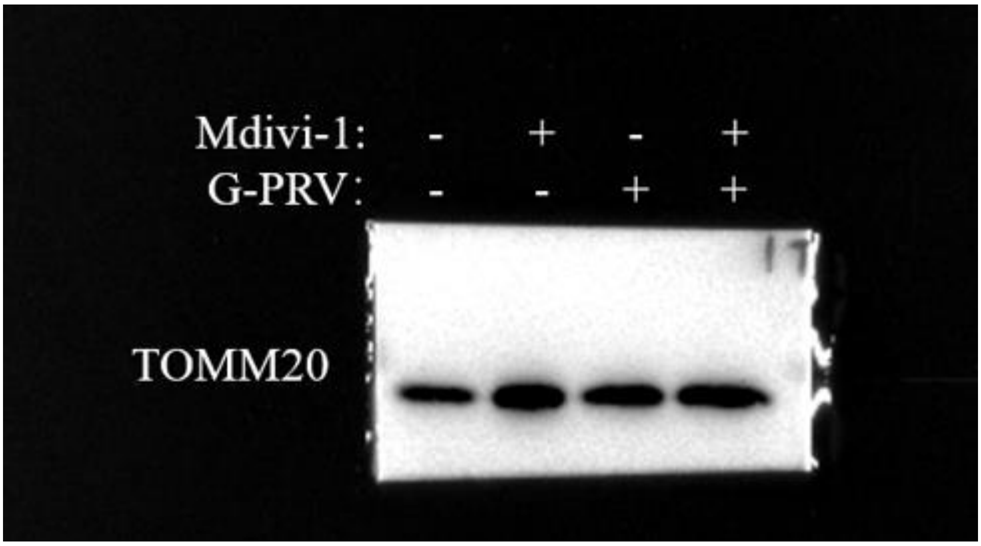
Mdivi-1: - + - +
G-PRV: - - + +
TOMM20

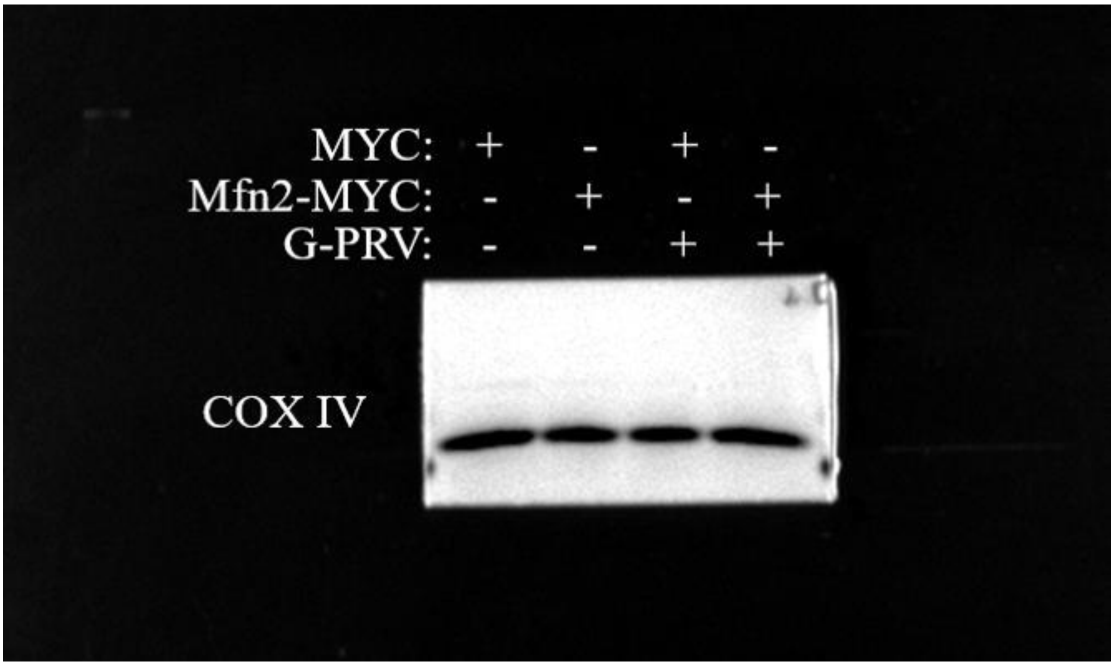
MYC: + - + -
Mfn2-MYC: - + - +
G-PRV: - - + +
COX IV

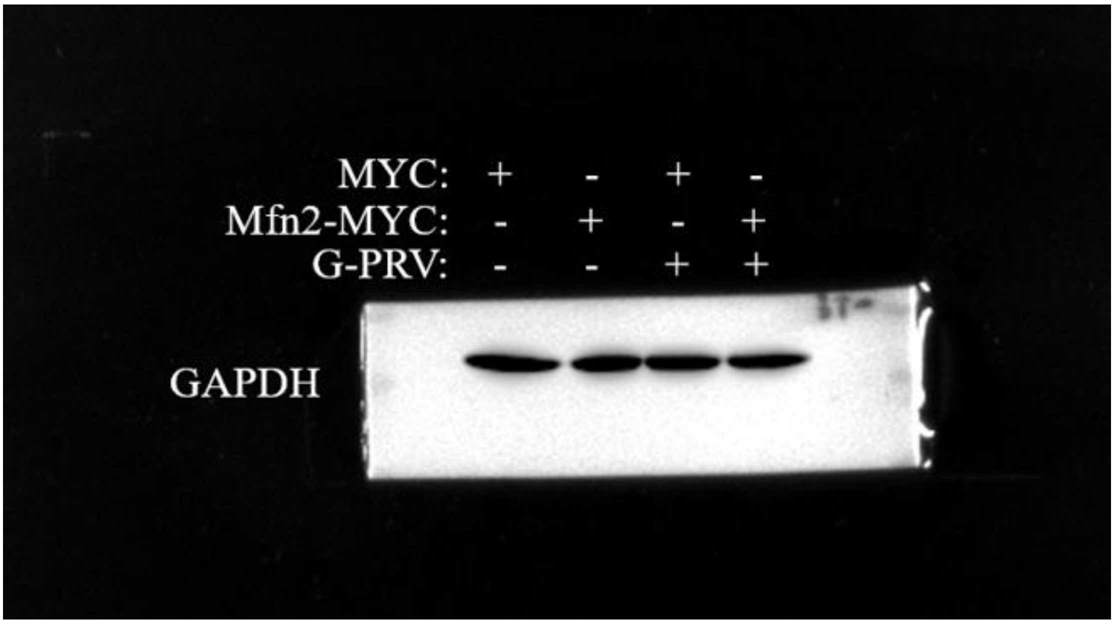

Figure S4E repeat 3

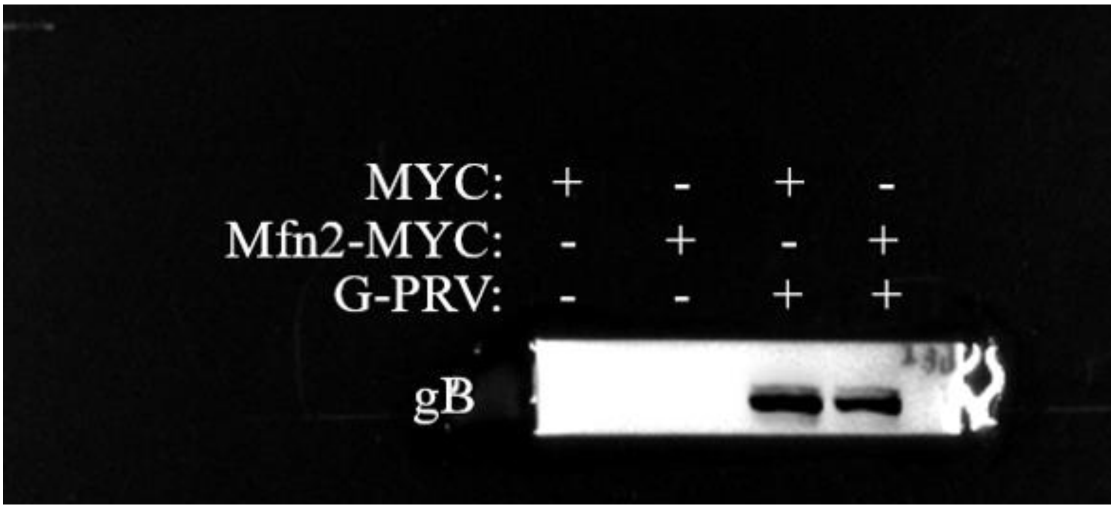

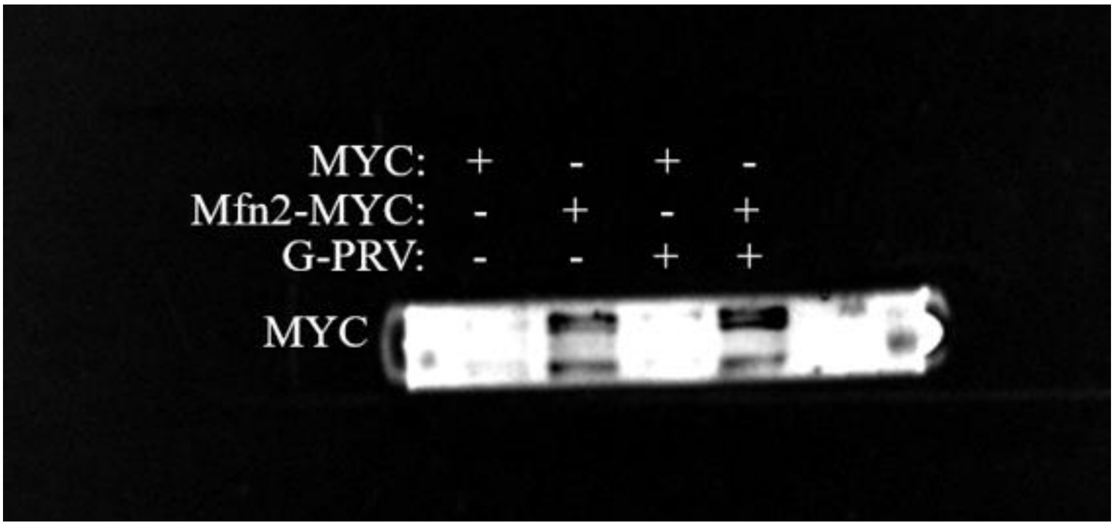

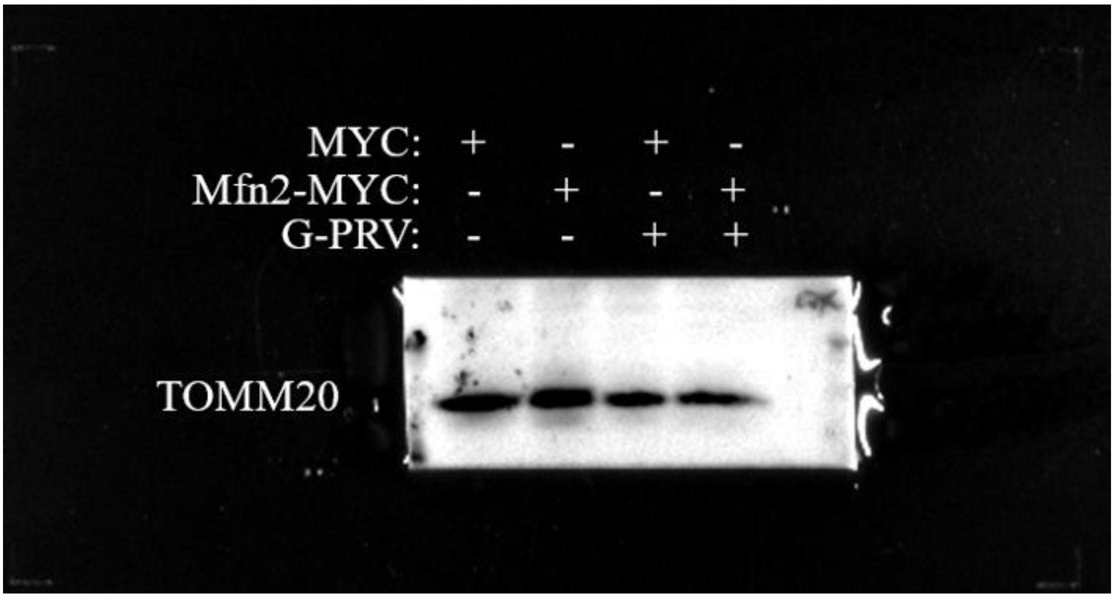
MYC: + - + -
Mfn2-MYC: - + - +
G-PRV: - - + +
TOMM20

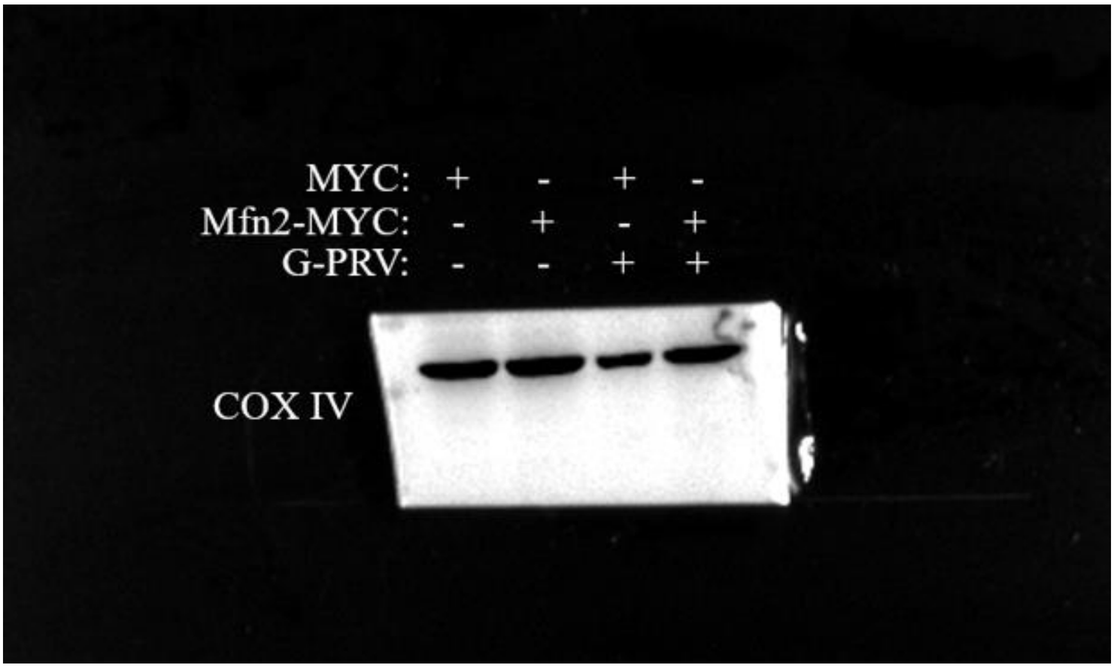
MYC: + - + -
Mfn2-MYC: - + - +
G-PRV: - - + +
COX IV

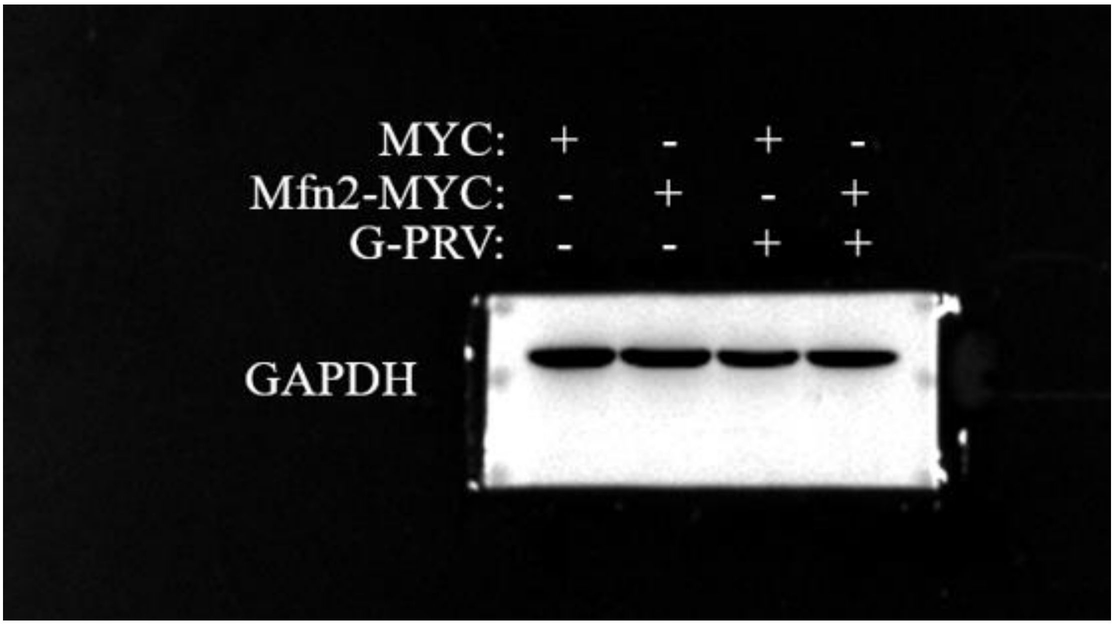

Figure S4F

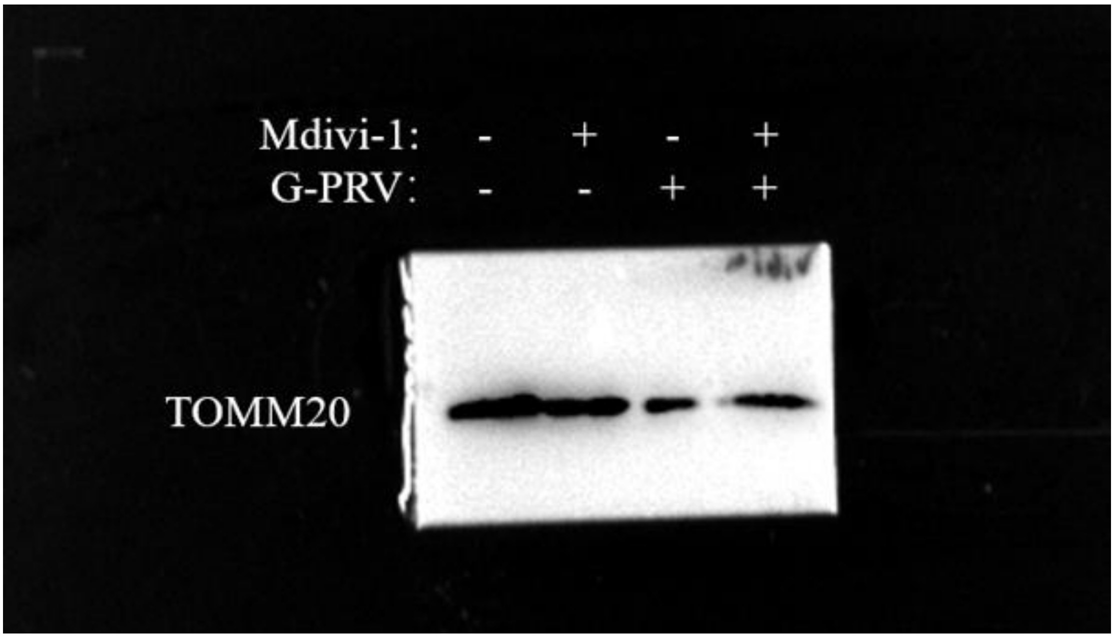

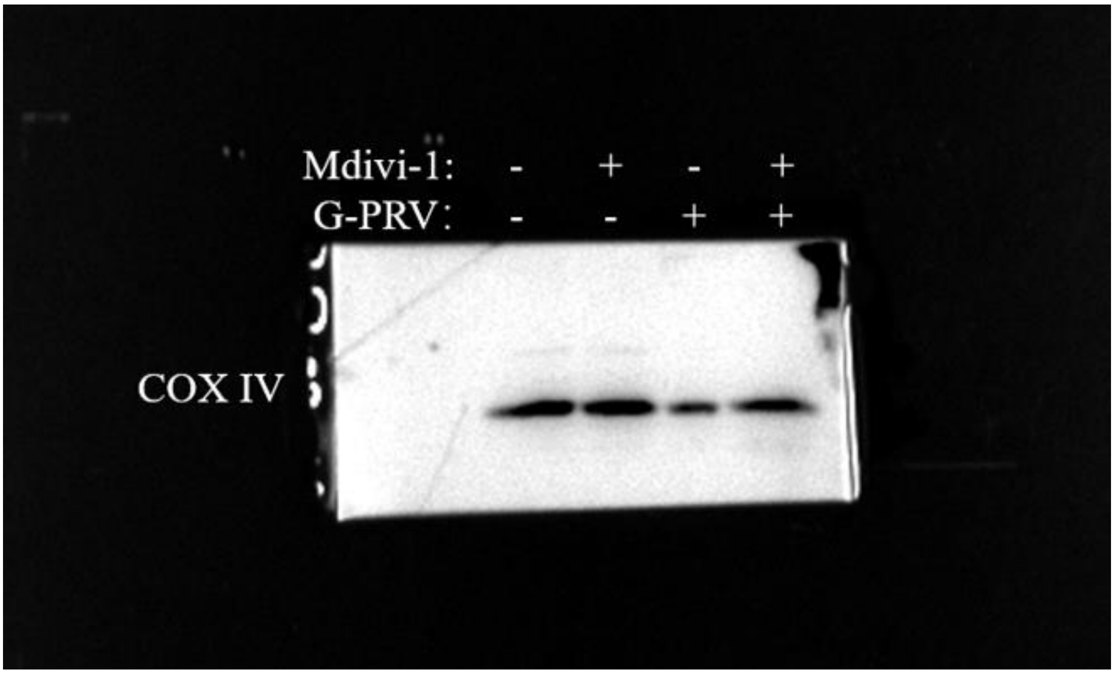

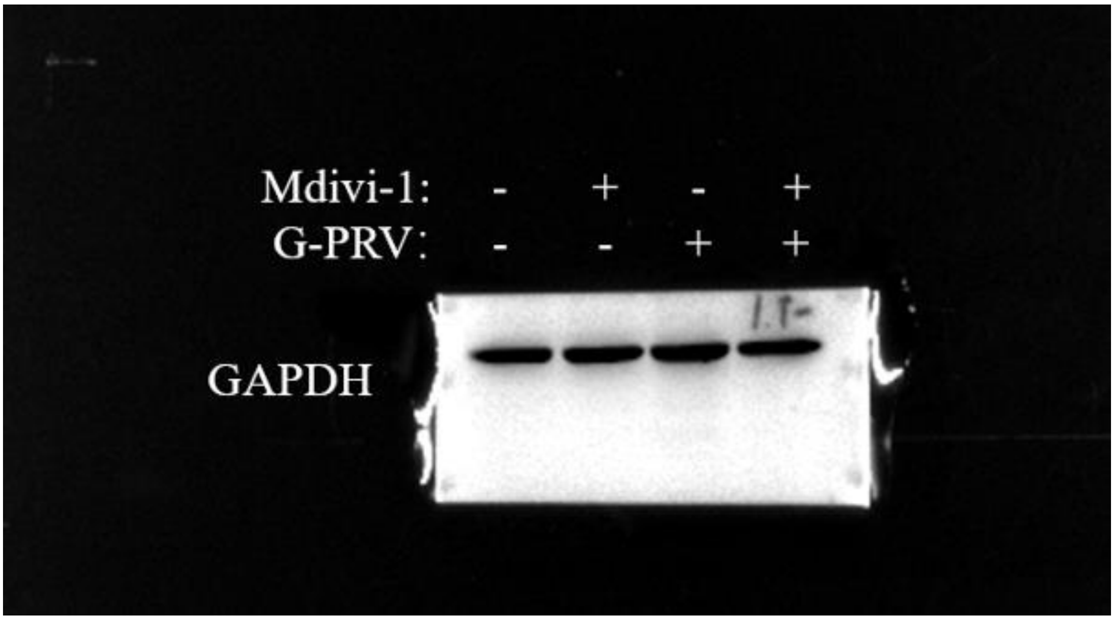

Figure S4F repeat 2

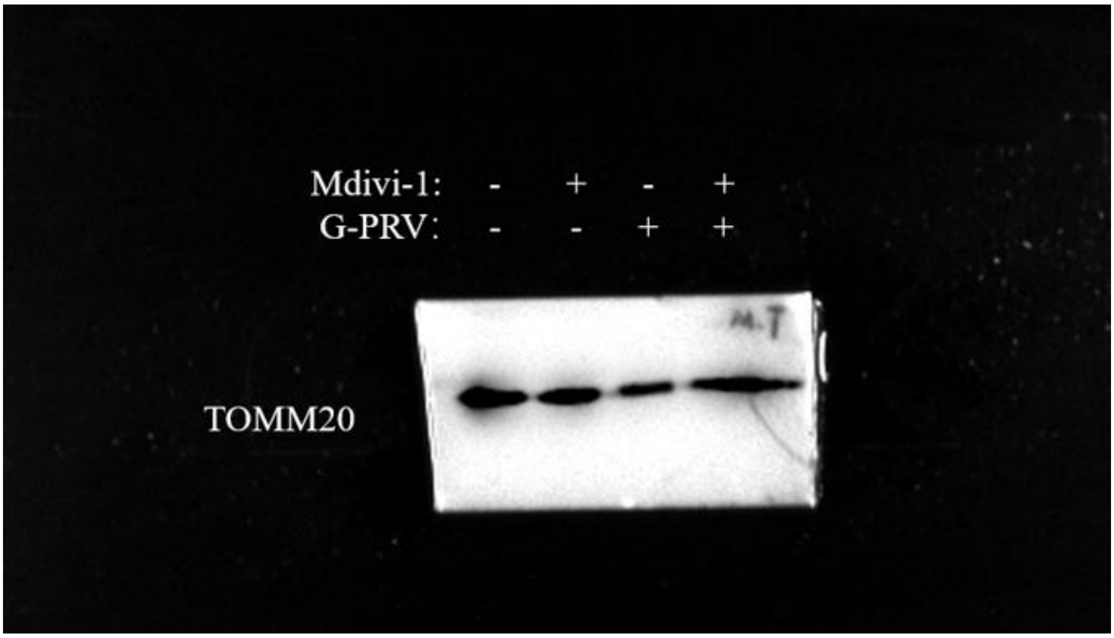
Mdivi-1: - + - +
G-PRV: - - + +
TOMM20

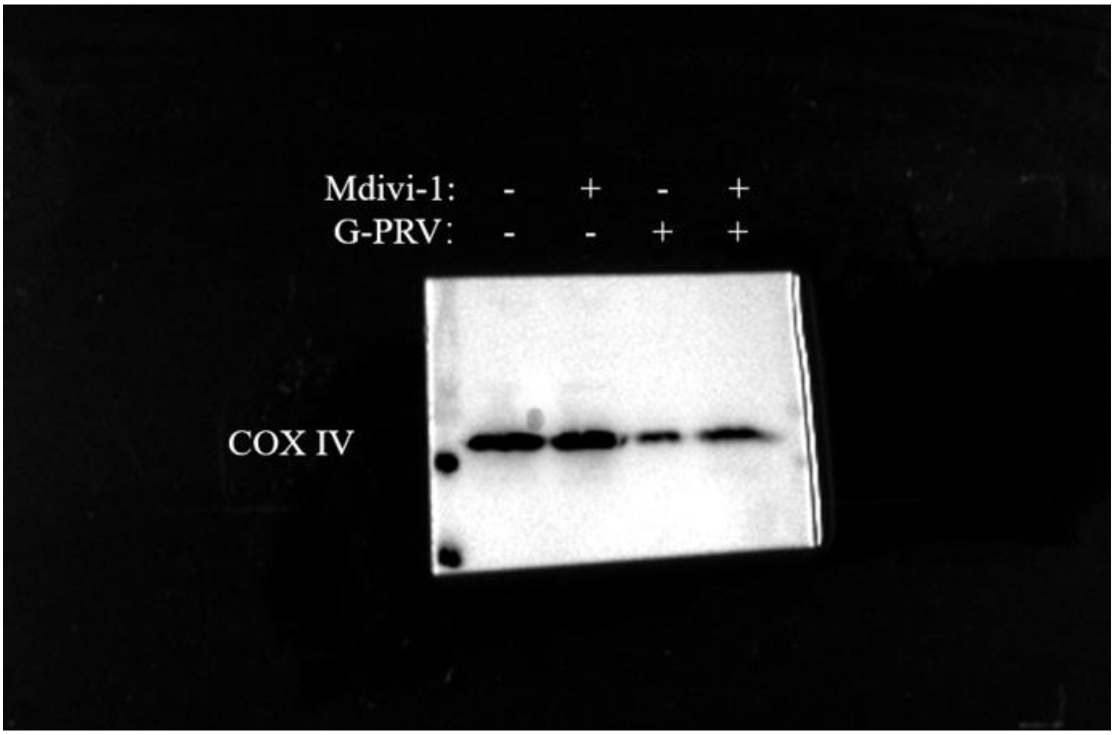
Mdivi-1: - + - +
G-PRV: - - + +
COX IV

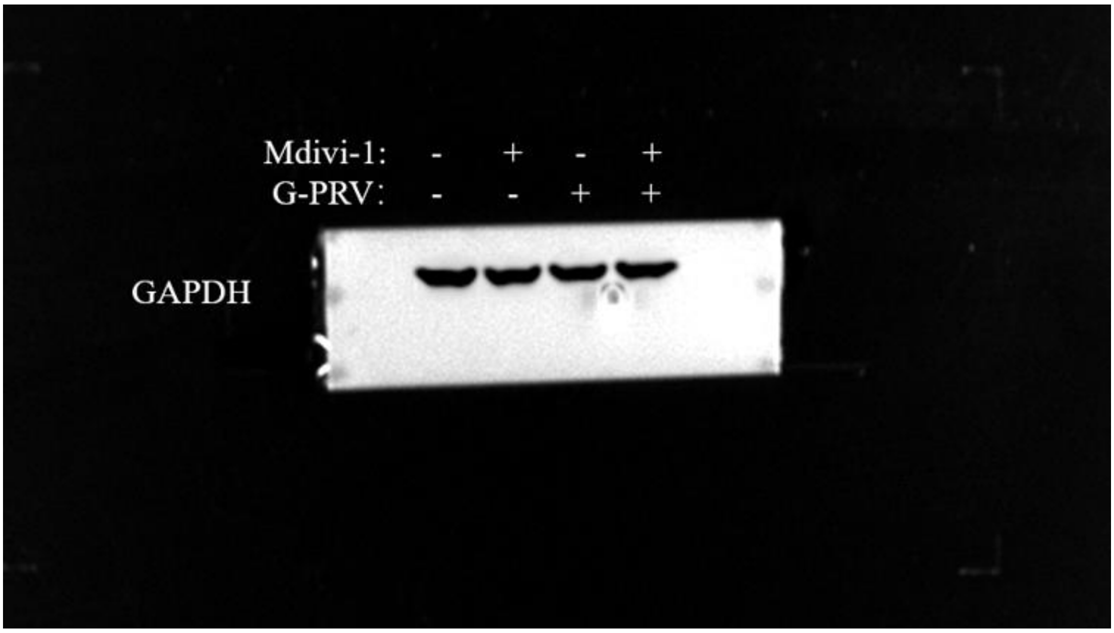

Figure S4F repeat 3

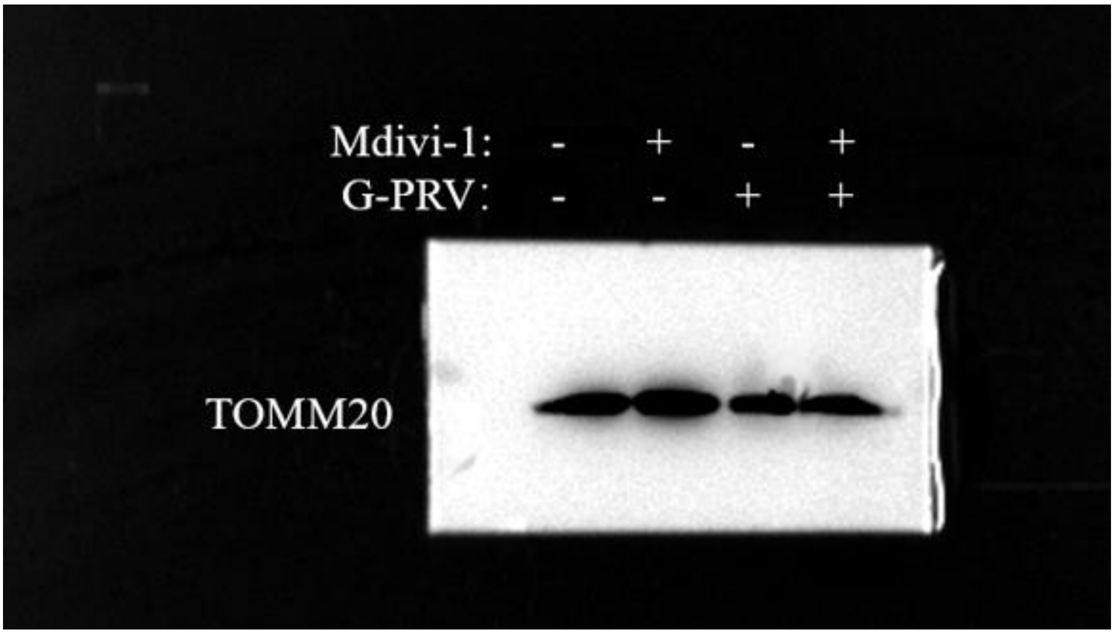

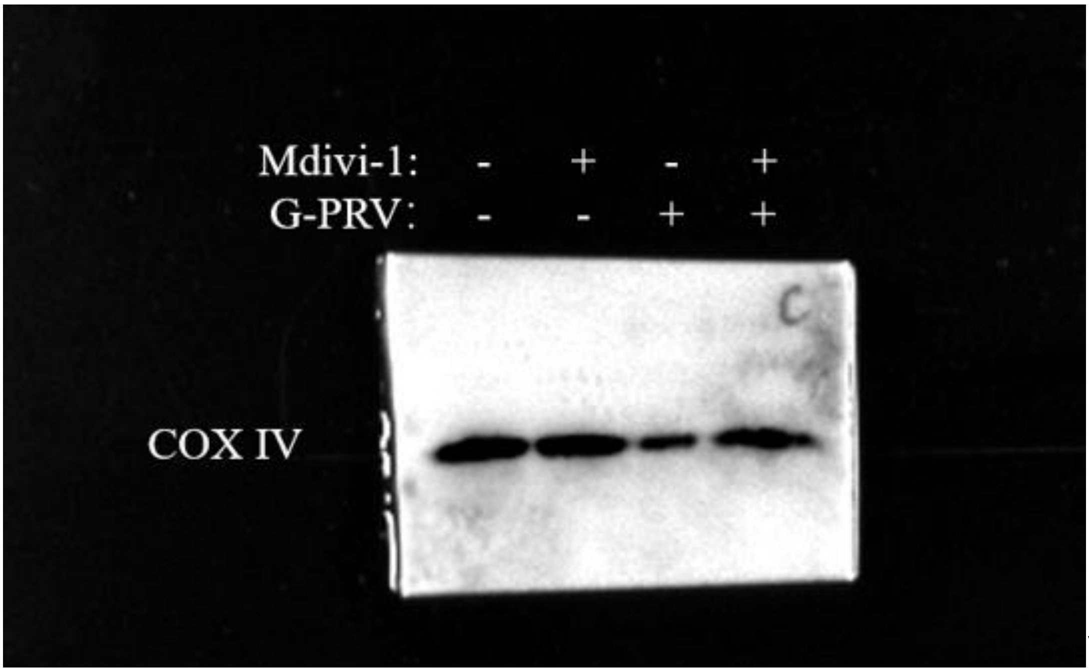

\

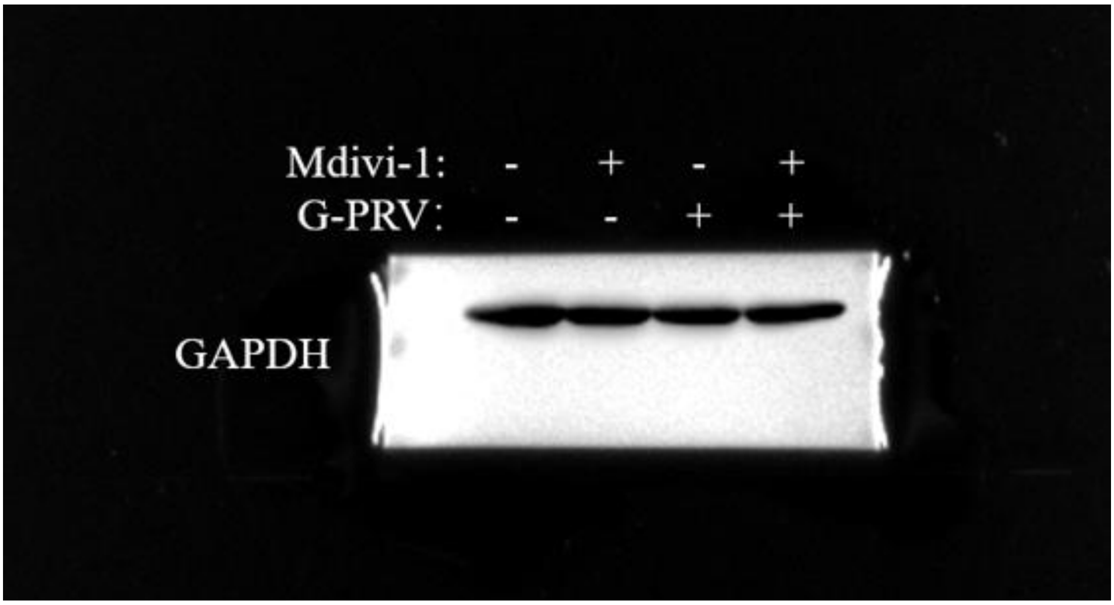

Figure S5B

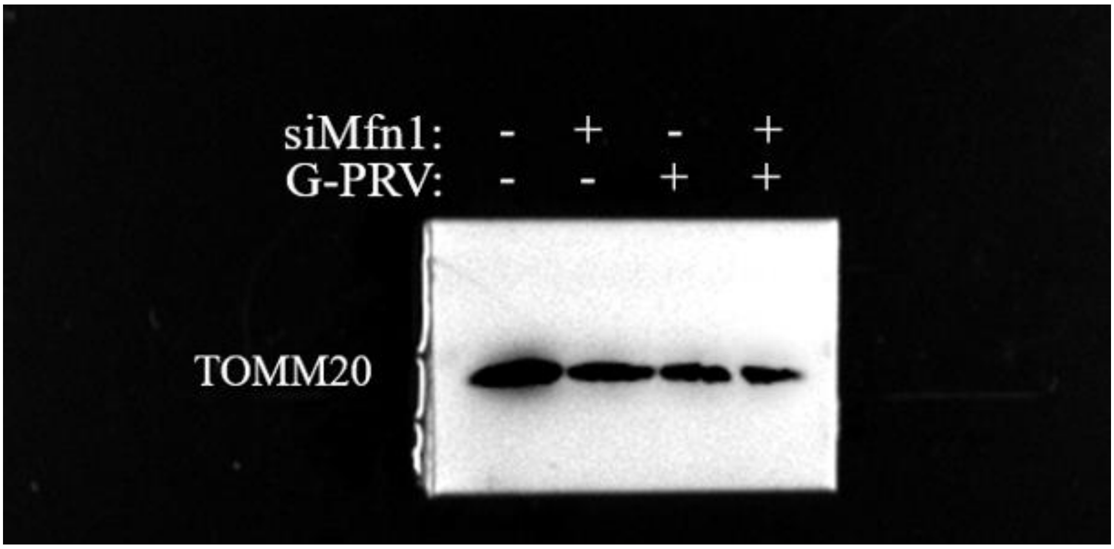
siMfn1: - + - +
G-PRV: - - + +
TOMM20

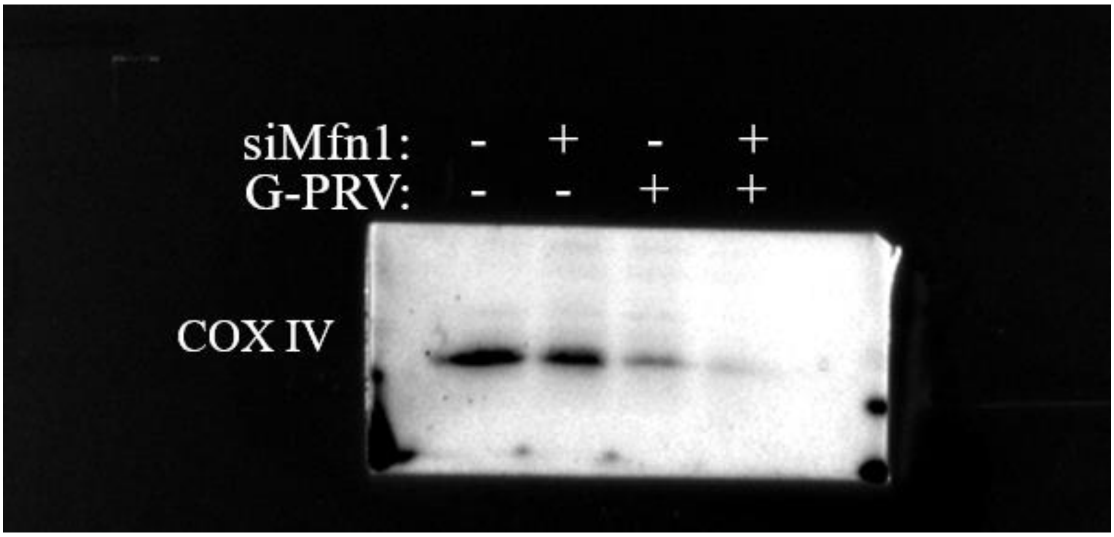
siMfn1: - + - +
G-PRV: - - + +
COX IV

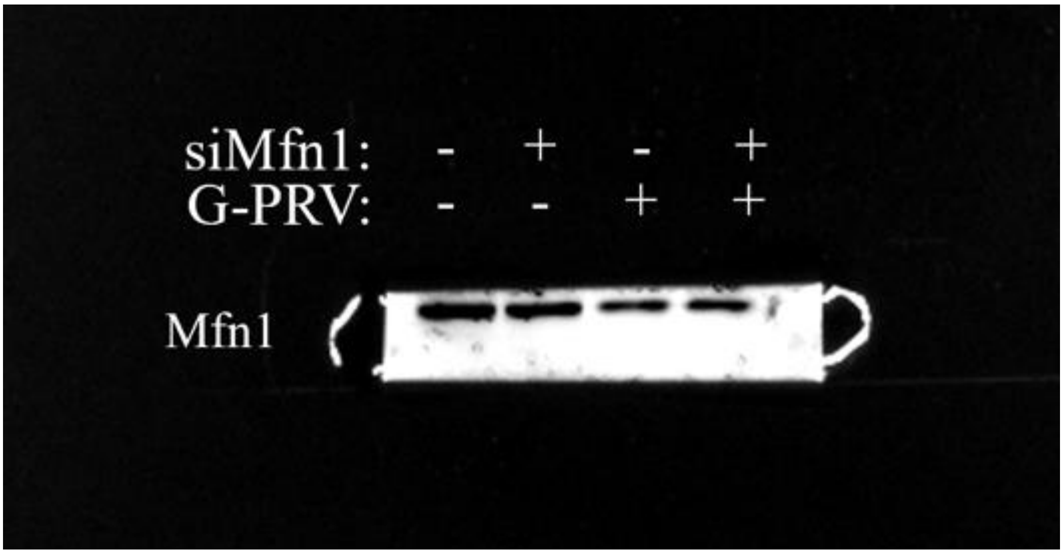
siMfn1: - + - +
G-PRV: - - + +
Mfn1

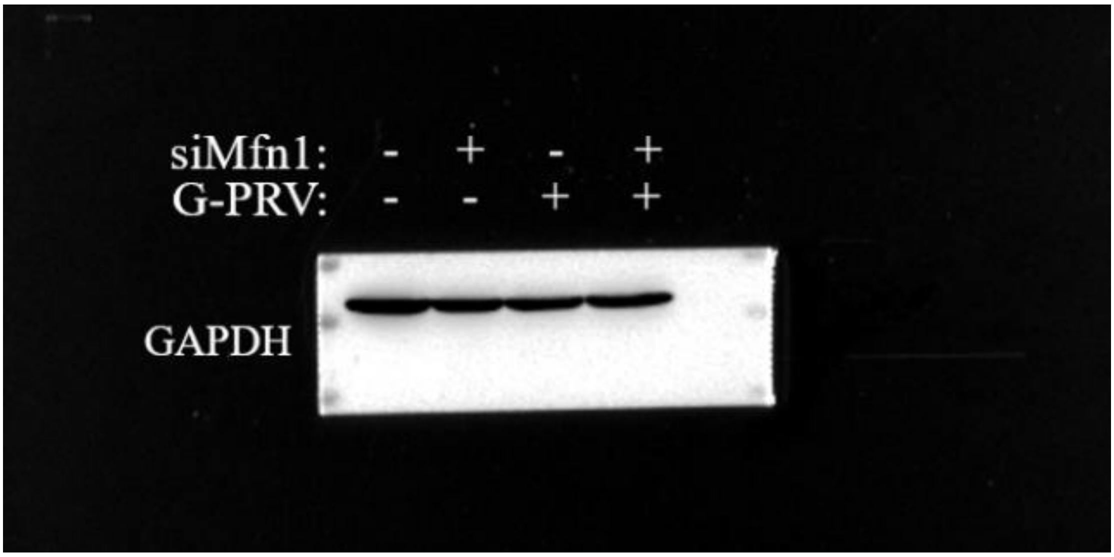
siMfn1: - + - +
G-PRV: - - + +
GAPDH

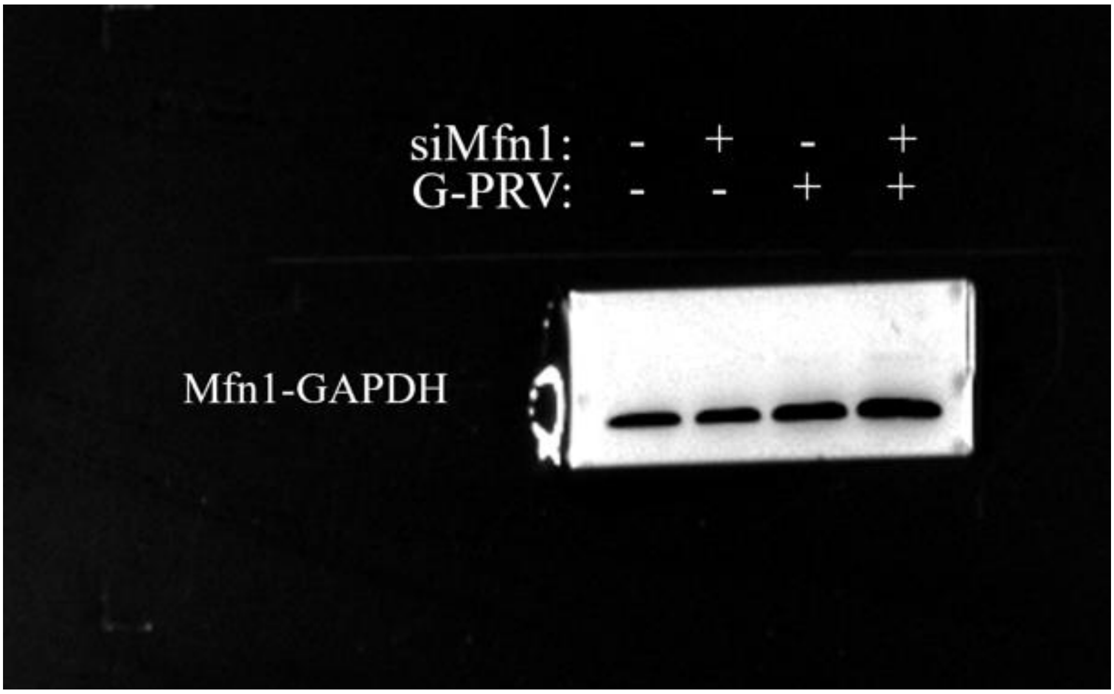
siMfn1: - + - +
G-PRV: - - + +
Mfn1-GAPDH

Figure S5B repeat 2

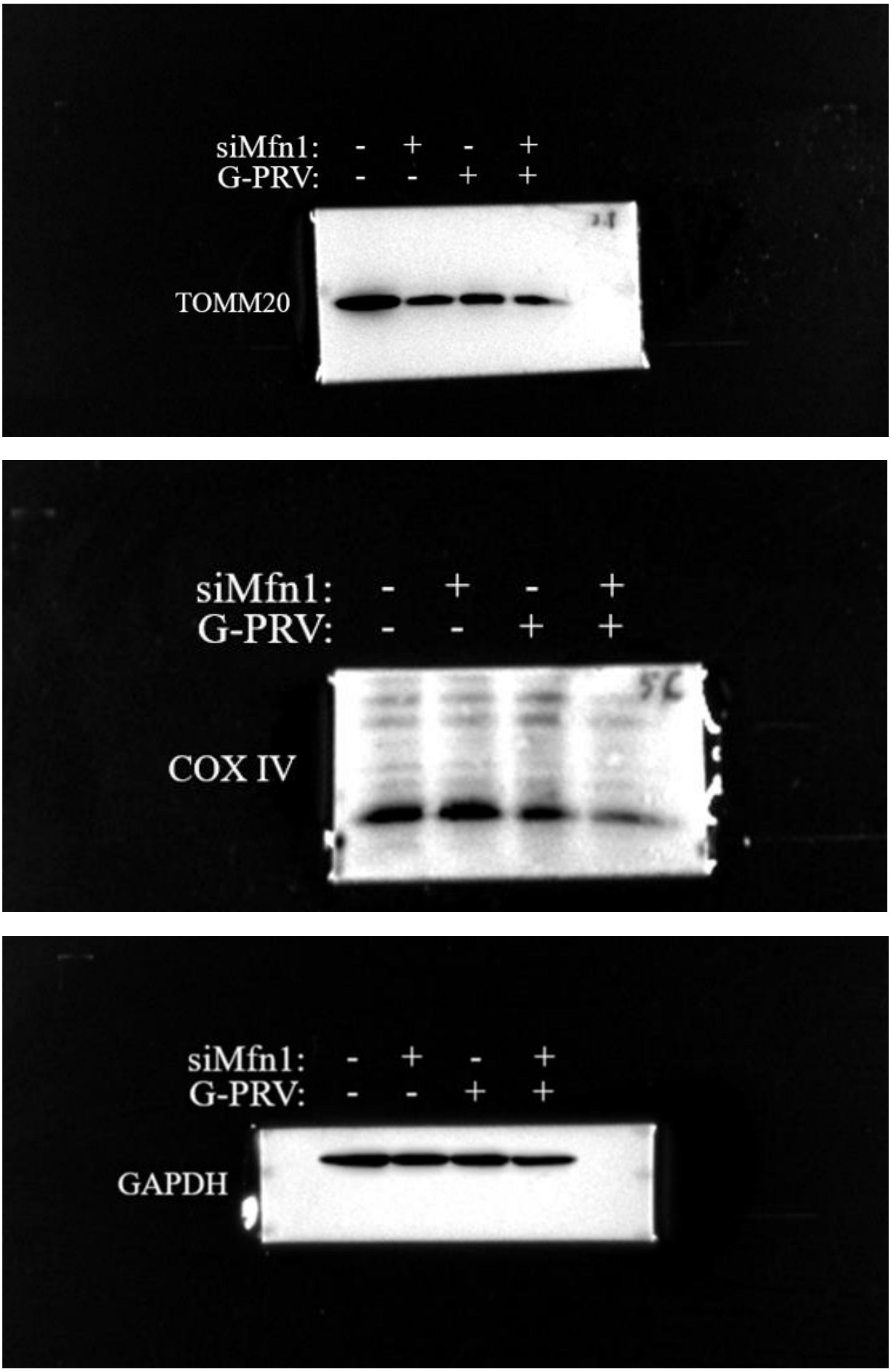

Figure S5B repeat 3

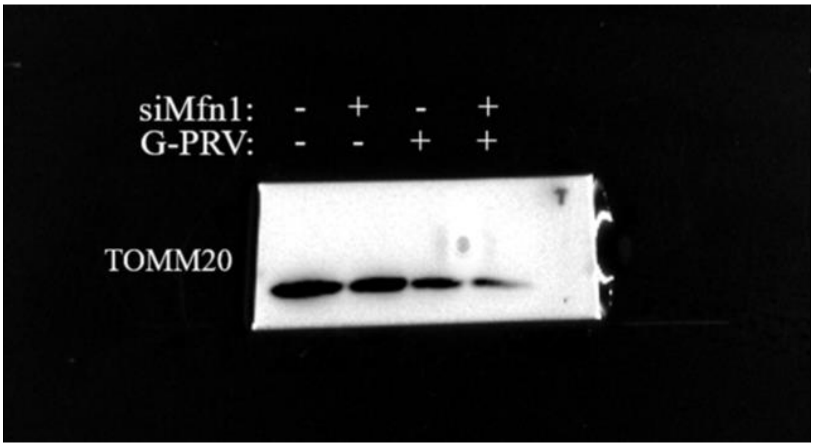

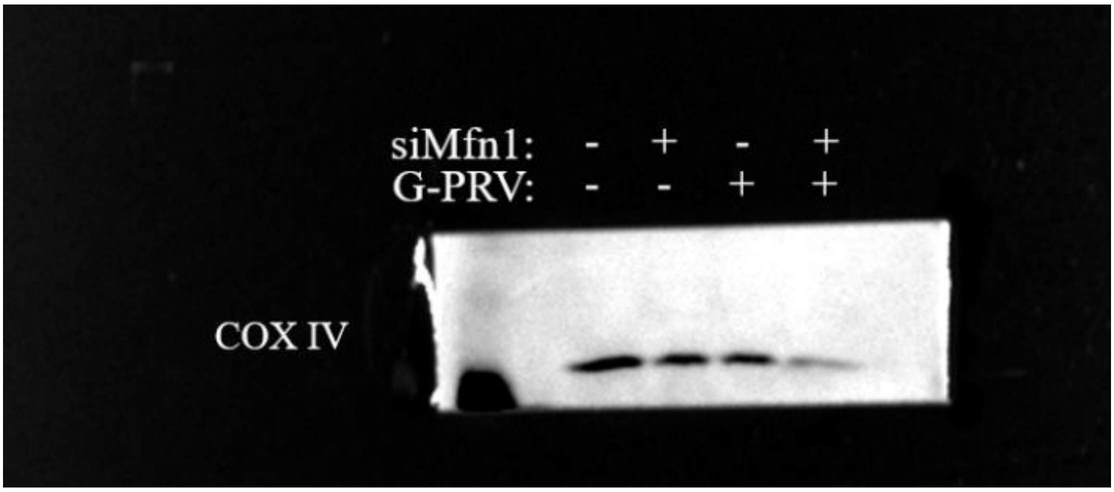

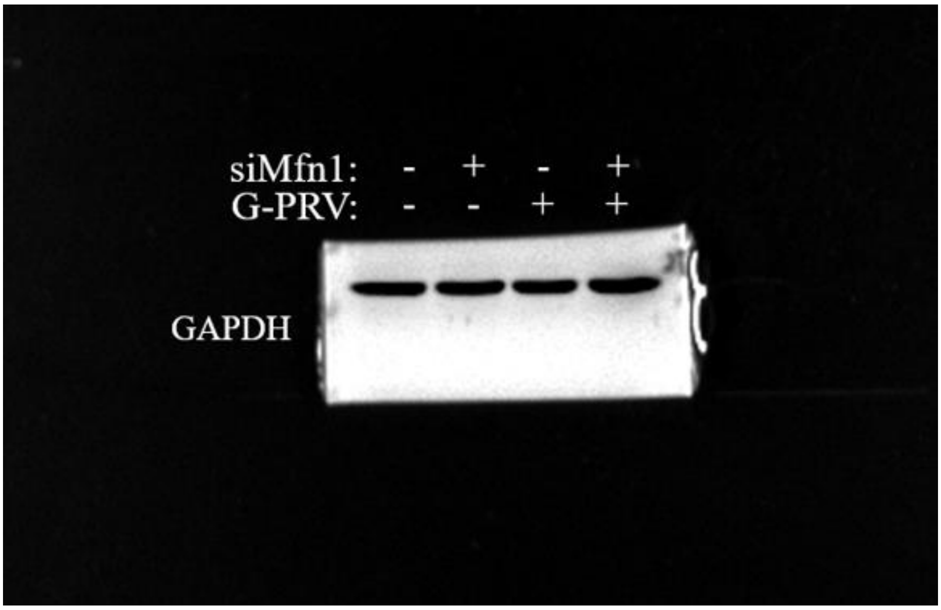

Figure S5C

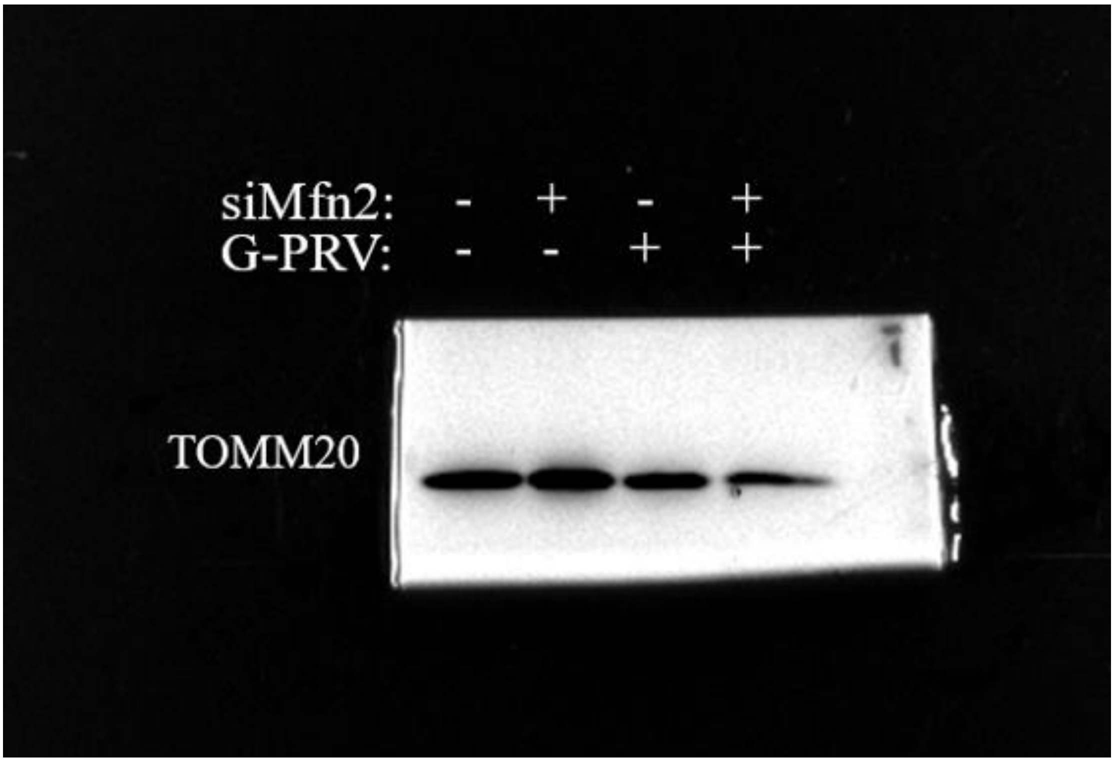

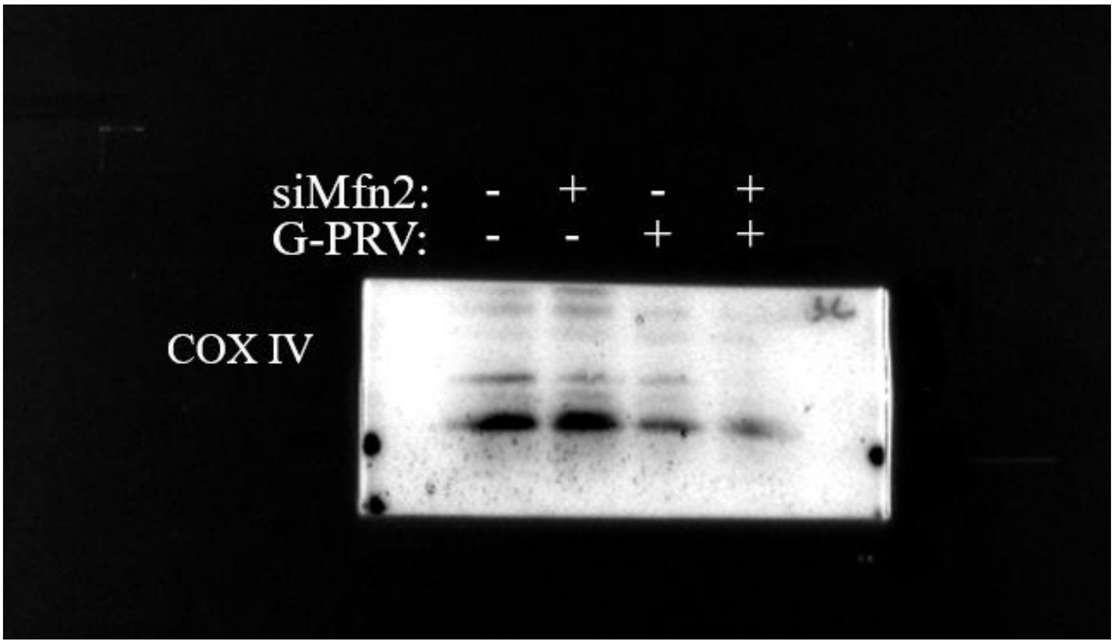

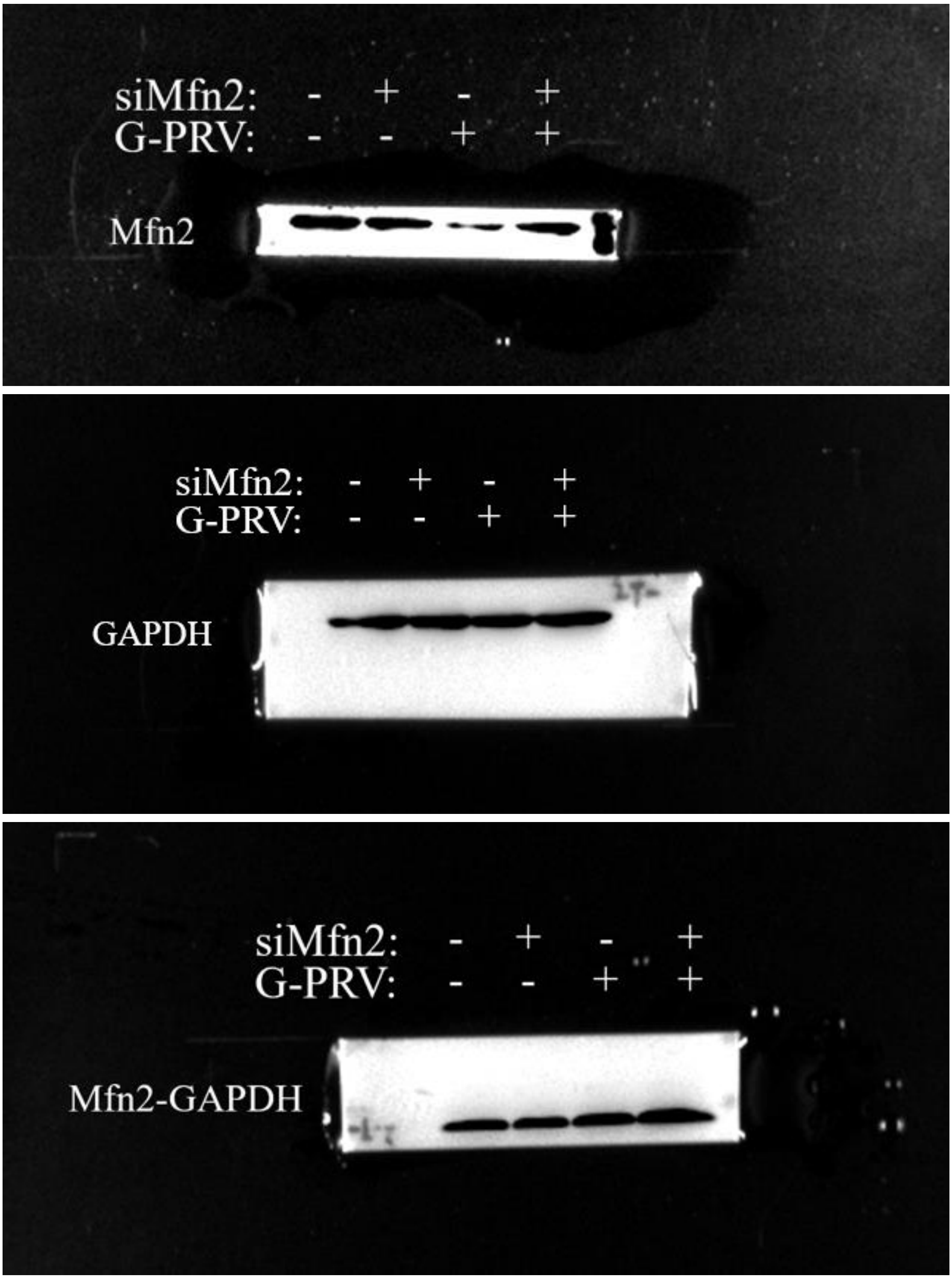
siMfn2: - + - +
G-PRV: - - + +
Mfn2
siMfn2: - + - +
G-PRV: - - + +
GAPDH
siMfn2: - + - +
G-PRV: - - + +
Mfn2-GAPDH

Figure S5C repeat 2

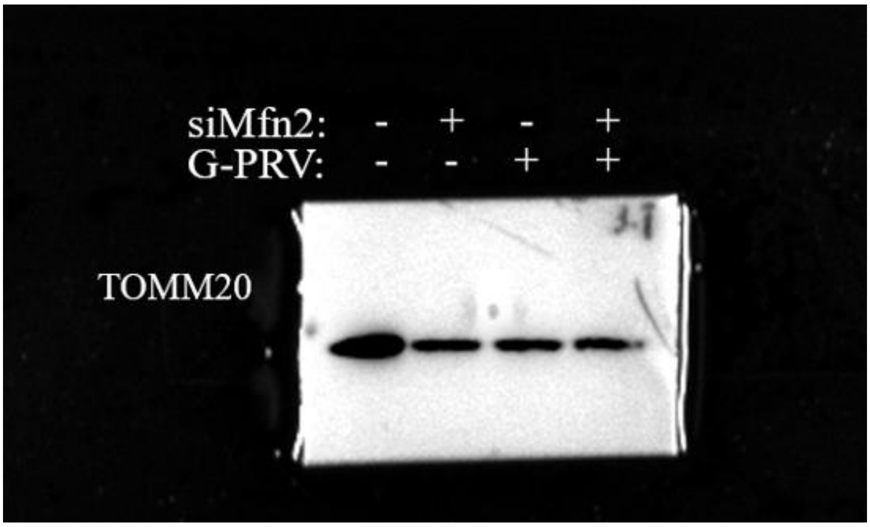

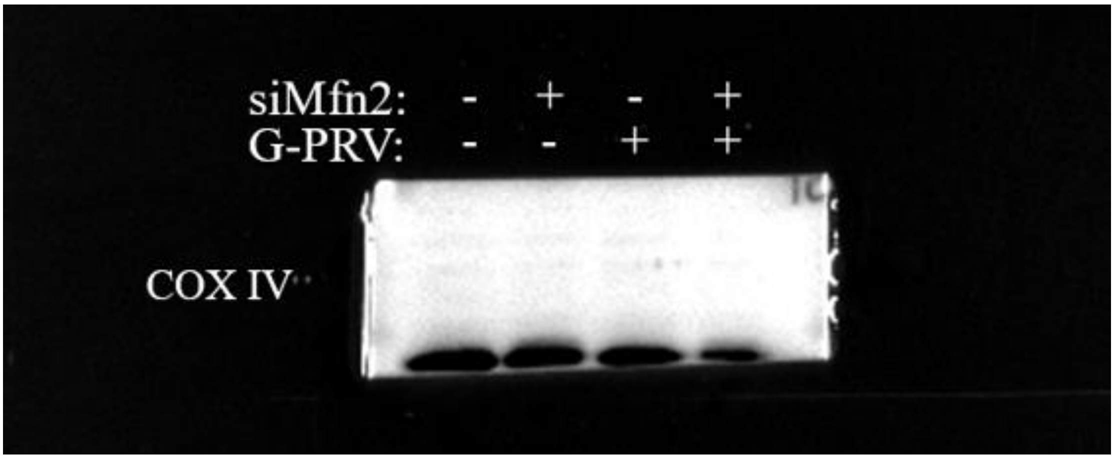

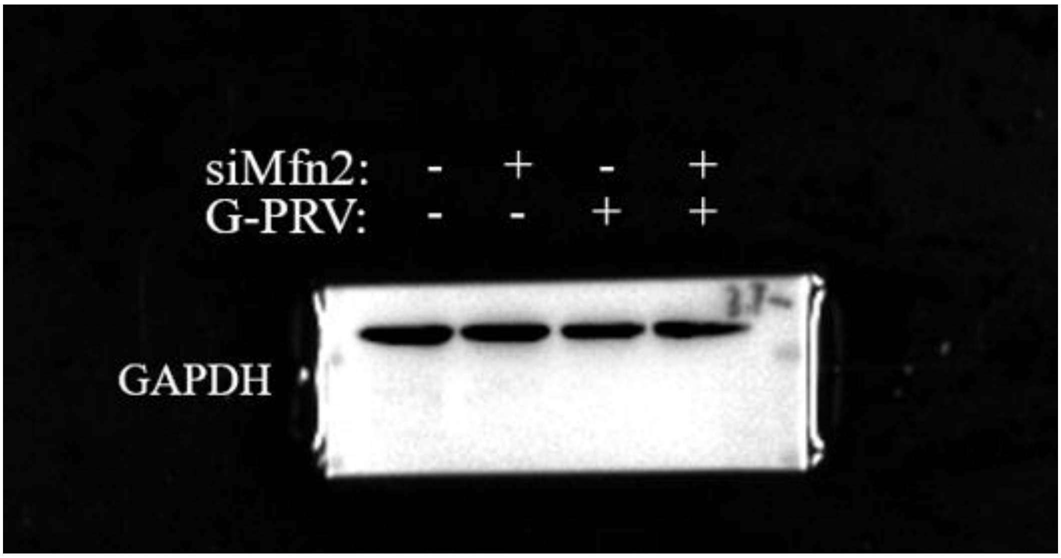

Figure S5C repeat 3

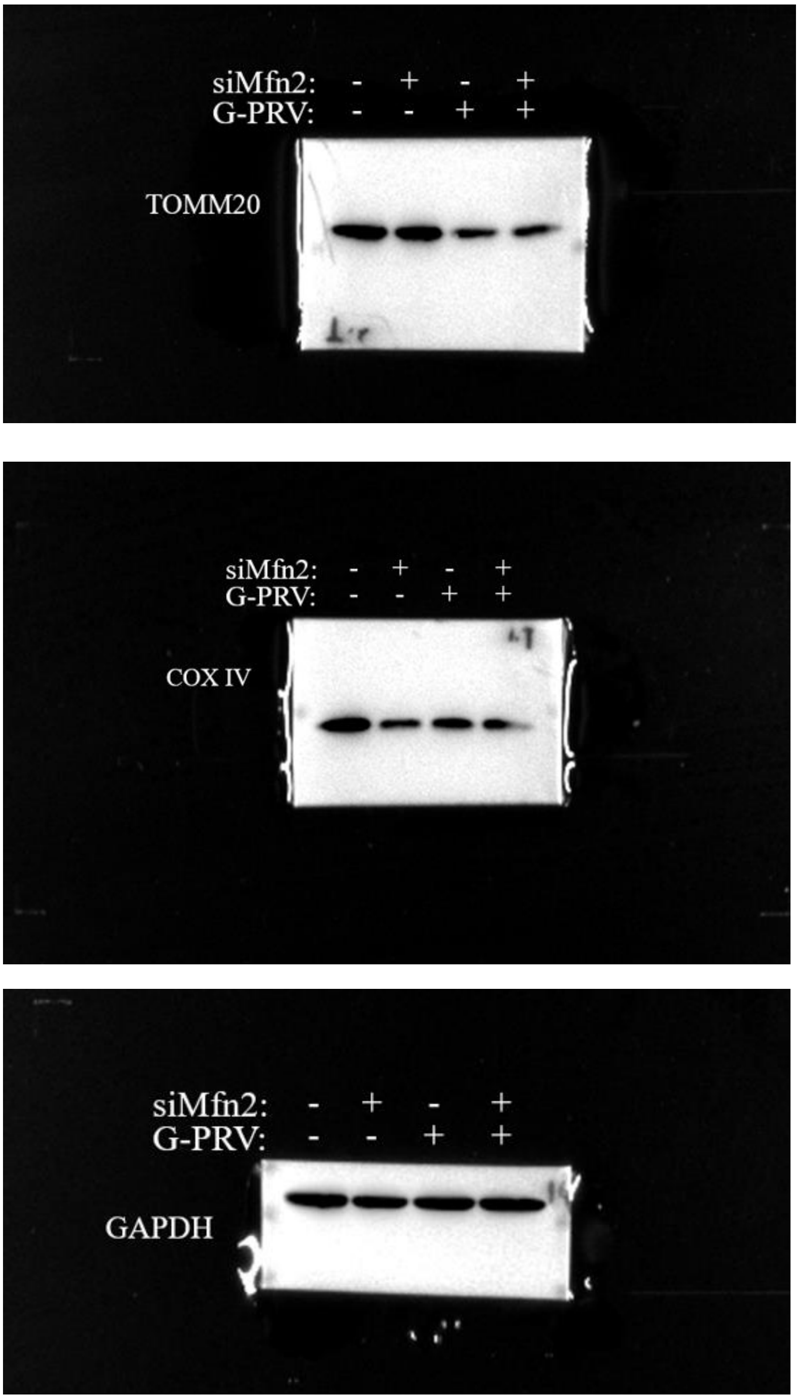

Supplement: Supplementary file 1 [file vetsci-12-00368-s001.zip › vetsci-3517192-supplementary.pdf]
